# Peculiar *k*-mer Spectra Are Correlated with 3D Contact Frequencies and Breakpoint Regions in the Human Genome

**DOI:** 10.3390/genes15101247

**Published:** 2024-09-25

**Authors:** Wisam Mohammed Hikmat, Aaron Sievers, Michael Hausmann, Georg Hildenbrand

**Affiliations:** 1 Kirchhoff-Institute for Physics, Heidelberg University, INF 227, 69117 Heidelberg, Germany; wisam.hikmat99@gmail.com (W.M.H.); aaron.sievers@med.uni-heidelberg.de (A.S.); 2 Institute for Human Genetics, University Hospital Heidelberg, INF 366, 69117 Heidelberg, Germany; 3 Faculty of Engineering, University of Applied Science Aschaffenburg, Würzburger Str. 45, 63743 Aschaffenburg, Germany

**Keywords:** *k*-mer, sequence analysis, Hi-C, 3D genomics

## Abstract

Background: It is widely accepted that the 3D chromatin organization in human cell nuclei is not random and recent investigations point towards an interactive relation of epigenetic functioning and chromatin (re-)organization. Although chromatin organization seems to be the result of self-organization of the entirety of all molecules available in the cell nucleus, a general question remains open as to what extent chromatin organization might additionally be predetermined by the DNA sequence and, if so, if there are characteristic differences that distinguish typical regions involved in dysfunction-related aberrations from normal ones, since typical DNA breakpoint regions involved in disease-related chromosome aberrations are not randomly distributed along the DNA sequence. Methods: Highly conserved *k*-mer patterns in intronic and intergenic regions have been reported in eukaryotic genomes. In this article, we search and analyze regions deviating from average spectra (ReDFAS) of *k*-mer word frequencies in the human genome. This includes all assembled regions, e.g., telomeric, centromeric, genic as well as intergenic regions. Results: A positive correlation between *k*-mer spectra and 3D contact frequencies, obtained exemplarily from given Hi-C datasets, has been found indicating a relation of ReDFAS to chromatin organization and interactions. We also searched and found correlations of known functional annotations, e.g., genes correlating with ReDFAS. Selected regions known to contain typical breakpoints on chromosomes 9 and 5 that are involved in cancer-related chromosomal aberrations appear to be enriched in ReDFAS. Since transposable elements like ALUs are often assigned as major players in 3D genome organization, we also studied their impact on our examples but could not find a correlation between ALU regions and breakpoints comparable to ReDFAS. Conclusions: Our findings might show that ReDFAS are associated with instable regions of the genome and regions with many chromatin contacts which is in line with current research indicating that chromatin loop anchor points lead to genomic instability.

## 1. Introduction

The cell nucleus, containing chromatin (DNA packed by proteins), RNAs, proteins, enzymes, water, ions, etc., is a self-organizing, non-random system [1], i.e., all these components arrange in a well-defined way depending on laws of mechanics (e.g., stiffness of chromatin), electrostatics (e.g., electrostatic potentials around charged units), or thermodynamics (e.g., entropy, diffusion) [2,3]. The principles of organization reveal gene-rich chromosomes in the nuclear center and gene-poor ones in the periphery [4]. This means that certain chromosomes and chromosome domains are located in adjacent neighborhoods [5]. Hi-C experiments [6,7] provide indirect insights into the 3D conformation of chromatin by deriving pairwise contact frequencies between nearly all possible DNA positions [8]. In recent years online databases were created, allowing simple and free access to contact datasets for many cell lines and species.

Another consequence of this defined neighboring can be seen in tumor development, especially in hematological tumor diseases, where the formation of structural chromosome aberrations is of causal importance. If under environmental stress of chromatin (e.g., ionizing radiation exposure, toxic chemical treatment, etc.) or intrinsically during cell metabolism, the dedicated DNA-repair mechanisms fail or their results are deficient, resulting DNA breaks being wrongly attached to adjacent regions of another chromosome in close vicinity which may lead to pathogenic mutations, cell death, or tumorigenesis. Under such stress, a DNA break, for instance, within the ABL gene on chromosome 9, and the consecutive translocation of the fragment to the neighboring BCR gene locus on chromosome 22 lead to the harmful BCR-ABL fusion [9] resulting in an extremely shortened chromosome 22 (Philadelphia chromosome) [10] and a modified variant of chromosome 9 fused with the remaining parts of chromosome 22. The translation of the BCR-ABL fusion gene produces a potentially pathogenic protein that plays a major role in oncogenesis of leukemia. The Hap1 cell line [11] used as a model in this study was derived from a human chronic myeloid leukemia tumor and possesses the described Philadelphia chromosome translocation variant [12].

Besides environmental factors like ionizing radiation and mutagenic chemicals, the DNA sequence context of the potential DNA breakpoints and the 3D environment within the cell nucleus might be risk factors for DNA breaks. For instance, in cell nuclei of patients suffering from the myelodysplastic syndrome (MDS), a precursor of leukemia (e.g., acute myeloid leukemia), chromosome translocations are frequently occurring with chromosome 5 where the breakpoints are always found within a small banding region [13]. 

These examples not only motivate studies on chromatin organization and neighboring regions using Hi-C technologies [6,7,14] or (super-resolution) fluorescence microscopy [15] but also pattern and motif searches in sequence databases in order to find out whether in those regions involved in chromosome aberration formation defined sequence conditions exist that deviate from the rest. Recent studies using non-alignment-based comparison methods (*k*-mer-based methods), sensitive to the sequence context, applied to eukaryotic genome sequences led to the discovery of an overabundance of certain repeat structures, especially A+T-rich super-short tandem repeats (SSTRs) in non-coding sequences [16,17]. Since a clear functional annotation for the observed patterns is still missing and the sequence context is known to influence the physical properties of DNA (and chromatin) [18,19] as well as the binding affinity for certain proteins [20,21], an effect on chromosomal stability seems reasonable, especially since properties related to chromatin stability, namely the histone affinity, and therefore stiffness (e.g., persistence length) and packaging of chromatin, are known to be strongly affected by A+T-rich DNA repeats [22].

Histone occupancy is also believed to influence the 3D conformation of chromatin which is another potential risk factor for DNA breaks and fusion events [23], since DNA molecules can only fuse if they come into physical proximity/contact. Recent studies also independently found a relation between DNA repeats and 3D conformation. These studies included tandem repeats (TRs) but mainly found transposons, especially Alu, as potential players for the chromatin 3D organization in humans [24,25]. Alu is a very frequent (e.g., covering ~10% of the human genome) around 300 bp long retrotransposon (SINE) present in genomes of primates. 

In this study, we focused on Hi-C data from Hap1 cells, since the fact that they were nearly haploid [11] simplifies the interpretation of Hi-C datasets, which normally cannot differentiate between homologous chromosomes, which could otherwise potentially influence the detection of the translocation variant on chromosome 9, by adding noise from alleles with different sequences and locations within the nucleus. Hi-C experiments have already been used to identify functional 3D chromatin structures like A/B compartments and transcriptional insulation neighborhoods called topologically associated domains (TADs) [26,27], sometimes also referred to as contact domains, since most 3D contact detected by Hi-C experiments is between sequence parts within the same TAD [26,28].

For the first time, we search for relations between the DNA sequence context, DNA breakpoints, and chromatin 3D conformation. We present and apply a *k*-mer-based (alignment-free) algorithm to identify some typical regions with peculiar DNA sequence structures. DNA word spectra of these regions are analyzed to identify different classes of regions, based on DNA word patterns. These classes are compared with DNA breakpoint regions, protein coding regions, and transposons. Finally, we search for a correlation between DNA word spectra and 3D contact frequencies from Hi-C experiments.

## 2. Materials and Methods

### 2.1. Data Sources

In the presented study, we used sequence data from the human genome GRCh38.p13 Primary Assembly (downloadable from the NCBI website [29]).

Hi-C datasets used were retrieved from Genome Interaction Tools and Resources (GITAR) under https://www.genomegitar.org/processed-data.html (accessed on 3 September 2020) [30]. The cell lines used were the lung fibroblasts IMR90 (CCL-186) [31] (accession no. GSM1551599) and the haploid fibroblast-like Hap1 (accession no. GSM1909121) [32].

### 2.2. k-mer Analysis

*k*-mer words (k bases long sequences) were obtained by a sliding window approach. The frequencies for DNA words of length *k* were derived from the chromosomal DNA sequences using the Oligo software package [17]. The collection of DNA word frequencies derived from a sequence is referred to as the associated *k*-mer spectrum [33]. *k*-mer spectra can be interpreted as 4^k^-dimensional vector representations of the associated sequences and therefore be compared by using established vector metrics, e.g., the *Pearson correlation function* [34]. Accordingly, the result of such a comparison was interpreted as a measure of similarity of associated sequences (see [33] for details of the method). While in general the word size *k* can be chosen arbitrarily, the most effective tradeoff between information content and computational time was achieved by choosing a word size *k* based on the sequence length *n*, using the formula $k=0.7\;{log}_{4}\left(n\right)$ [35].

### 2.3. Local k-mer Analysis

In a standard *k*-mer analysis, as described above, only the frequencies of DNA words are saved while information on their relative positions in the sequence is lost. While this data reduction is the key difference between more conservative approaches (e.g., alignment algorithms) and the application described here, positional information is crucial in order to find locally annotated sequence patterns. Therefore, we re-included the positional information (partially) by splitting the sequences into segments of equal size and derived *k*-mer spectra for each segment as if they were independent sequences. The resolution for identifying peculiar *k*-mer features was dependent on the size of these segments. In general, on one hand one would prefer a high resolution and therefore the chosen segment size should be very small. On the other hand, smaller segment sizes increase computational times and imply fewer DNA words per spectrum. This leads to a potential loss of statistical significance of the results. Accordingly, the lower limit of the segment size was given by the requirement of a minimum of a few dozen counts per segment for each DNA word [36].

A measure of peculiarity of a local spectrum was derived by pairwise comparing the *k*-mer spectrum of the segment with every *k*-mer spectrum associated with another segment (approach 1). Since this derivation was very time consuming, requiring the pairwise correlation of thousands of spectra with thousands of components each, we decided to use a less complex approach. We compared each segment’s (local) *k*-mer spectrum with only one reference spectrum, namely, the average (chromosomal) *k*-mer spectrum (approach 2). We defined the deviation from the average spectrum as the sum of the differences between frequencies of individual DNA words i in the local *k*-mer spectrum $f_{local_{i}}$ and the reference *k*-mer spectrum $f_{ref_{i}}$ (see Equation (1)).

(1) $$d\;=\;\sum_{i}f_{local_{i}}-f_{ref_{i}}$$

This approach required only one correlation for every segment. In order to compare the two approaches, we compared the average correlation value from approach 1 with the result from approach 2 (see Figure S1). The results were very similar (Pearson correlation of −0.96). Accordingly, we decided to use the deviation from average spectra (approach 2) for this study.

The similarity/deviation of the local *k*-mer spectrum with/from the average *k*-mer spectrum could then be correlated with the density of the other annotated elements (e.g., genes, transposons, DNA breakpoints) to find associations and thus gain insights into potential functional relationships between observed patterns and these elements. This comparison introduced another limitation for the segment size, since annotations themselves often have a limited resolution, e.g., Hi-C data used in this study have a typical resolution of 40 kbp only [30].

### 2.4. Deviation from Average Spectra for Word Sets

While, in general, the sum in Equation (1) was derived over all DNA words within a spectrum, it was possible and meaningful to take the sum over a predefined subset of DNA words to derive the influence of this DNA word set on the deviation from average spectra. In this work, we define different word sets associated with different sequence pattern of words to identify patterns relevant for the deviation.

### 2.5. Regions Deviating from Average Spectra (ReDFAS)

Dividing chromosomes into segments as described above produced one spectrum for each segment. In order to find regions with peculiar *k*-mer patterns, deviating from the average spectra (ReDFAS), a quantification of peculiarity of *k*-mer spectra was needed. We defined a ReDFAS as a region (a region can be larger than one segment) that has a deviation from average spectrum (see Equation (1)) larger than a certain threshold d_threshold_. Here, d_threshold_ was chosen in such a way that only 5% of all segments showed values compatible with the parts of ReDFAS. Accordingly, a ReDFAS was a region with an average deviation from the average spectrum higher than 95% of all segments.

### 2.6. Significance of Correlations

We pairwise correlated deviations from average spectra of segments with associated average Hi-C contact frequencies derived for Hap1 and IMR90 [37,38] cells on individual human chromosomes using a sampling (bootstrapping) algorithm for error approximation. In each of 100 repetitions, 100 regions (40 kbp in size) were randomly selected and annotated values (average spectrum deviations of the region and Hi-C contact frequencies) were correlated using the *Pearson correlation coefficient* [34]. Consecutively, we derived mean correlation values and standard deviations for error approximation. Additionally, we calculated reference values for significance tests of the correlations. Therefore, we repeated the sampling and correlation described above for randomly shuffled segments. We considered a correlation value as significant if the absolute differences between mean correlation values from empirical data and reference data were larger than 1*σ* with $\sigma^{2}=\sigma_{empirical}^{2}+\sigma_{reference}^{2}$, where $\sigma_{empirical}$ and $\sigma_{reference}$ were the standard deviations of correlations for empirical and reference data, respectively.

We considered the differences between mean correlation values (e.g., derived from Hap1 and IMR90 datasets) as significant if they were larger than the combination of standard errors of the respective mean values $\sigma_{\bar{x}}=\sqrt{\frac{\sigma_{Hap1}^{2}}{\sqrt{100}}+\frac{\sigma_{IMR90}^{2}}{\sqrt{100}}}$, where $\sigma_{Hap1}$ and $\sigma_{IMR90}$ were the standard deviations of correlations for the respective datasets and 100 was the number of correlated samples.

### 2.7. Principal Component Analysis (PCA)

To perform the PCA on the *k*-mer spectra, we used the PCA implementation within the Oligo software package [17]. We generated a matrix based on the complete *k*-mer spectra for each segment on the respective chromosome and derived the first 3 principal components using default parameters.

## 3. Results

### 3.1. Translocated Regions on Chromosome 9 Are Visible in Hi-C Data

**Figure 1:**
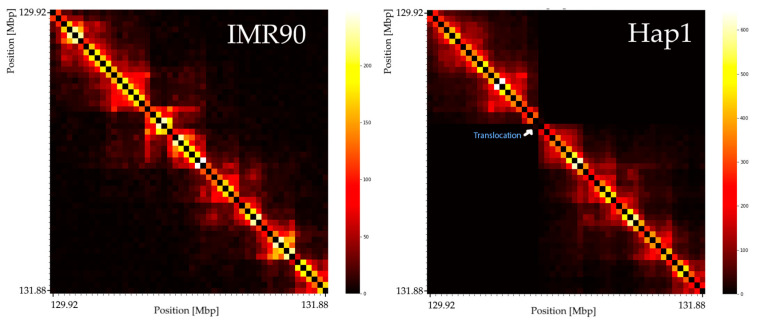
Hi-C contact frequencies (40 kbp resolution) of chromosome 9 at the ABL region is dependent on the examined cell type. Shown are 2 Mbp around the ABL region. Left: IMR90 cell line without ABL-BCR translocation. Right: aberrant Hap1 cell line (with translocation chromosome); ABL region after translocation with BCR. The reduced contacts (dark regions) expected between both sides of the breakpoint are clearly visible for Hap1.

The Philadelphia chromosome and the creation of the BCR-ABL fusion gene is one of the most famous translocations directly involved in oncogenesis of leukemia [39]. The t(9;22) (q34;qll) translocation implies that the part of chromosome 9 that follows downstream the breakpoint in the ABL region should not be in proximity to the upstream part, therefore no further 3D contacts were expected. On Hi-C heatmaps of cells with rows and columns ordered by the relevant positions in a reference genome (i.e., a genome without a break causing a translocation) this should appear as a black low-frequency triangle with sharp borders in the breakpoint region. We compared the Hi-C contact frequencies of the healthy (no breaks in the ABL or BCR region) cell line with the Hi-C dataset of the cell line Hap1 (known to have an ABL-BCR fusion = Philadelphia chromosome) and observed the expected triangle-like structure at the relevant position (Figure 1).

### 3.2. Local Deviations from the Average k-mer Spectrum

We derived the deviations from average *k*-mer spectra for each human chromosome with a resolution of 40 kbp and *k* = 5 (see Figure 2 and Figures S2–S23). In all cases, significant deviations were registered at the centromeres and around the telomeres. In order to show whether such deviations are also typical for breakpoint regions, we analyzed results for chromosome 9 especially in the ABL region (Figure 2a). ABL revealed the typical characteristics of a ReDFAS. 

**Figure 2:**
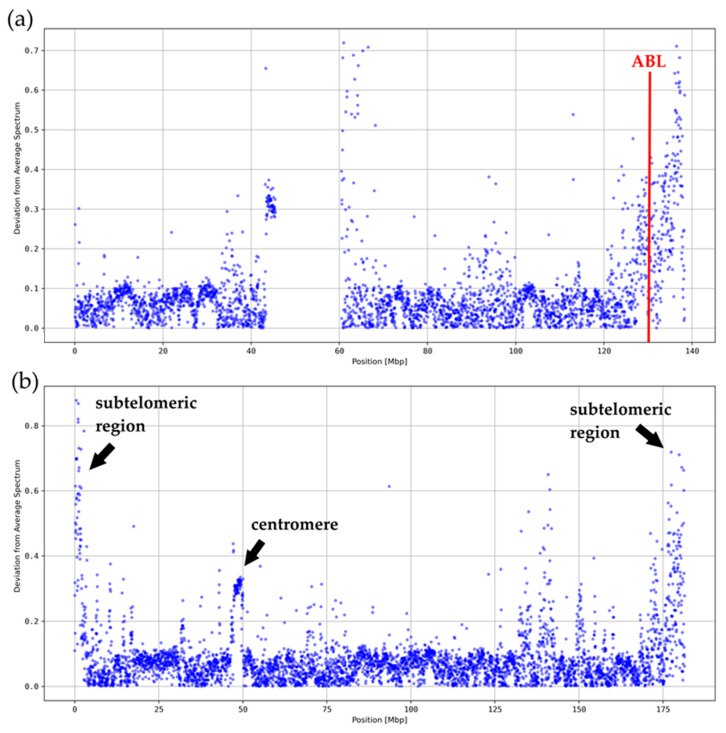
Average *k*-mer spectrum deviation of each 40 kbp segment (for *k* = 5) (**a**) on chromosome 9; ReDFAS are visible in subtelomeric regions, at the centromere (note: missing values from 35–60 Mbp were caused by a sequencing gap), and in the ABL region; (**b**) on chromosome 5; ReDFAS are visible in subtelomeric regions, at the centromere, and clustered in a region at 125–155 Mbp (intermediate ReDFAS).

However, since ABL is located close to the telomere or subtelomeric region, which might have an impact on the deviations in the *k*-mer spectrum, we analyzed chromosome 5 (Figure 2b) which is known to also have a chromosome region—but on the long arm separated from the teleomere—where usually several breakpoints for aberrations relevant in myelodysplastic syndrome (MDS) are located. Again, a typical deviating region, i.e., ReDFAS, on the long arm is visible. In contrast to chromosome 9, where the ReDFAS is associated with one translocation with a major breakpoint and only a few minor breakpoints, the ReDFAS on chromosome 5 seems to be different concerning the genetic outcome. It is associated with multiple chromosome aberrations occurring in MDS which may individually but not simultaneously occur in the disease. Besides different translocations as results of breakpoints, also a deletion could occur in the ReDFAS-rich region on chromosome 5 (del(5q)).

Chromosome 5 is an illustrative example, since it has nearly no sequencing gaps, it is of intermediate size compared to other human chromosomes, and has an average content of coding sequences. The average value of the deviation on chromosome 5 is around 23.4% (see Figure 2b). The large variability in *k* = 5 spectra on chromosome 5 is apparent when looking at the range of different values for the local spectral deviations. This range results from a subset of locally concentrated clusters of segments with very high spectral deviations above 50%. Again, one of those local clusters is associated with the centromere (centromeric ReDFAS), e.g., at 46.5–50.1 Mbp (see Figure 2b). It shows deviations up to more than 100% of the average value. The segments at the beginning of the p-arm and the end of the q-arm (subtelomeric ReDFAS) also consistently show deviating spectra as for most chromosomes. The cluster at 125–155 Mbp (intermediate ReDFAS) also shows such large deviations (see Figure 2b). Since there is no obvious explanation like centromere or telomeres, it could be of relevance for the breakpoint formation.

### 3.3. Classification of ReDFAS

In order to go into more detail regarding the detected ReDFAS, we looked at features of the spectra that differentiate ReDFAS from the remaining chromosomal segments and can be characterized by associated *k*-mer word patterns. Short tandem repeats (TRs), DNA words with repetitive nucleotide patterns and small repeat units, were found to dominate *k*-mer spectra of eukaryotic genomes [16,33]. Thus, we decided to define sets of TR DNA words, which we call A-/C-/G-/T-rich (20 combinations each, one mismatch allowed) and AT-/CG-rich (32 words each, no mismatch allowed). Details can be found in Table 1.

We derived the average deviation from *k*-mer spectra for those word sets for all segments (*k* = 5, segment size 40 kbp) on chromosome 5 (Figure 3). See Figures S24–S46 for other chromosomes.

The deviations of the word sets in individual ReDFAS segments can be very large, up to several hundred percent, and are thus even higher than the overall deviation of the segment. While some differences were visible, the general tendencies of deviations based on C-rich, G-rich, and GC-rich word sets are very similar over the whole chromosome (see Figure S116). The same observation can be made for A-rich, T-rich, and AT-rich ones, while between the two classes of word sets an anti-correlation was observed. This classification scheme is also supported by PCA based on all *k*-mer words (Figure 4 and Figures S47–S69).

The PCA results indicate the existence of a separated cluster of ReDFAS near the centromere and a class of segments that show a continuous pattern from no ReDFAS over to subtelomeric and intermediate ReDFAS (see Figure 4). Intermediate ReDFAS seem often to lie between the other subsets, rarely differing (see Figure S54 for chromosome 9).

Since the G+C content of *k*-mer words seems to be an essential element for the classification of ReDFAS, we searched for a general dependency of the spectral deviation and the local G+C content. In general, the G+C content shows less variance over all segments as seen in Figure 5 and Figures S70–S92.

**Figure 3:**
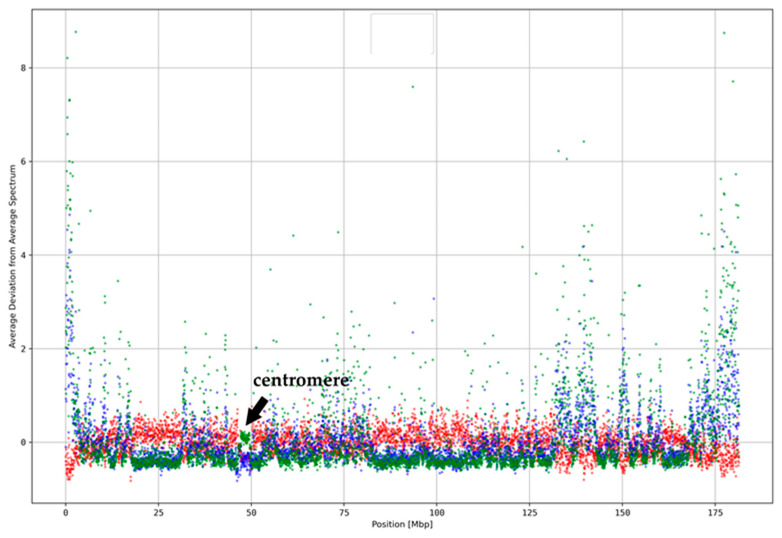
The spectral deviations of segments on chromosome 5 for a selection of representative word sets (see Table 2). 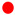 = A-rich; 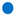 = C-rich; 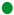 = GC-rich.

**Figure 4:**
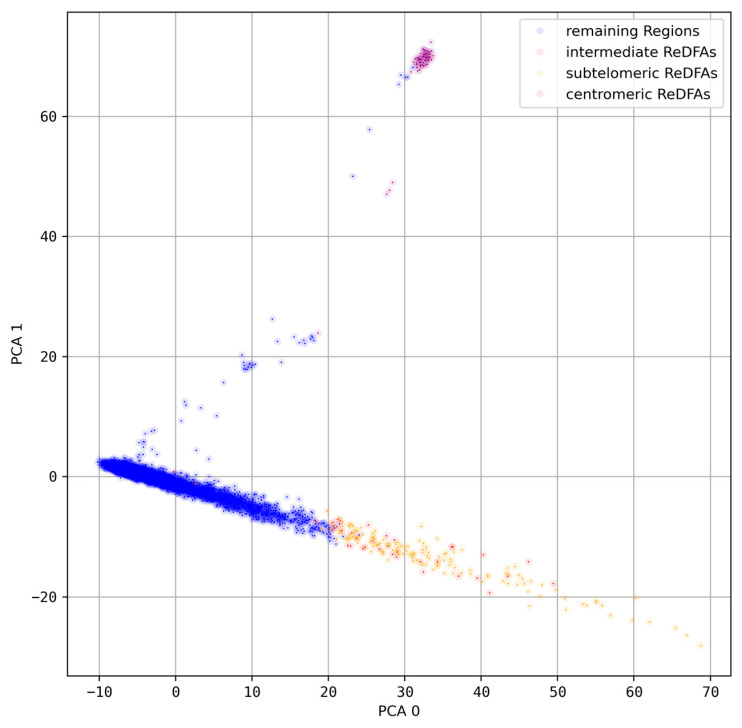
PCA results *k* = 5 on chromosome 5: PCA was performed on *k* = 5 spectra of 40 kbp segments on chromosome 5. Different classes of regions are labeled with different colors. Red: intermediate ReDFAS without special label; yellow: subtelomeric ReDFAS; purple: centrometic ReDFAS; blue: segments not labeled as ReDFAS.

**Figure 5:**
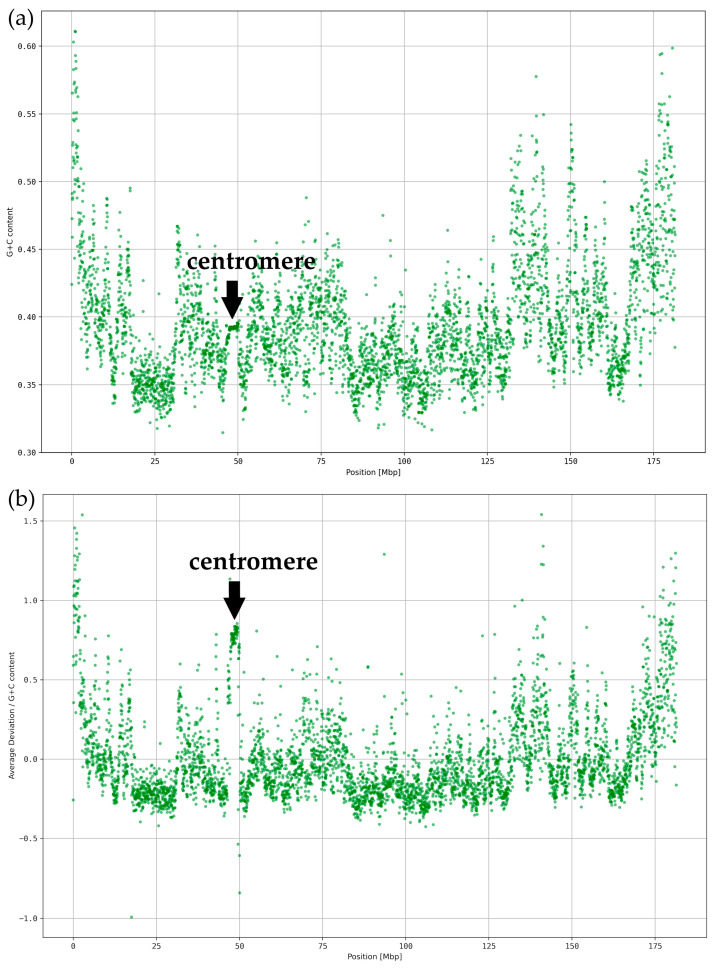
Relation between ReDFAS and G+C content. (**a**) G+C content of 40 kbp segments on chromosome 5. (**b**) Average deviation from average spectra of 40 kb segments on chromosome 5, corrected for local G+C content.

The drop in C-rich and G-rich words in the centromeric region in Figure 3 is not visible as a drop in the local G+C content (see Figure 5a), while the G+C content is slightly higher in the subtelomeric regions and the chr5: 125–155 Mbp region. The correlation coefficient between average spectrum deviation (Figure 3) and the G+C content (Figure 5a) is 0.56 which may indicate a weak to intermediate dependency. This weak relationship was expected since variances in DNA word frequencies with unequal G+C to A+T contents (e.g., AAAGG) should influence local G+C content. We corrected the average deviation from average spectra within the 40 kbp segments for the local G+C content (see Figure 5b). All patterns observed without correction, especially the concentration of ReDFAS near centromeres and telomeres as well as the chr5: 125–155 Mbp region, were more clearly observable after the correction. The correlation coefficient between uncorrected (Figure 3) and corrected results (Figure 5b) is 0.99, supporting the observation that the reason for the changes in the pattern could not be totally explained by local G+C content. We conclude that changes in the local G+C content cannot only explain the observed higher abundancies of word classes in ReDFAS.

### 3.4. Relationship between k-mer Spectrum Deviation and 3D Chromatin Organization

The observed local sequence patterns could have an impact on the 3D organization of chromosomes, e.g., by altering sequence-dependent physical properties of chromatin. These could, e.g., induce conditions that favor DNA damage and thus single- and/or double-strand breaks. The differences of Hi-C contacts between Hap1 and IMR90 cells were evaluated (see Figure 6 and Figures S93–S115) to see how Hi-C data differ between different cell lines from different tissues. As expected, Hi-C frequencies were highly conserved between different human tissues/cell lines (Pearson correlation: 0.787, Figure 6).

**Figure 7:**
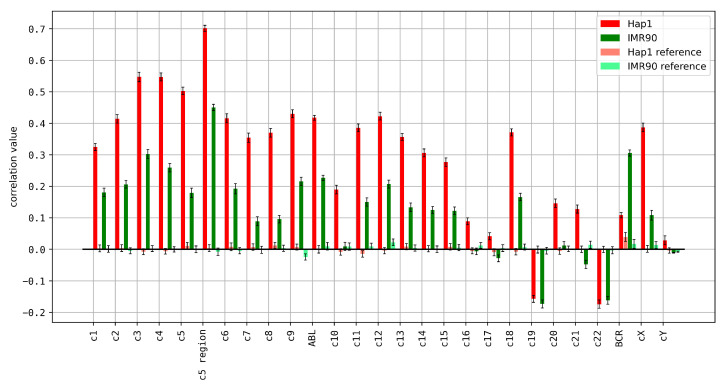
The correlation of Hi-C contact frequencies with *k*-mer spectrum deviation for each chromosome (excluding centromeres), c5: 125–150 Mbp (c5 region) and ABL as well as BCR breakpoint regions. The datasets labeled as reference show correlation data derived for randomly shuffled datasets (see Section 2 Materials and Methods for details).

We compared the observed spectral deviations with 3D chromatin contact frequencies from Hi-C databases. Since Hi-C datasets were lacking data for centromeres, the correlations were performed with masked centromere regions. We found significant correlations between the spectral deviations and Hi-C contact frequencies (Figure 7).

We found a positive correlation between Hi-C data and spectral deviations for 22 of 24 human chromosomes in the cancerous Hap1 cell line, whereas the healthy cell line shows a positive correlation for 19 chromosomes only (Figure 7).

The absolute value of the correlation differs significantly between the two cell lines analyzed (Hap1 and IMR90) for all chromosomes. This indicates that the relation between *k*-mer spectra and 3D chromatin conformation change is dependent on the cell line [40] and cell fate [15]. Some chromosomes show high correlations between Hi-C and spectral deviations, suggesting that the sequence composition might have a different impact on the 3D structure on these chromosomes (Sievers et al., manuscript submitted). This can be further broken down to specific regions like chr5: 120–150 Mbp which is involved in the del(5q) mutation in MDS. The correlation value between average spectrum deviation and contact frequency in this region is high (0.744) in the leukemia cell line Hap1. While ABL and chromosome 9 show high correlations in Hap1 and increased correlations in IMR90, chromosome 22 as a whole shows negative correlations. Since it is a relatively small chromosome with a low number of genes, size and gene density could be relevant factors. However, BCR by itself is positively correlated, especially in IMR90. This may indicate that BCR is the only open part for contacts. In addition, it should be considered that ABL is a protein-coding region and BCR by itself is not. Chromosome 19 as the most gene-rich chromosome also shows a negative correlation since it has nearly no ReDFAS.

### 3.5. Relationship between ReDFAS, Breakpoint Regions, and NPCs, PCs, CDSs, ALUs, and L1s

In order to test our hypothesis that spectral deviations of the DNA sequence have an impact on chromosomal stability in ReDFAS and thus lead to the formation of DNA breaks, we searched for an enrichment of typical disease-related breakpoint (BP) regions in ReDFAS and compared this to other characteristic regions in each chromosome. Since non-protein-coding genes (NPC) and coding sequence (CDS) regions show distinct *k*-mer correlations [3], these regions were considered for BP analysis. Additionally, the interspersed elements ALU and L1 were analyzed, since transposable elements are known to be determinedly involved in genome organization [41]. Thereby, ALU is in the genome often separated from L1 and L1 is often integrated into heterochromatin-dense regions.

In Table 2, the results are summarized. In nearly all cases, but especially for chromosomes 1–16, X, and Y, the relative coverage of the chromosome by the respective genomic feature (ReDFAS, NPC, PC, CDS, ALU, L1) is not associated with the relative amount of breakpoint regions that are found within these features. The amount of BPs inside ReDFAS is more than doubled relative to the value expected from the chromosomal coverage of the ReDFAS. However, chromosomes 17–22 show a different relation regarding BPs found in ReDFAS. Instead of being enriched in ReDFAS they seem to be depleted. Beyond ReDFAS, the highest enrichment of BPs was found in coding sequences (CDSs), i.e., in euchromatin, while nearly no BPs were observed in L1 related to heterochromatin. NPC and ALU showed the opposite to ReDFAS, i.e., the amount of BPs inside NPCs and ALUs is about the half that expected from the chromosomal coverage of these regions. The values for BPs in ReDFAS and in CDSs are highest for chromosome Y.

So, it seems to be that with genetic activity correlated to an improved accessibility of the DNA the occurrence of BPs is increased. Therefore, the accumulation of these genomic features within ReDFAS and the prevalence of BPs within the genomic features was calculated for each chromosome and word set (see Table 2). Breakpoint regions are significantly enriched in ReDFAS and CDSs (see Table 3). Since most breakpoints are found because they lead to diseases [42], the large proportion observable in Table 2 and Table 3 were expected. In contrast, BPs tend to avoid NPCs and ALUs and especially L1 elements, whereas only L1 is significantly rarely found within ReDFAS (Table 3 and Table 4).

## 4. Discussion

In this work, we searched for relations between the DNA sequence context, DNA breakpoints, and chromatin 3D chromatin conformation and showed the outcome especially for chromosomes 9 and 5 since these chromosomes are involved in typical chromosome aberrations known in leukemia and MDS. We presented and applied a *k*-mer-based (alignment-free) algorithm to identify regions with peculiar DNA sequence structures we call ReDFAS. Since the sequence context influences properties of DNA and chromatin-folding features, we searched for association with chromosomal regions with known functions and importance for chromatin organization and found ReDFAS in centromeric and subtelomeric regions of all human chromosomes, being already in regions known for specific 3D arrangements. A minority of ReDFAS are located scattered around chromosomes in local clusters with sizes of around 10 Mbp (intermediate ReDFAS), raising the question of whether these regions might also have a structure-inducing function for chromatin 3D organization. Since DNA context-dependent physiochemical properties were known to influence local binding affinities for proteins (e.g., histone affinity [43]) and therefore stiffness of DNA and chromatin, the underlying mechanism by which peculiar sequence patterns influence chromatin 3D conformation could be a shifting of energetic costs for the formation of functional 3D conformations (e.g., loops, loop anchors).

A classification of ReDFAS, based on the influence of different TR DNA word sets on the variation from average spectra, revealed two classes of ReDFAS. ReDFAS in the first class are associated with centromeric regions and show high influences from TRs with low G+C contents, while ReDFAS in the second class were located in subtelomeric and intermediate regions, with high influences from TRs with high G+C contents. Since an association of ReDFAS and local G+C content was not observed, we conclude that the correlation with different G+C classes of TRs is not simply the result of higher or lower G+C content of ReDFAS classes. Since intermediate ReDFAS show sequence patterns comparable to subtelomeric ReDFAS, a similar mechanism, e.g., chromatin packaging density, or even a similar function (e.g., genomic stability) for these ReDFAS seems to be a reasonable assumption. Another possible explanation for the presence of DNA words with high G+C contents in scattered ReDFAS could be an enrichment of loop anchor points (LAPs). LAPs are relatively G+C-rich and show histone modifications, which coincide with the findings that subtelomeric and intermediate ReDFAS are preferred to be G+C-rich [44]. Since DNA repeats sometimes were mentioned as possible candidates for direct mediators of chromatin contacts [45], the sequences with G+C-rich words (e.g., SSSSS) might directly work as anchor points or indirectly as unspecific binding sites for proteins associated with anchor points [46]. In any case, a higher abundance of loop anchor points should lead to a higher packaging density in ReDFAS which is supported by the observed correlation between the deviation from average spectra of segments (the main property that defines ReDFAS) and Hi-C contact frequencies.

Another mechanism that could explain the correlation between sequence context and packaging density, also including the observation that TRs might be involved, would be the modulation of histone occupancy. Long stretches of A+T-rich repeats influence histone occupancy and therefore physical properties of chromatin like its stiffness (persistence length) [47,48]. Their absence in scattered ReDFAS might therefore indicate higher histone occupancies and accordingly more flexible chromatin regions that can be easily contracted to higher packaging densities and thus change cellular functioning and finally the cell fate.

Other DNA repeats, especially the local density of ALU elements, which have a high G+C content and therefore could also influence the G+C content of ReDFAS, are also known to be associated with Hi-C contact frequency levels [49] and are therefore believed to be of importance for understanding the connection between sequence context and chromatin 3D conformation. Interestingly, we could not observe a correlation between the density of Alu elements and ReDFAS; we observed a depletion of Alu elements in ReDFAS. Since Alu elements and subtelomeric and intermediate ReDFAS share elevated G+C content and correlations with 3D contact frequencies, this might indicate that ReDFAS and Alu elements are two mechanisms for 3D chromatin organization with a special role of their own specific G+C-rich sequence patterns. While a detailed analysis of this hypothesis is out of the scope of this work, the long-known fact that Alu elements are associated with gene-rich regions and the fact that no relation between ReDFAS and Alu elements was observed might indicate that Alu elements play a similar role to ReDFAS but in closer proximity to genes.

Since we also found a significant enrichment of DNA breakpoint regions in ReDFAS, while in contrast we found (consistent with [50]) a depletion of breakpoint regions in transposons (Alu and L1), the influences on DNA and/or chromatin properties by the peculiar sequence context in ReDFAS also seem to affect genome stability, differing from those of Alu elements. It is possible that the sequence context or the resulting binding affinity for proteins (e.g., histones) to DNA increases the risk of DNA/chromatin strand breaks or that ReDFAS are the result of a compensatory mechanism, stabilizing regions that were feasible for DNA breaks. In both cases ReDFAS might be relevant players for the prediction and understanding of various genetic diseases, like cancer. This further supports the hypothesis on the relevance of LAPs since they were known to be highly mutated in various cancer cells [51]. Additional support for the relevance of ReDFAS for cancer is our observation that the correlations between spectral deviations and Hi-C contact frequencies show significant differences when comparing data from cancerous Hap1 and normal IMR90 cell lines. While the analysis of only two cell lines leaves space for other interpretations, e.g., differences in 3D contact frequencies between tissues of different experimental setups, our findings might still be a first hint that the mechanism that associates ReDFAS and 3D contacts is disturbed in cancer cells.

The general findings of correlations of ReDFAS with already known functional elements and regions suggest their own function in the genome. The specific association of ReDFAS with breakpoint regions also implies clinical relevance worth further research. Especially, the example of chromosome 5 where the ReDFAS seem to be associated with multiple aberrations in MDS needs further investigations. It may be useful to increase the resolution in this special region under the aspect that deletions and translocations occur in this region but with very different chromatin breakpoints. In addition, for the translocated partner regions on other chromosomes, it may be interesting to study whether they contain ReDFAS. If the analysis is performed with better and more detailed resolution even very small ReDFAS might become visible. The outcome of such investigations together with microscopic results and appropriate modeling might help to better understand why aberrations occur in certain chromatin regions. Such experiments and investigations will be the subject of future research.

Independently of the underlying mechanism, there seems to be some predisposition in the DNA sequence that influences the 3D chromatin organization under certain environmental conditions towards the induction of aberrations. Such changes would always indicate influences on other biological functions, for instance, the regulation of transcription of genes in these regions [52,53] or the cell fate in general [15].

## Figures and Tables

**Figure 6:**
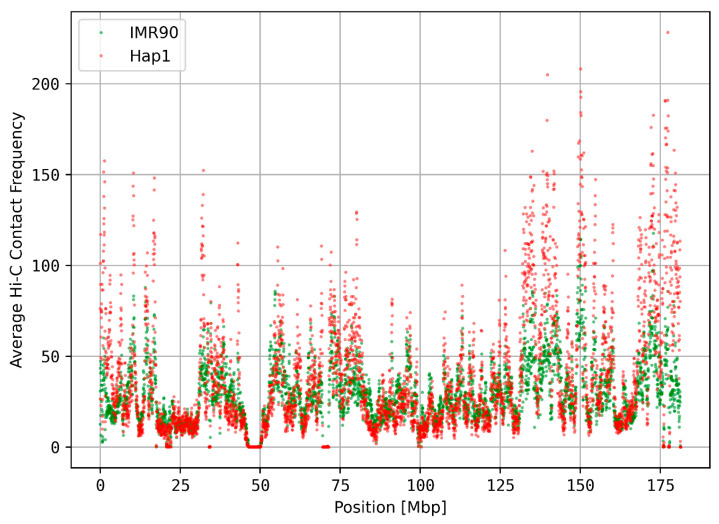
The Hi-C contact frequencies on chromosome 5 for different cell lines: The Pearson correlation between the two distributions is 0.787. (Red) The Hi-C frequencies in a cancerous chromosome 5 (cell line HAP1). (Green) The HiC frequencies in a healthy chromosome 5 (cell line IMR90).

**Table 1:** Definition of sets of DNA words of length *k* = 5 used for the classification of ReDFAS. N refers to “any nucleotide”, W (weak) is either A or T, S (strong) is either G or C.

Word Group	Corresponding 5-mers
A-rich	AAAAN, AAANA, AANAA, ANAAA, NAAAA
C-rich	CCCCN, CCCNC, CCNCC, CNCCC, NCCCC
G-rich	GGGGN, GGGNG, GGNGG, GNGGG, NGGGG
T-rich	TTTTN, TTTNT, TTNTT, TNTTT, NTTTT
AT-rich	WWWWW
GC-rich	SSSSS

**Table 2:** Coverage of ReDFAS, NPCs, CDS, Alu, L1 on human chromosomes and respective content of breakpoints (BP) located within ReDFAS, NPCs, CDS, Alu, and L1.

chr.	Cover ReDFAS	BP in ReDFAS	Cover NPC	BP in NPC	Cover CDS	BP in CDS	Cover Alu	BP in Alu	Cover L1	BP in L1
1	4%	9%	12%	6%	1%	38%	11%	3%	14%	1%
2	4%	8%	17%	8%	1%	39%	9%	4%	18%	1%
3	4%	12%	15%	7%	1%	37%	9%	3%	18%	1%
4	4%	14%	16%	7%	1%	35%	7%	3%	19%	1%
5	4%	11%	16%	7%	1%	36%	8%	3%	19%	1%
6	4%	11%	16%	8%	1%	35%	9%	4%	18%	1%
7	4%	7%	13%	7%	1%	38%	11%	3%	17%	1%
8	4%	9%	18%	7%	1%	39%	9%	2%	18%	0%
9	4%	10%	13%	4%	1%	40%	9%	3%	15%	1%
10	4%	7%	13%	7%	1%	40%	11%	3%	16%	1%
11	4%	9%	13%	10%	2%	37%	9%	3%	17%	1%
12	4%	7%	14%	6%	1%	38%	11%	3%	16%	0%
13	4%	12%	14%	8%	1%	38%	7%	2%	15%	1%
14	4%	9%	15%	11%	1%	41%	9%	4%	14%	0%
15	3%	6%	13%	8%	1%	38%	10%	4%	14%	0%
16	4%	6%	11%	4%	2%	42%	14%	3%	11%	0%
17	2%	0%	11%	6%	2%	40%	18%	2%	10%	0%
18	2%	0%	12%	4%	1%	37%	8%	2%	16%	1%
19	3%	0%	9%	5%	4%	40%	25%	2%	10%	0%
20	2%	1%	12%	6%	1%	38%	12%	5%	14%	1%
21	2%	1%	15%	4%	1%	32%	7%	1%	13%	0%
22	2%	0%	10%	7%	2%	36%	22%	2%	8%	0%
X	3%	12%	8%	5%	1%	35%	8%	3%	29%	1%
Y	4%	33%	3%	13%	0%	33%	4%	10%	11%	0%

**Table 3:** Significance level of enrichments of BPs in ReDFAS and genomic elements (PC, NPC, CDS, Alu, and L1) as well as of genomic elements in ReDFAS.

Enrichment Tested	Difference [*σ*]
BP in ReDFAS	18
BP in PC	84
BP in NPC	−15
BP in CDS	261
BP in Alu	−17
BP in L1	−32
PC in ReDFAS	13
NPC in ReDFAS	5.1
CDS in ReDFAS	76
Alu in ReDFAS	−2.2
L1 in ReDFAS	−23

**Table 4:** Correlation of spectral deviations and transposons.

Correlated Map	Empirical Correlation	Reference Correlation
ALU	0.17 ± 0.11	0.00 ± 0.09
L1	−0.11 ± 0.11	0.01 ± 0.09

## Data Availability

All codes and scripts (including visualization) used for this article, as well as a manual, are available online at “http://www.kip.uni-heidelberg.de/biophysik/software (accessed on 27 September 2021)” or from an associated GitHub repository (https://github.com/Sievers-A/Oligo. Accessed on 27 September 2021).

## Supplementary Materials

The following supporting information can be downloaded at: https://www.mdpi.com/article/10.3390/genes15101247/s1, Figure S1: Comparison of Approaches for Deviations, Figure S2–S23: Average *k*-mer Spectrum Deviations for chromosomes 1–4, 6–8, 10–Y; Figure S24–S46: The Spectral Deviations of Segments for chromosomes 1–4, 6–8, 10–Y; Figure S47–S69: PCA Results *k* = 5 on chromosomes 1–4, 6–8, 10–Y; Figure S70–S92: Relation between ReDFAS and G+C Content for chromosomes 1–4, 6–8, 10–Y; Figure S93–S115: The Hi-C Contact Frequencies for chromosomes 1–4, 6–8, 10–Y; Figure S116: The Spectral Deviations of Segments on Chromosome 5 for all word sets.
