# Supplementary material for: Peculiar k-mer Spectra Are Correlated with 3D Contact Frequencies and Breakpoint Regions in the Human Genome
Source: Genes (Basel). 2024 Sep 25;15(10):1247. doi: 10.3390/genes15101247 (PMC11506876; doi:10.3390/genes15101247)
Supplement: Supplementary file 1 [file genes-15-01247-s001.zip › genes-3147578-supplementary.pdf]

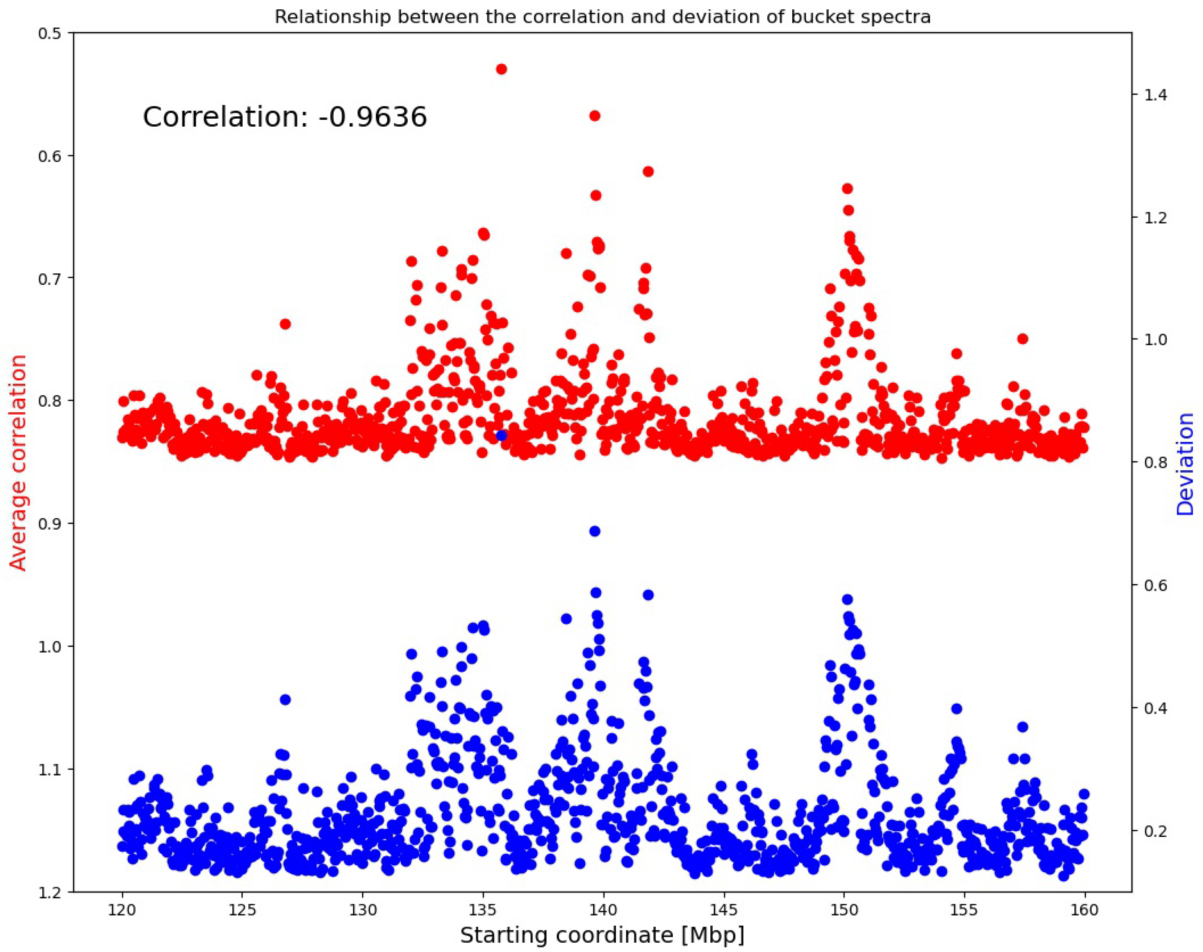

Figure S1: Comparison of Approaches for Deviations: Correlation between average correlation in blue (right axis) and the
deviation of the section spectra in red (left axis), the memory intensive correlation of thousands of sections can be replaced by the spectrum deviation with a very high confidence

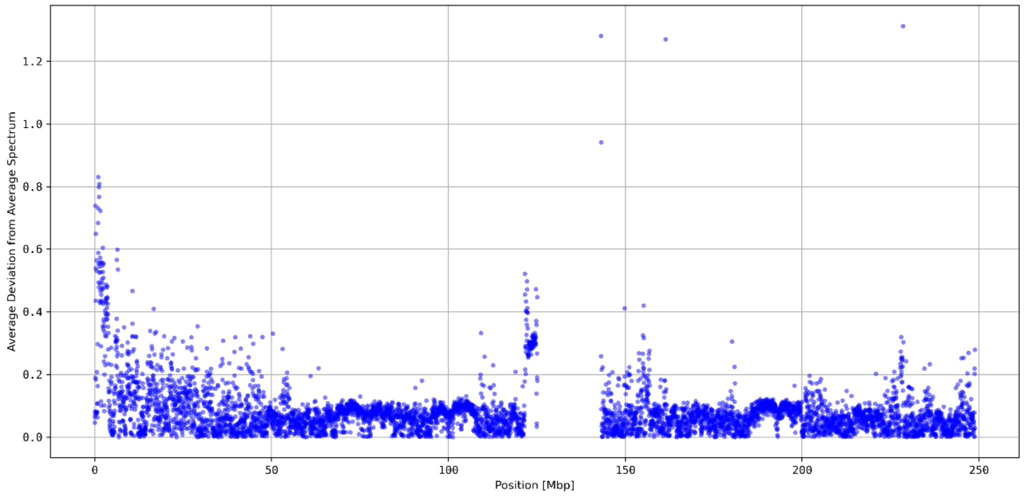

Figure S2: Average k-mer spectrum deviation of each 40 kbp segment (for k = 5) on chromosome 1

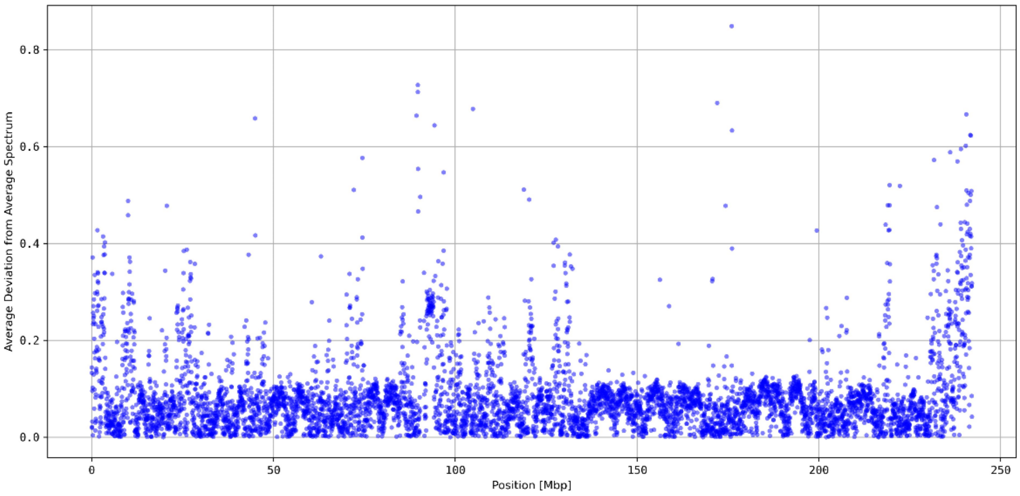

Figure S3: Average k-mer spectrum deviation of each 40 kbp segment (for k = 5) on chromosome 2

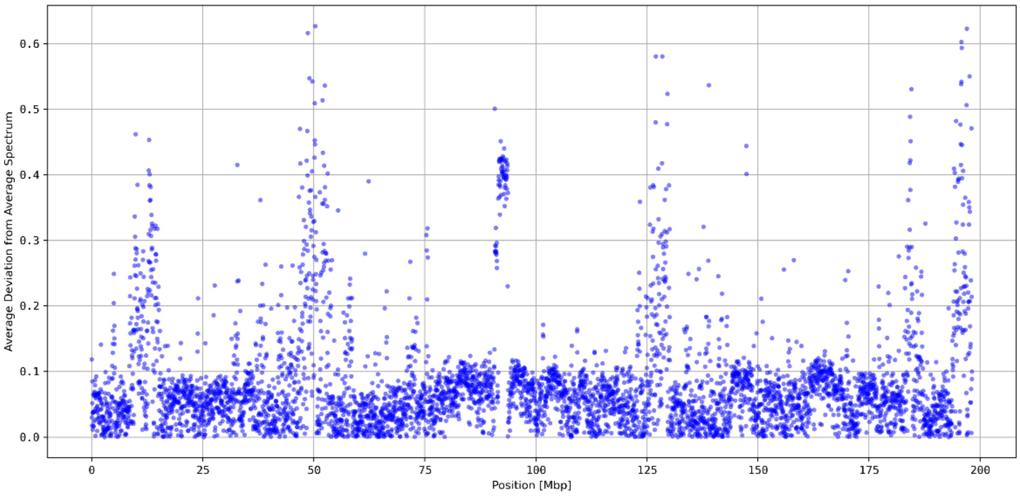

Figure S4: Average k-mer spectrum deviation of each 40 kbp segment (for k = 5) on chromosome 3

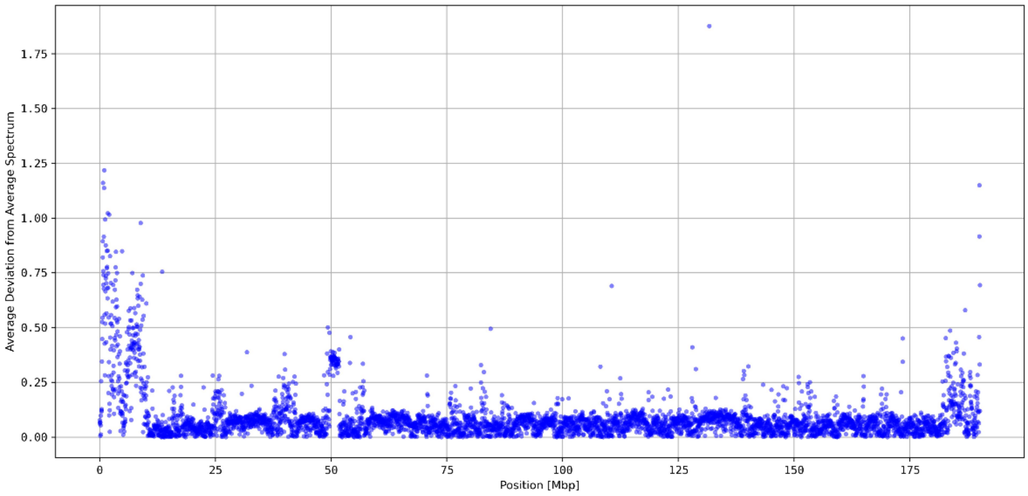

Figure S5: Average k-mer spectrum deviation of each 40 kbp segment (for k = 5) on chromosome 4

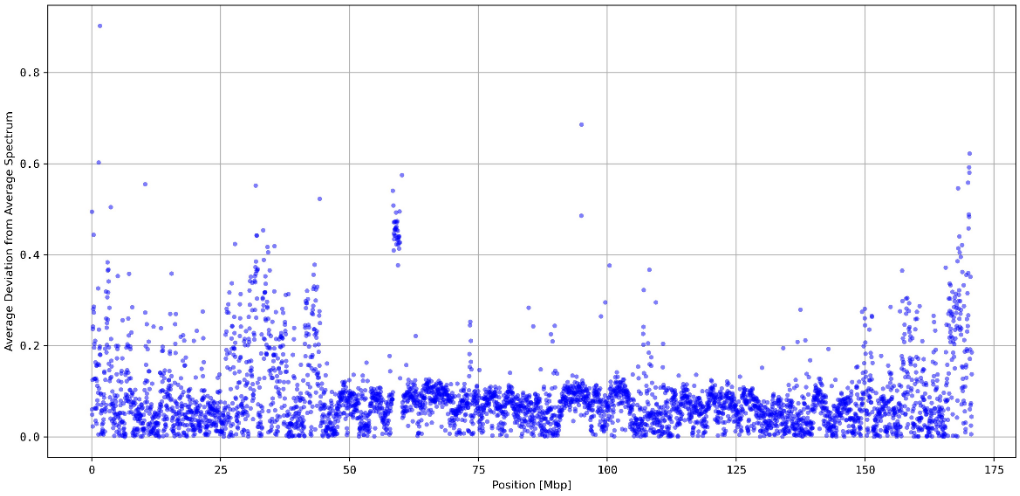

Figure S6: Average k-mer spectrum deviation of each 40 kbp segment (for k = 5) on chromosome 6

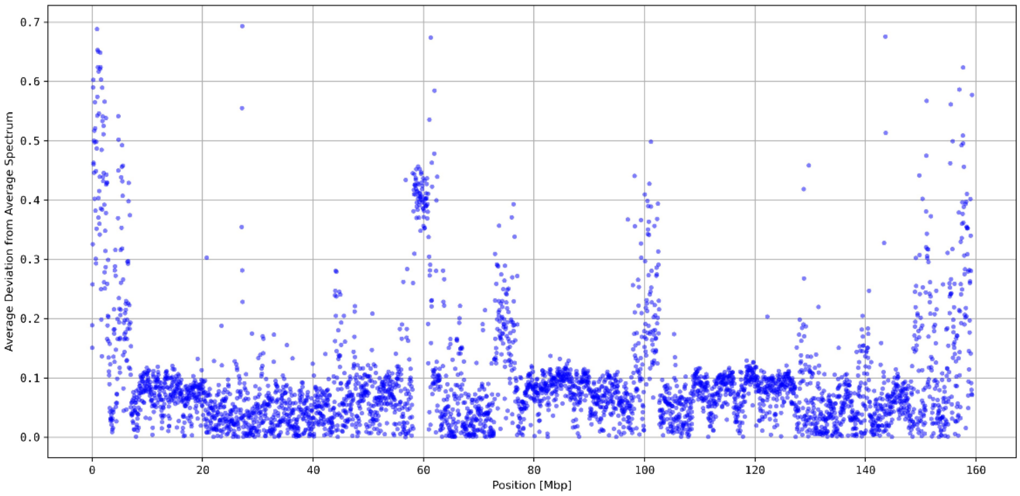

Figure S7: Average k-mer spectrum deviation of each 40 kbp segment (for k = 5) on chromosome 7

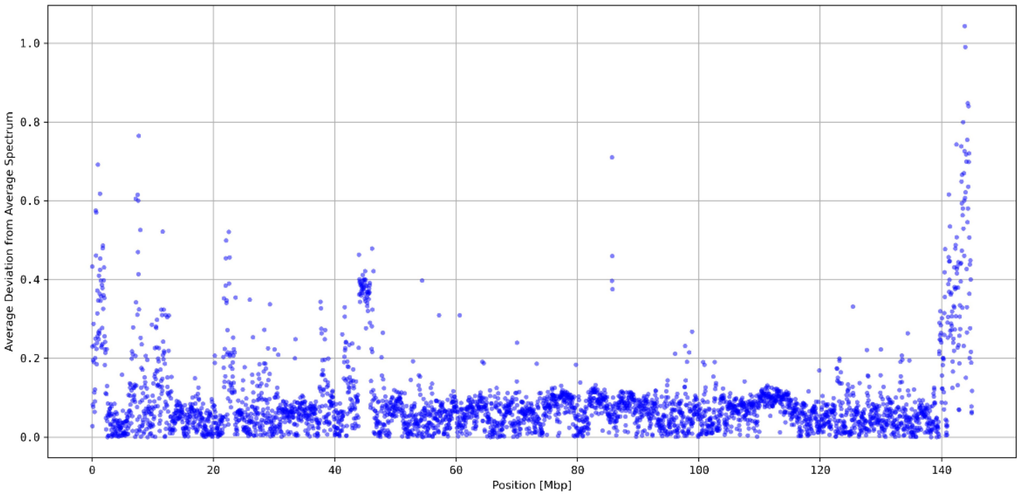

Figure S8: Average k-mer spectrum deviation of each 40 kbp segment (for k = 5) on chromosome 8

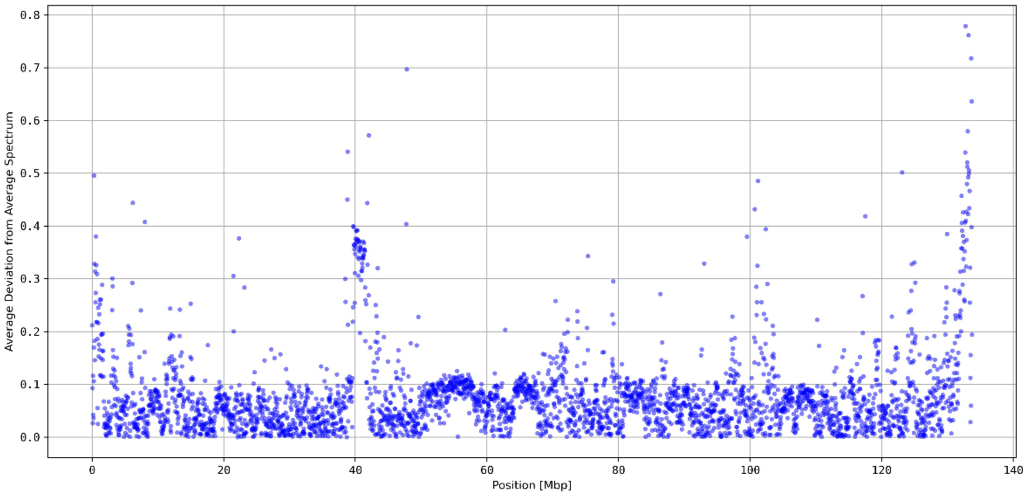

Figure S9: Average k-mer spectrum deviation of each 40 kbp segment (for k = 5) on chromosome 10

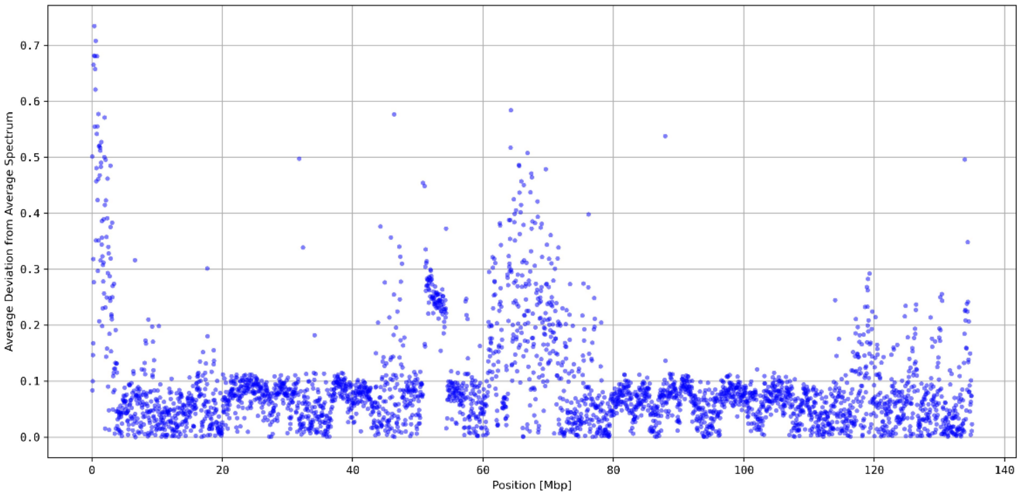

Figure S10: Average k-mer spectrum deviation of each 40 kbp segment (for k = 5) on chromosome 11

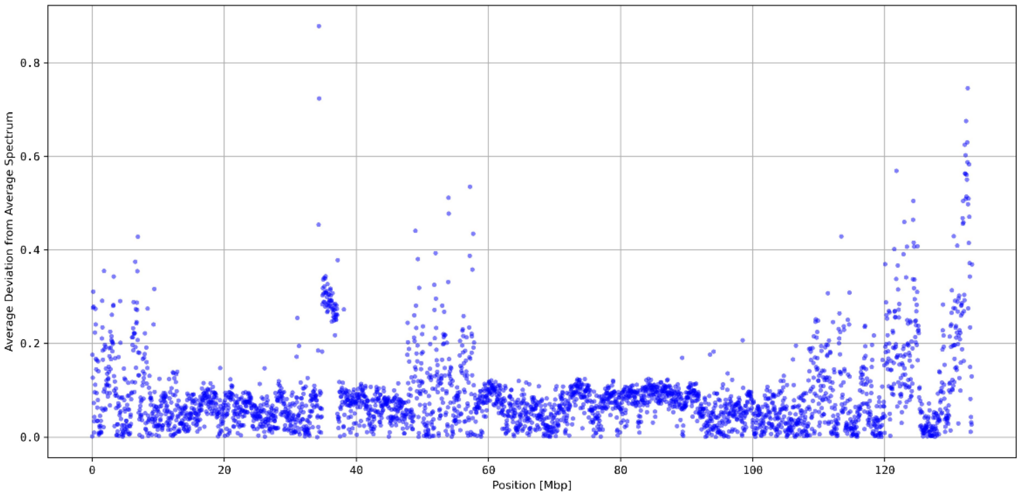

Figure S11: Average k-mer spectrum deviation of each 40 kbp segment (for k = 5) on chromosome 12

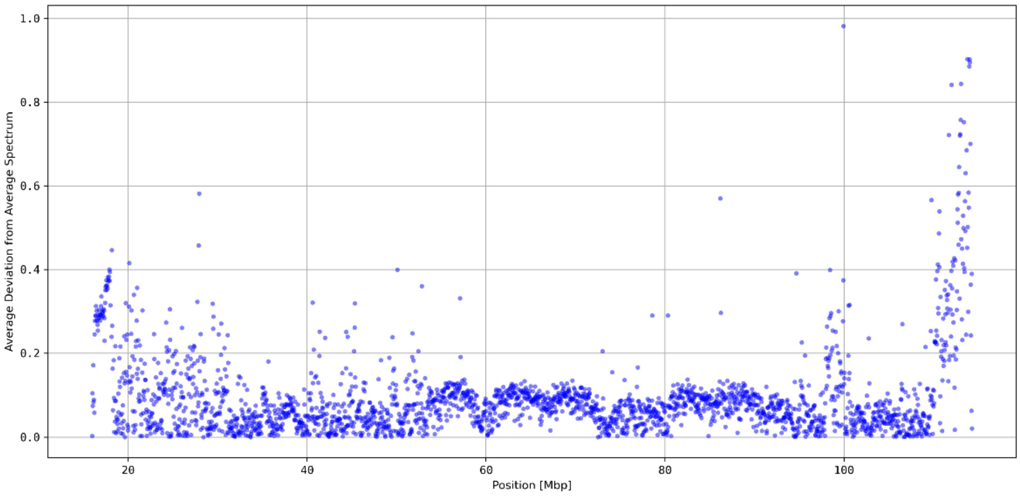

Figure S12: Average k-mer spectrum deviation of each 40 kbp segment (for k = 5) on chromosome 13

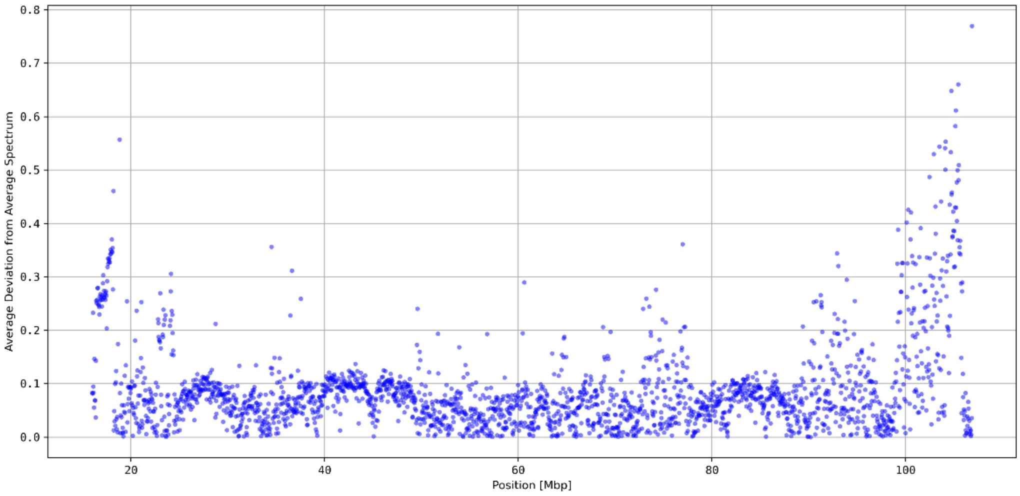

Figure S13: Average k-mer spectrum deviation of each 40 kbp segment (for k = 5) on chromosome 14

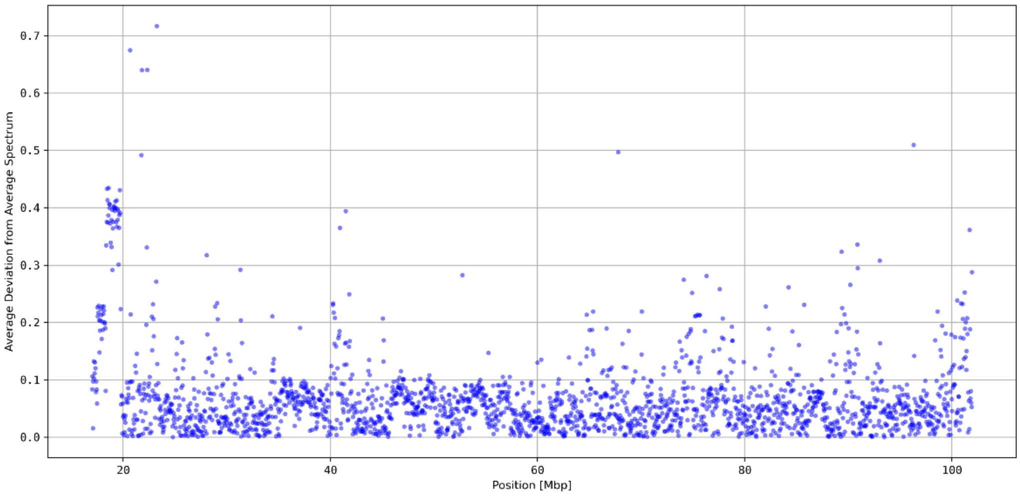

Figure S14: Average k-mer spectrum deviation of each 40 kbp segment (for k = 5) on chromosome 15

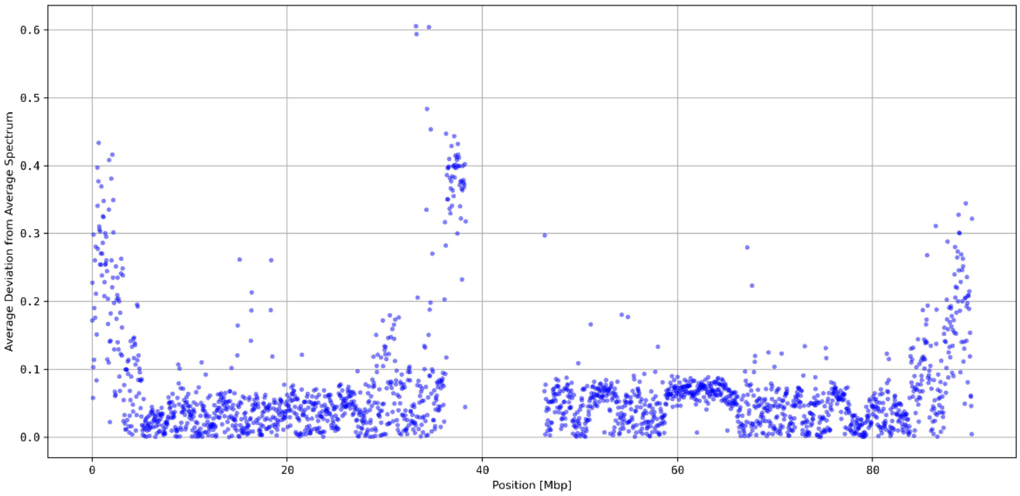

Figure S15: Average k-mer spectrum deviation of each 40 kbp segment (for k = 5) on chromosome 16

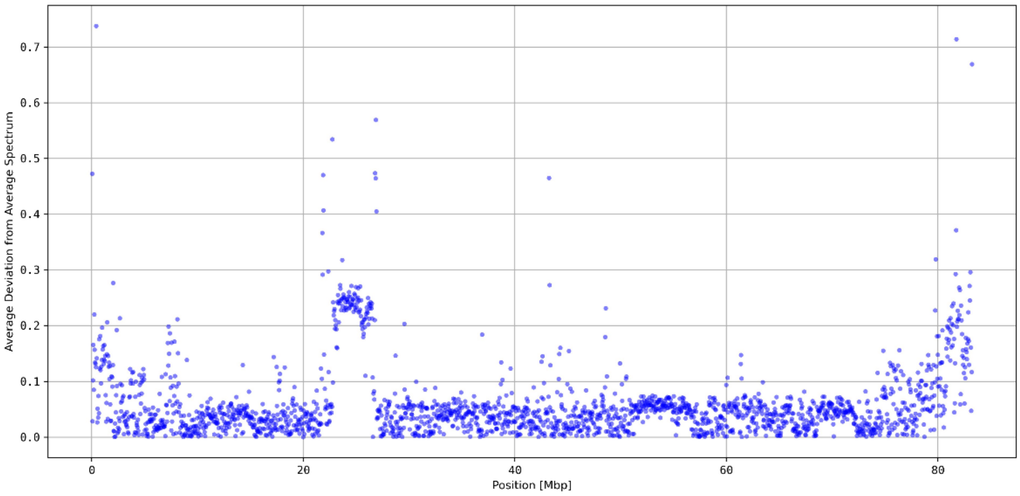

Figure S16: Average k-mer spectrum deviation of each 40 kbp segment (for k = 5) on chromosome 17

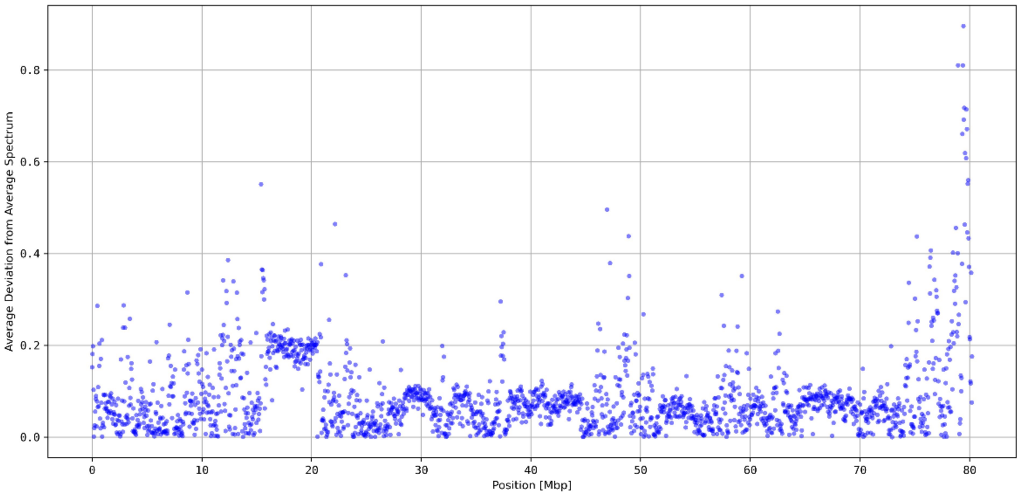

Figure S17: Average k-mer spectrum deviation of each 40 kbp segment (for k = 5) on chromosome 18

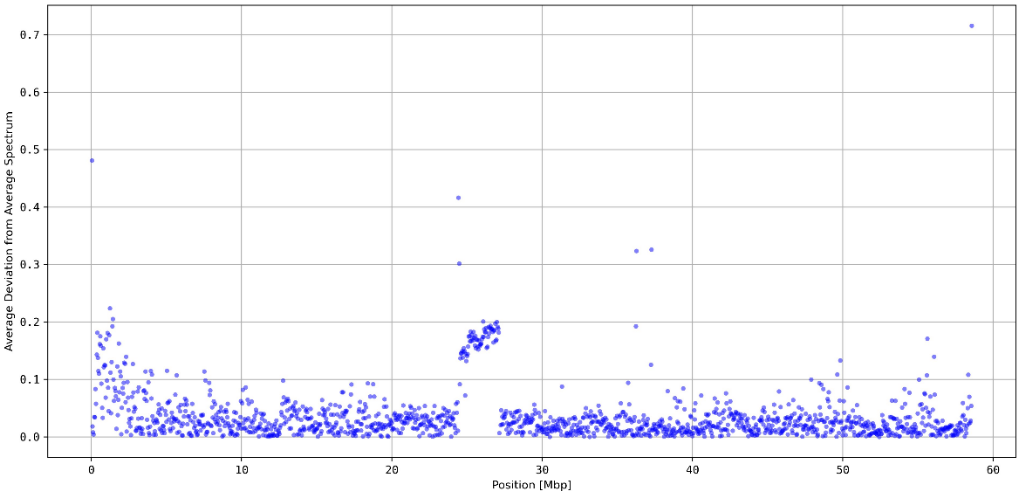

Figure S18: Average k-mer spectrum deviation of each 40 kbp segment (for k = 5) on chromosome 19

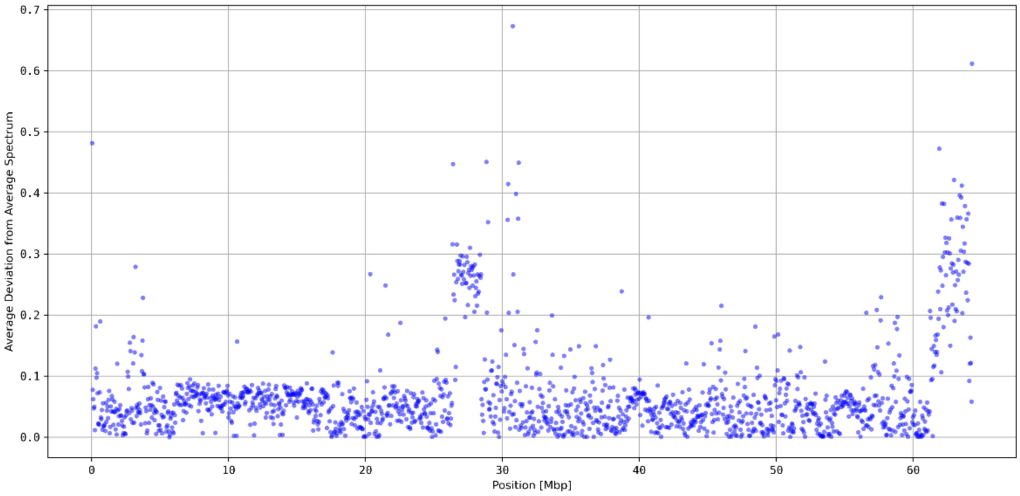

Figure S19: Average k-mer spectrum deviation of each 40 kbp segment (for k = 5) on chromosome 20

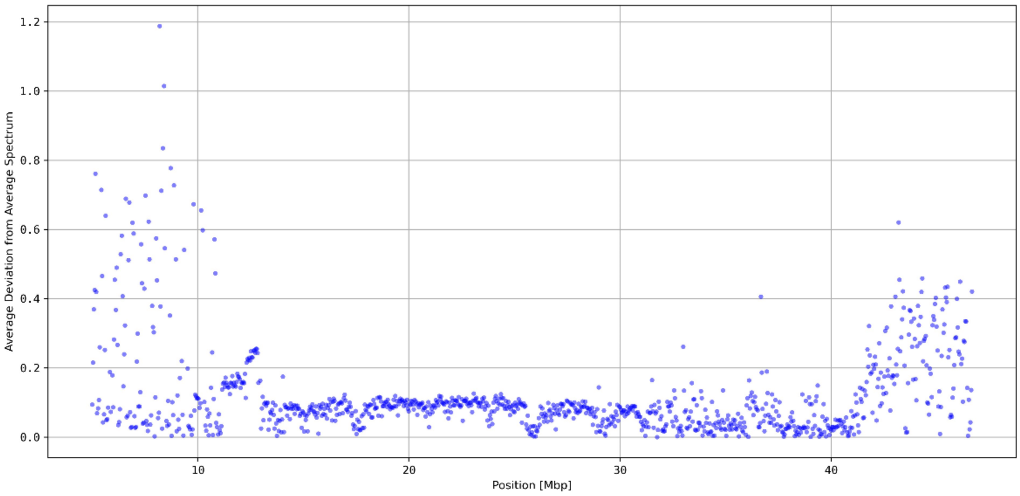

Figure S20: Average k-mer spectrum deviation of each 40 kbp segment (for k = 5) on chromosome 21

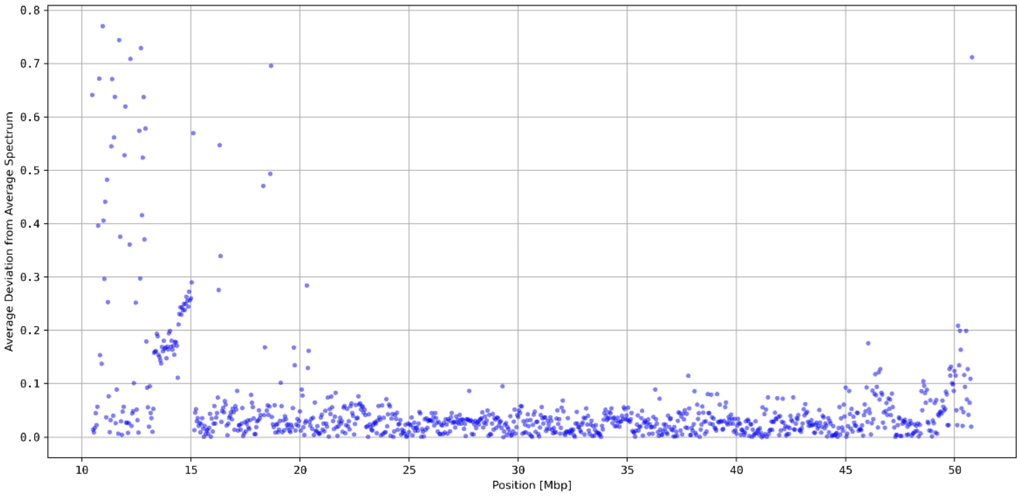

Figure S21: Average k-mer spectrum deviation of each 40 kbp segment (for k = 5) on chromosome 22

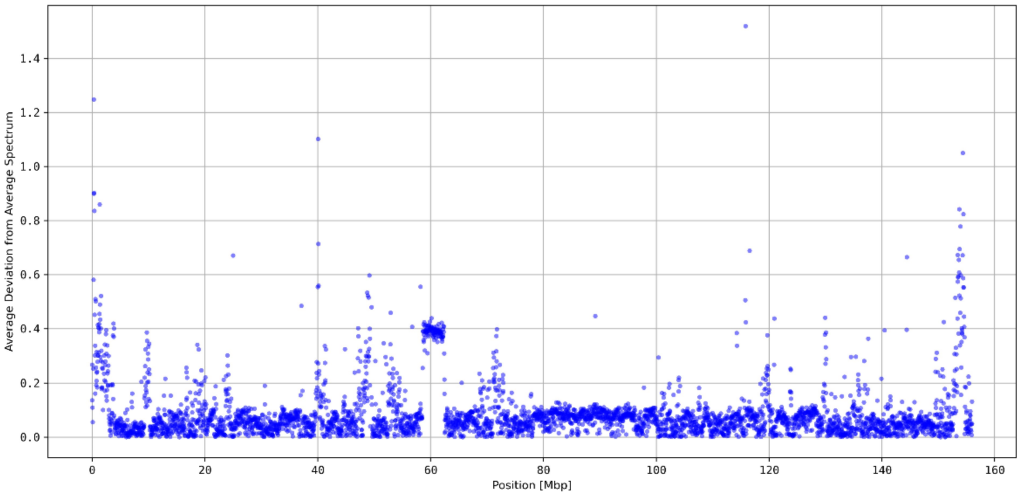

Figure S22: Average k-mer spectrum deviation of each 40 kbp segment (for k = 5) on chromosome X

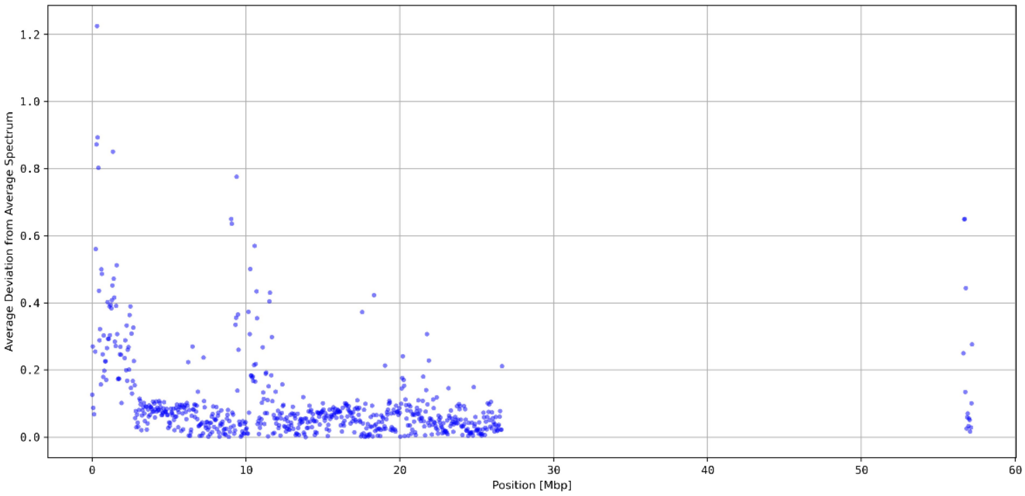

Figure S23: Average k-mer spectrum deviation of each 40 kbp segment (for k = 5) on chromosome Y

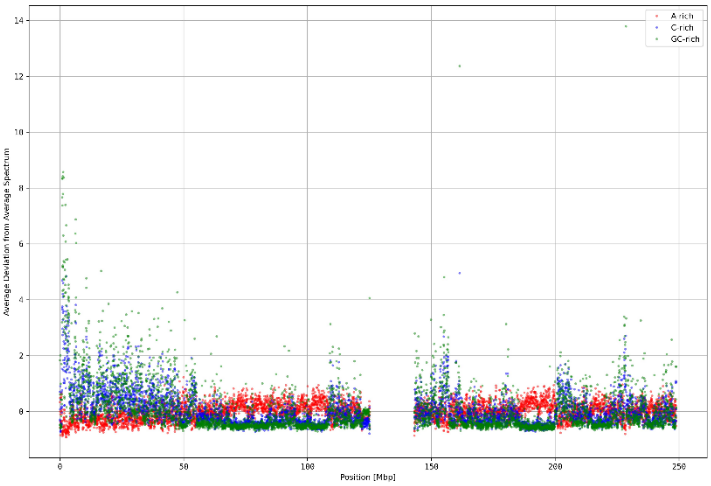

Figure S24: The spectral deviations of segments on chromosome 1 for a selection of representative word sets (see Table 2). ● = A-rich; ● = C-rich; ● = GC-rich

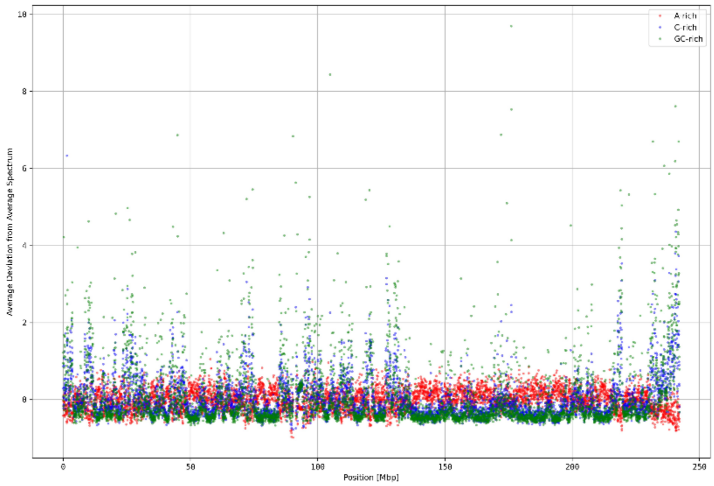

Figure S25: The spectral deviations of segments on chromosome 2 for a selection of representative word sets (see Table 2). ● = A-rich; ● = C-rich; ● = GC-rich

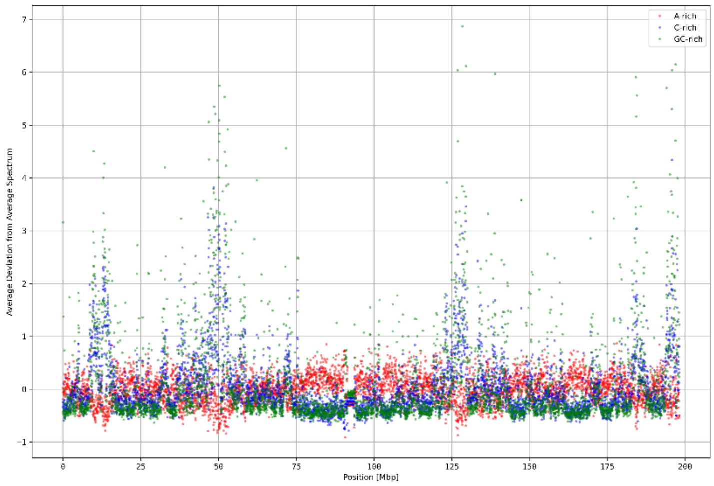

Figure S26: The spectral deviations of segments on chromosome 3 for a selection of representative word sets (see Table 2). ● = A-rich; ● = C-rich; ● = GC-rich

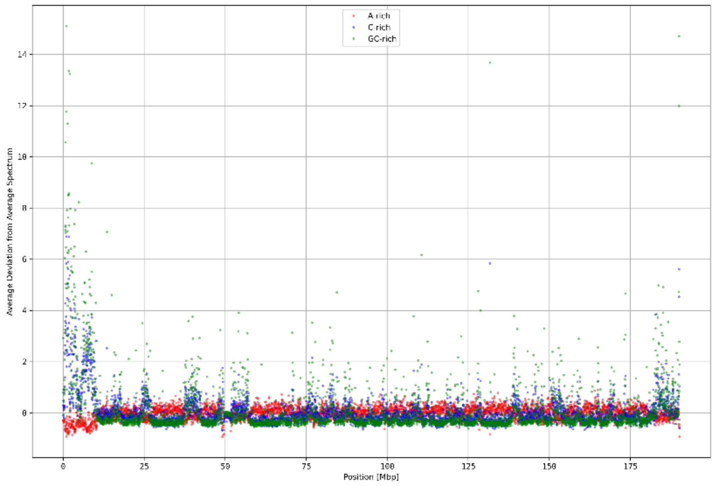

Figure S27: The spectral deviations of segments on chromosome 4 for a selection of representative word sets (see Table 2). ● = A-rich; ● = C-rich; ● = GC-rich

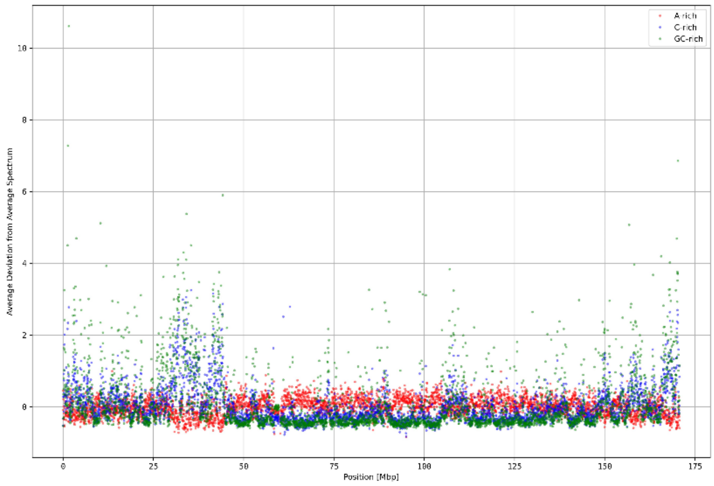

Figure S28: The spectral deviations of segments on chromosome 6 for a selection of representative word sets (see Table 2). ● = A-rich; ● = C-rich; ● = GC-rich

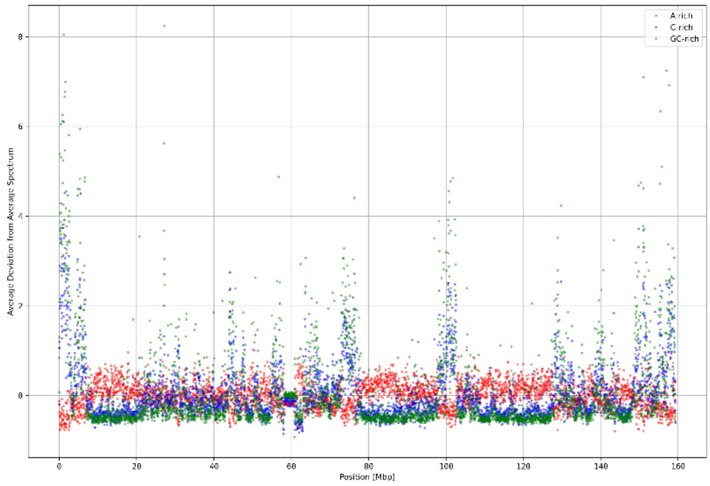

Figure S29: The spectral deviations of segments on chromosome 7 for a selection of representative word sets (see Table 2). ● = A-rich; ● = C-rich; ● = GC-rich

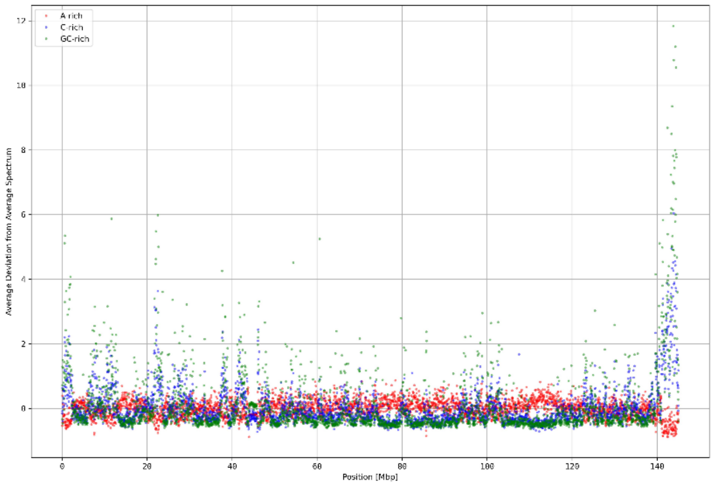

Figure S30: The spectral deviations of segments on chromosome 8 for a selection of representative word sets (see Table 2). ● = A-rich; ● = C-rich; ● = GC-rich

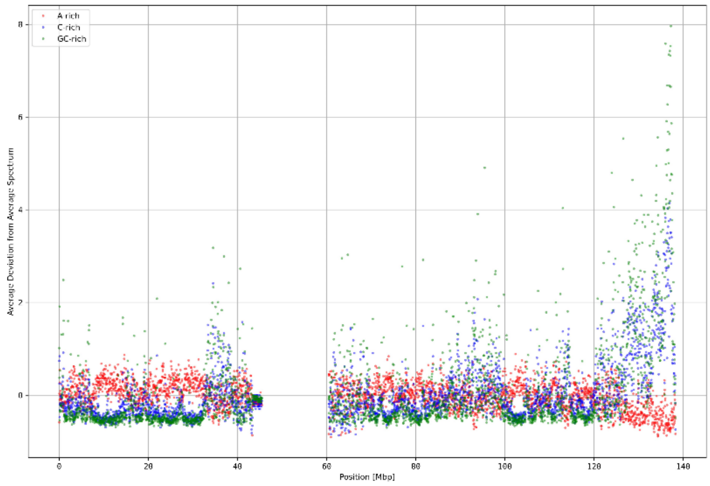

Figure S31: The spectral deviations of segments on chromosome 9 for a selection of representative word sets (see Table 2). ● = A-rich; ● = C-rich; ● = GC-rich

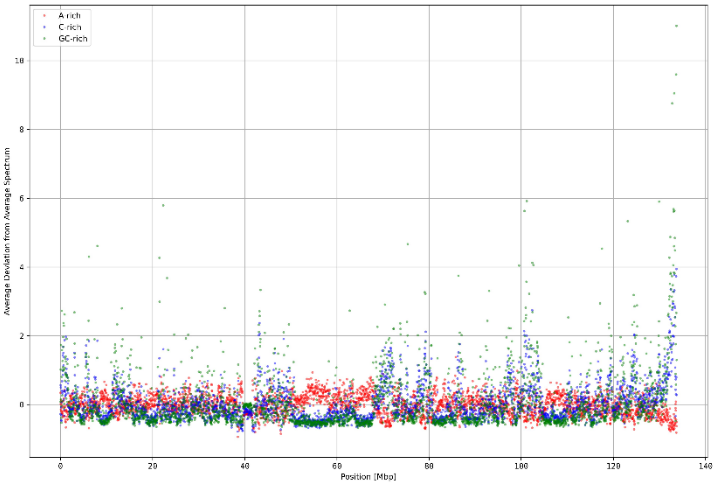

Figure S32: The spectral deviations of segments on chromosome 10 for a selection of representative word sets (see Table 2). ● = A-rich; ● = C-rich; ● = GC-rich

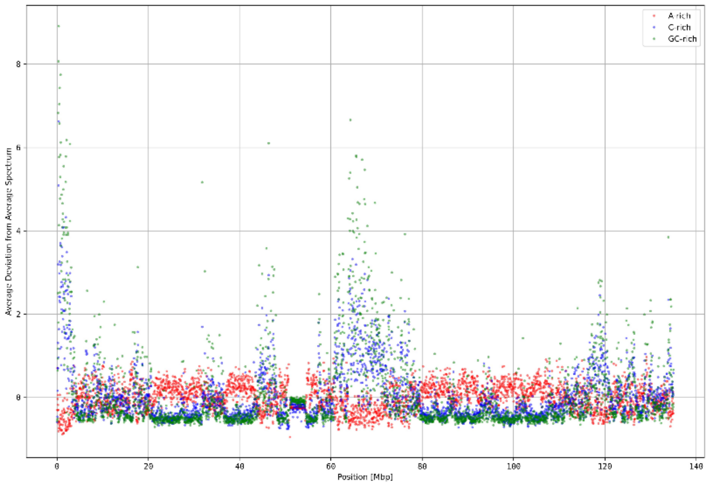

Figure S33: The spectral deviations of segments on chromosome 11 for a selection of representative word sets (see Table 2). ● = A-rich; ● = C-rich; ● = GC-rich

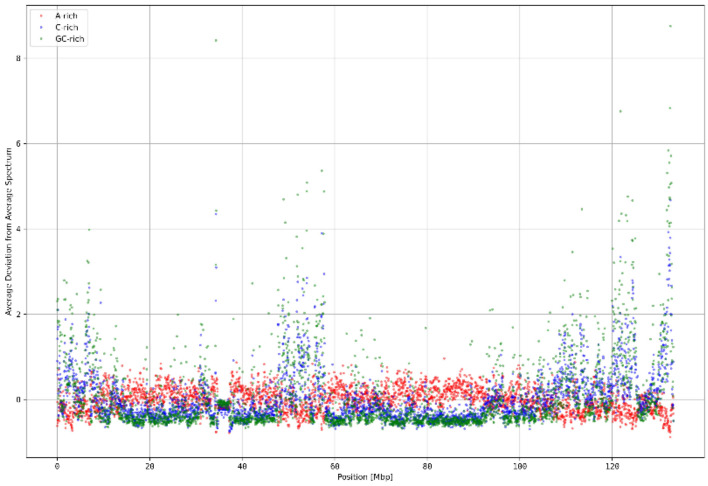

Figure S34: The spectral deviations of segments on chromosome 12 for a selection of representative word sets (see Table 2). ● = A-rich; ● = C-rich; ● = GC-rich

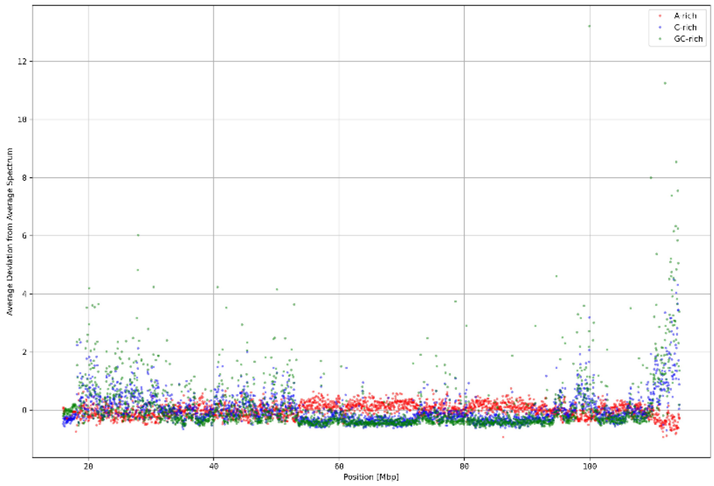

Figure S35: The spectral deviations of segments on chromosome 13 for a selection of representative word sets (see Table 2). ● = A-rich; ● = C-rich; ● = GC-rich

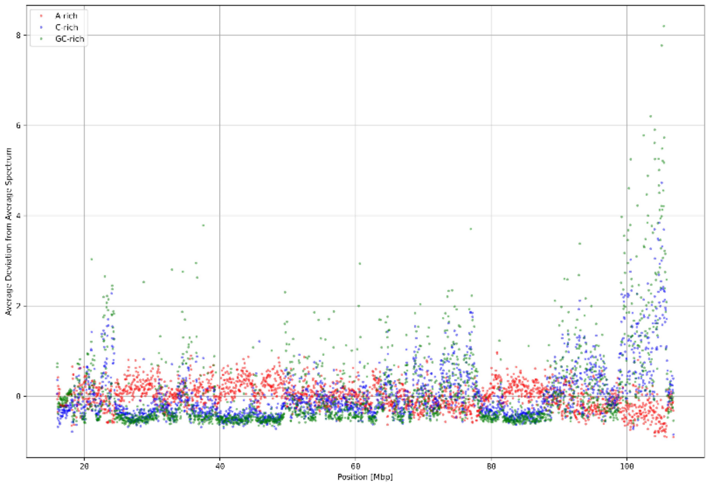

Figure S36: The spectral deviations of segments on chromosome 14 for a selection of representative word sets (see Table 2). ● = A-rich; ● = C-rich; ● = GC-rich

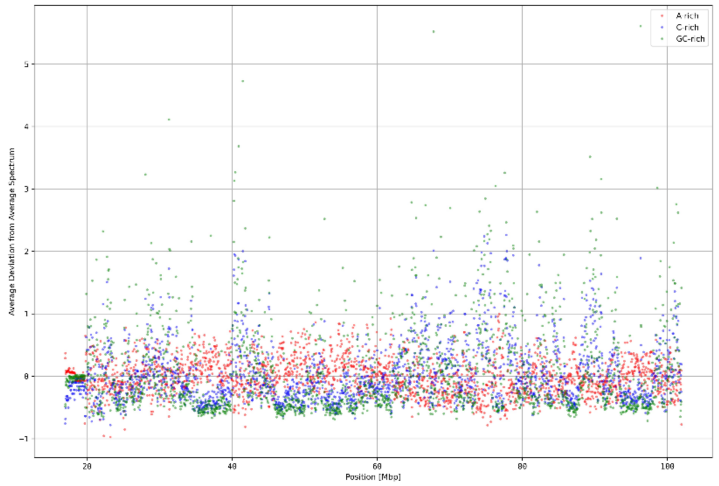

Figure S37: The spectral deviations of segments on chromosome 15 for a selection of representative word sets (see Table 2). ● = A-rich; ● = C-rich; ● = GC-rich

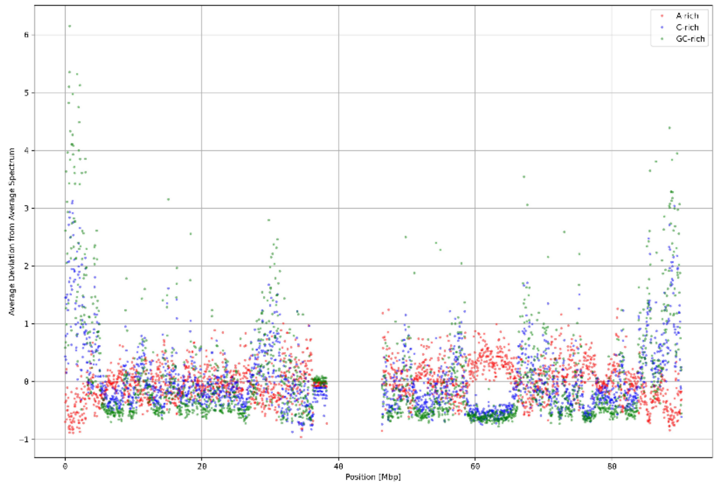

Figure S38: The spectral deviations of segments on chromosome 16 for a selection of representative word sets (see Table 2). ● = A-rich; ● = C-rich; ● = GC-rich

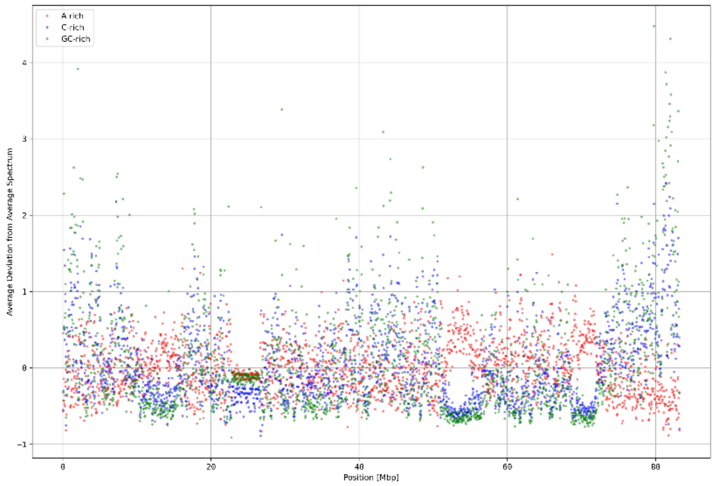

Figure S39: The spectral deviations of segments on chromosome 17 for a selection of representative word sets (see Table 2). 🔴 = A-rich; 🔵 = C-rich; 🟢 = GC-rich

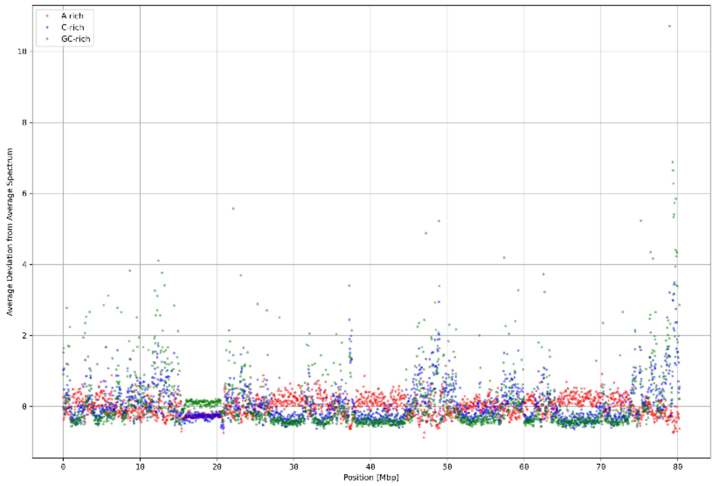

Figure S40: The spectral deviations of segments on chromosome 18 for a selection of representative word sets (see Table 2). 🔴 = A-rich; 🔵 = C-rich; 🟢 = GC-rich

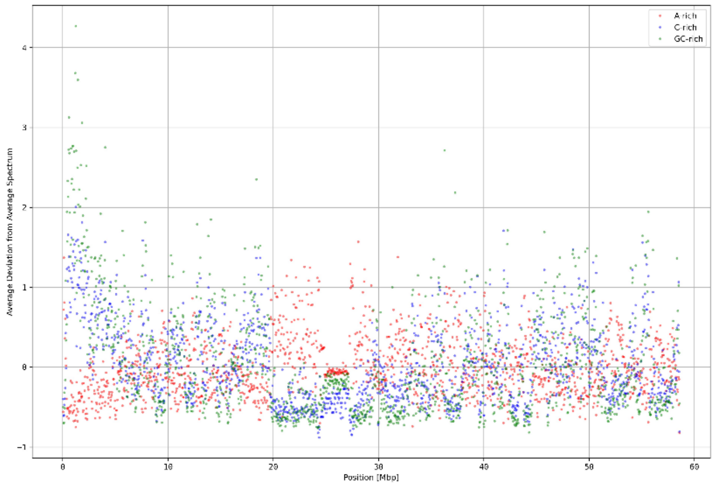

Figure S41: The spectral deviations of segments on chromosome 19 for a selection of representative word sets (see Table 2). ● = A-rich; ● = C-rich; ● = GC-rich

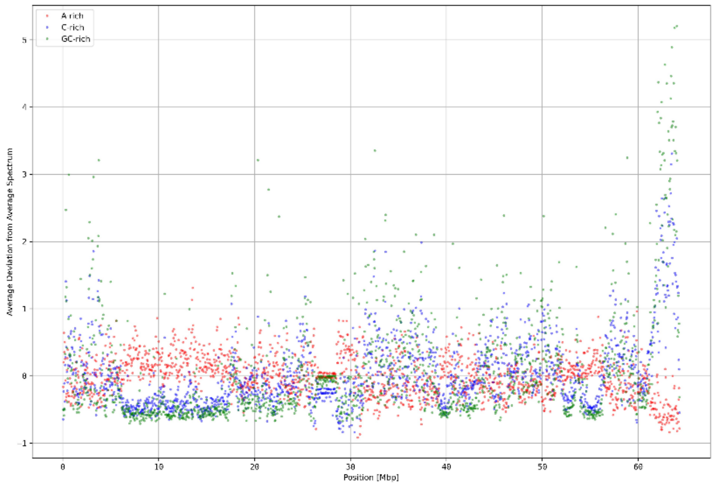

Figure S42: The spectral deviations of segments on chromosome 20 for a selection of representative word sets (see Table 2). ● = A-rich; ● = C-rich; ● = GC-rich

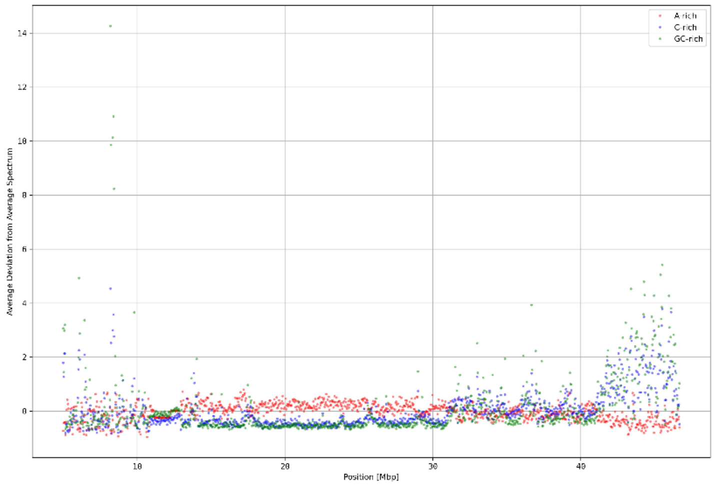

Figure S43: The spectral deviations of segments on chromosome 21 for a selection of representative word sets (see Table 2). ● = A-rich; ● = C-rich; ● = GC-rich

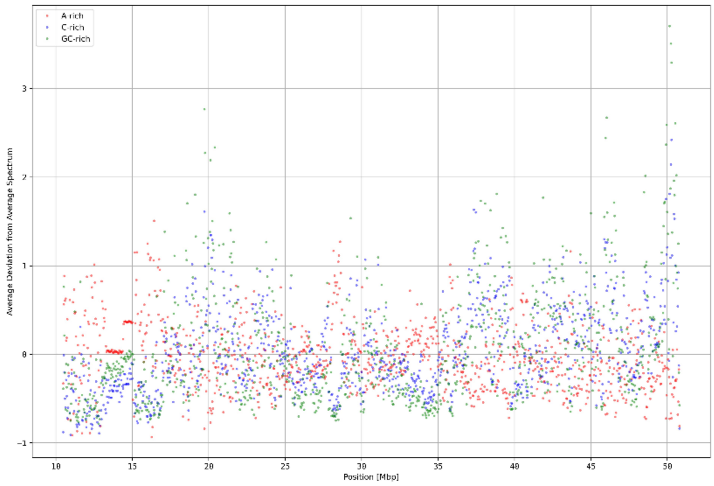

Figure S44: The spectral deviations of segments on chromosome 22 for a selection of representative word sets (see Table 2). ● = A-rich; ● = C-rich; ● = GC-rich

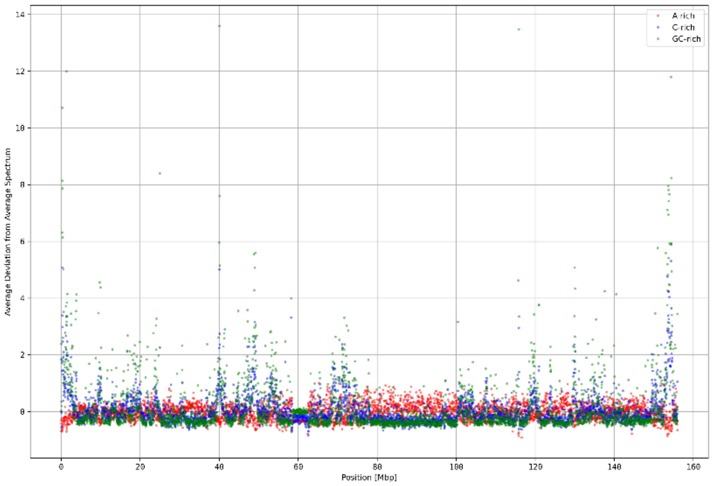

Figure S45: The spectral deviations of segments on chromosome X for a selection of representative word sets (see Table 2). ● = A-rich; ● = C-rich; ● = GC-rich

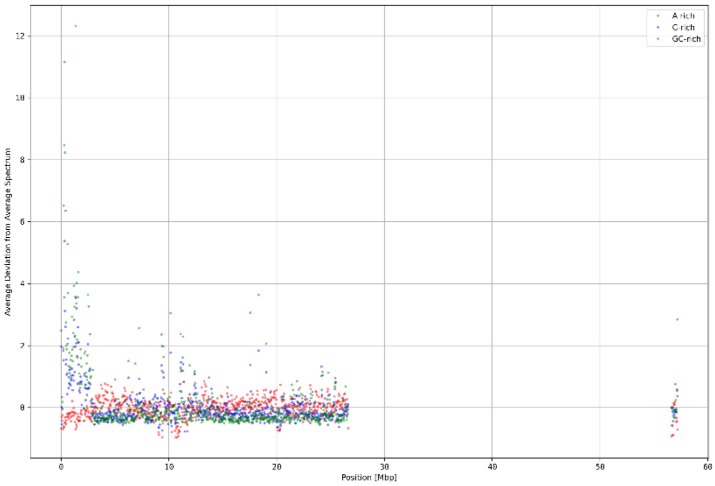

Figure S46: The spectral deviations of segments on chromosome Y for a selection of representative word sets (see Table 2). ● = A-rich; ● = C-rich; ● = GC-rich

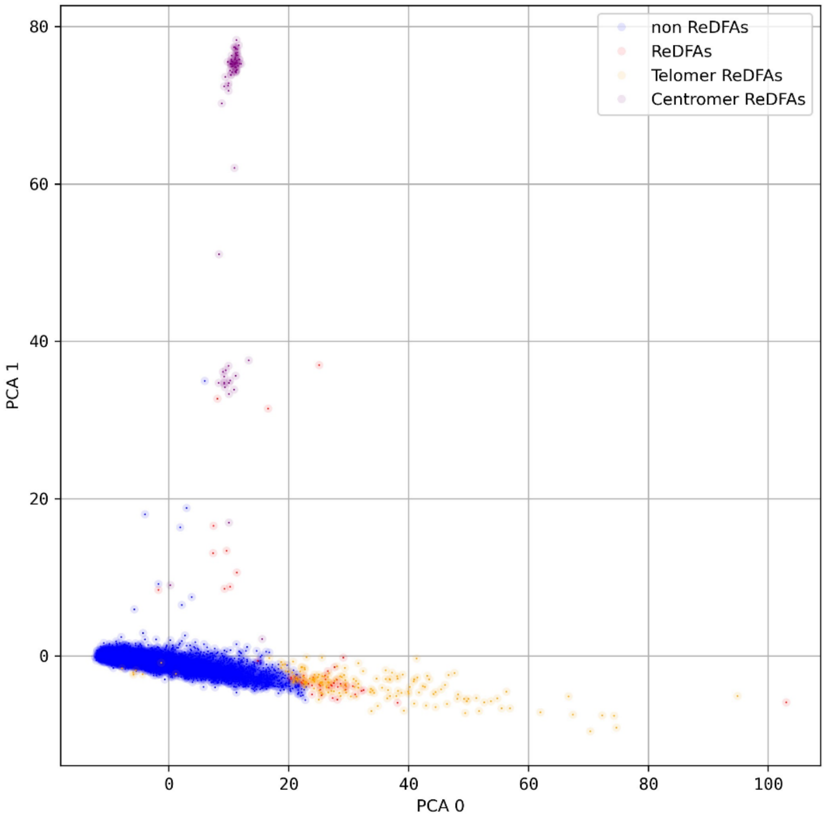

Figure S47. PCA results k = 5 on chromosome 1: PCA was performed on k = 5 spectra of 40 kbp segments on the chromosome. Different classes of regions were labeled with different colors. red: intermediate ReDFAS without special label; yellow: subtelomeric ReDFAS; purple: centromeric ReDFAS; blue: segments not labeled as ReDFAS.

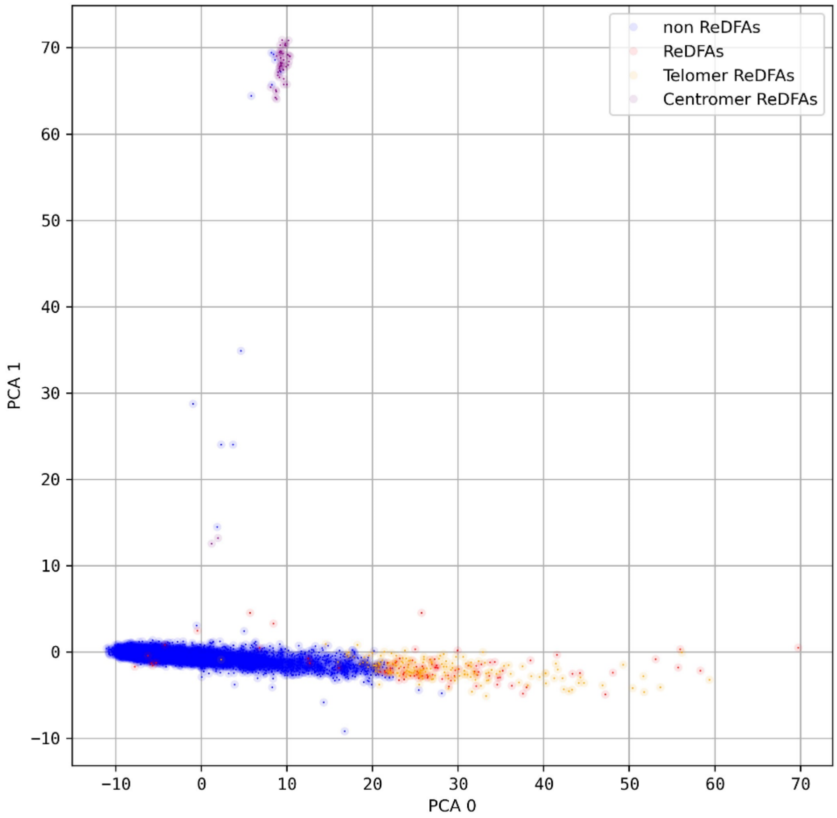

Figure S48. PCA results k = 5 on chromosome 2: PCA was performed on k = 5 spectra of 40 kbp segments on the chromosome. Different classes of regions were labeled with different colors. red: intermediate ReDFAS without special label; yellow: subtelomeric ReDFAS; purple: centromeric ReDFAS; blue: segments not labeled as ReDFAS.

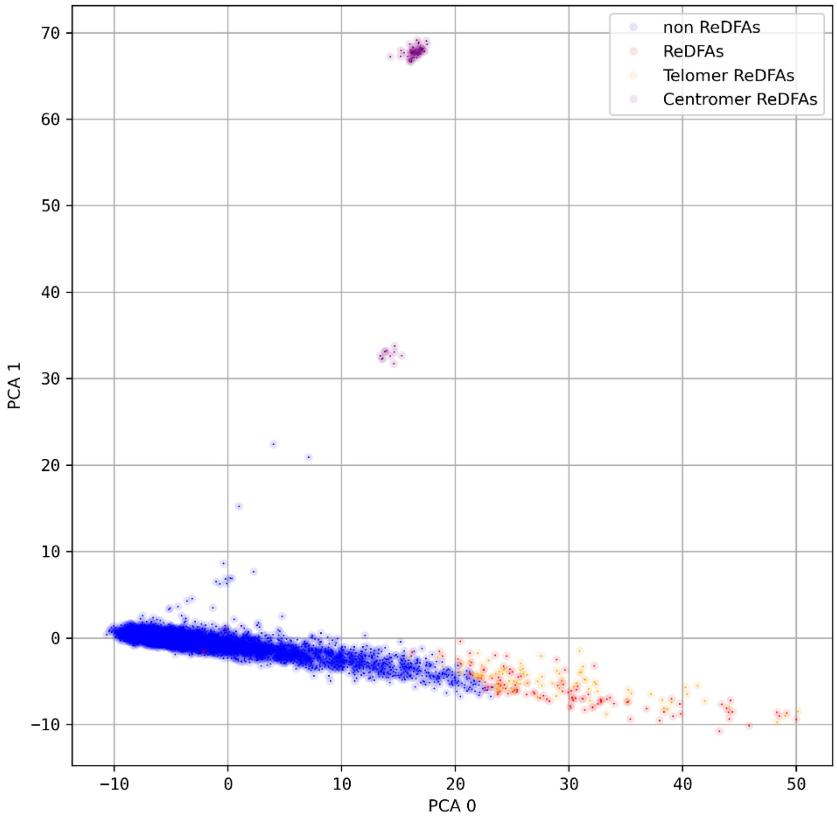

Figure S49. PCA results k = 5 on chromosome 3: PCA was performed on k = 5 spectra of 40 kbp segments on the chromosome. Different classes of regions were labeled with different colors. red: intermediate ReDFAS without special label; yellow: subtelomeric ReDFAS; purple: centromeric ReDFAS; blue: segments not labeled as ReDFAS.

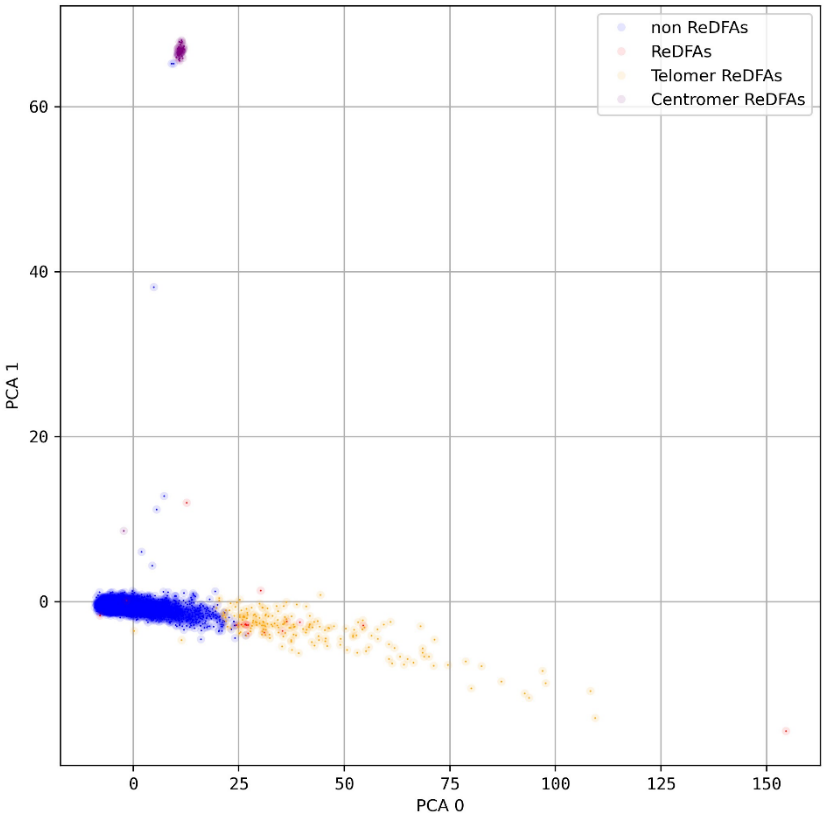

Figure S50. PCA results k = 5 on chromosome 4: PCA was performed on k = 5 spectra of 40 kbp segments on the chromosome. Different classes of regions were labeled with different colors. red: intermediate ReDFAS without special label; yellow: subtelomeric ReDFAS; purple: centromeric ReDFAS; blue: segments not labeled as ReDFAS.

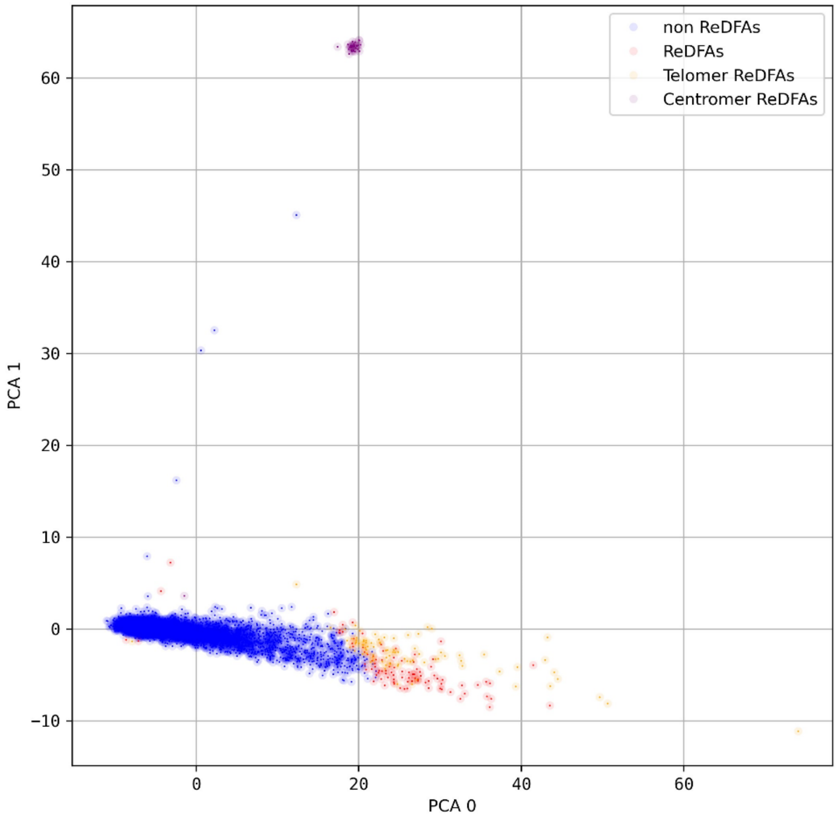

Figure S51. PCA results k = 5 on chromosome 6: PCA was performed on k = 5 spectra of 40 kbp segments on the chromosome. Different classes of regions were labeled with different colors. red: intermediate ReDFAS without special label; yellow: subtelomeric ReDFAS; purple: centromeric ReDFAS; blue: segments not labeled as ReDFAS.

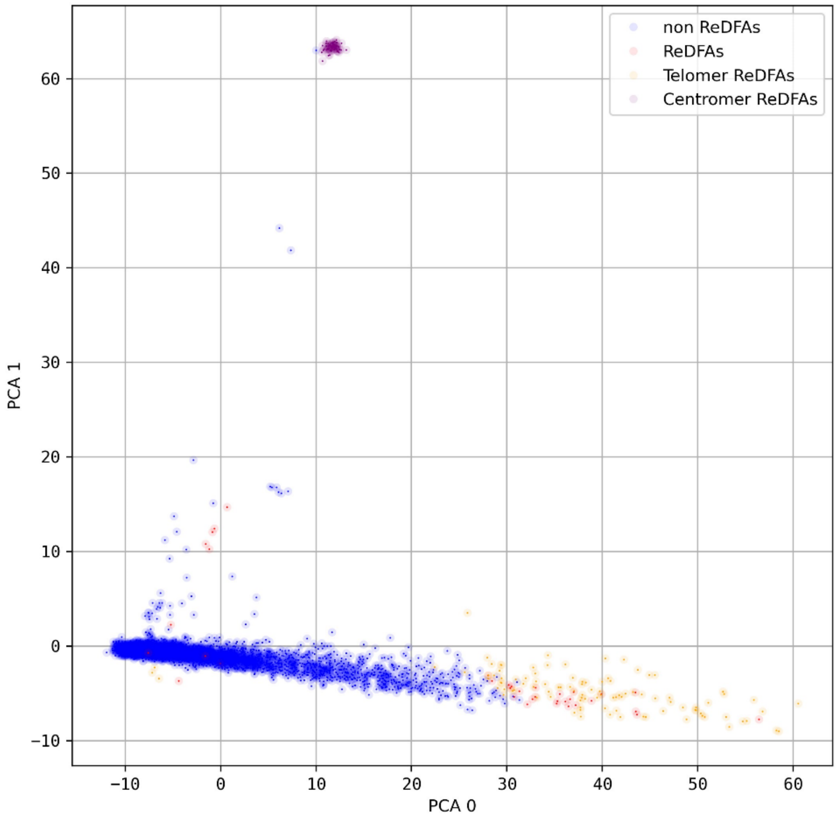

Figure S52. PCA results k = 5 on chromosome 7: PCA was performed on k = 5 spectra of 40 kbp segments on the chromosome. Different classes of regions were labeled with different colors. red: intermediate ReDFAS without special label; yellow: subtelomeric ReDFAS; purple: centromeric ReDFAS; blue: segments not labeled as ReDFAS.

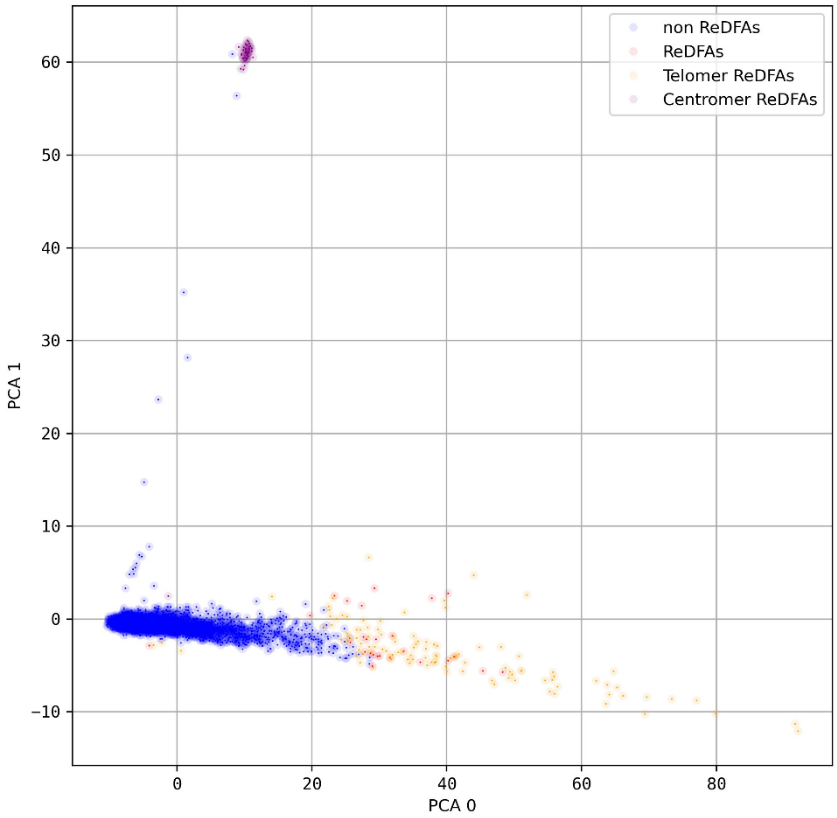

Figure S53. PCA results k = 5 on chromosome 8: PCA was performed on k = 5 spectra of 40 kbp segments on the chromosome. Different classes of regions were labeled with different colors. red: intermediate ReDFAS without special label; yellow: subtelomeric ReDFAS; purple: centromeric ReDFAS; blue: segments not labeled as ReDFAS.

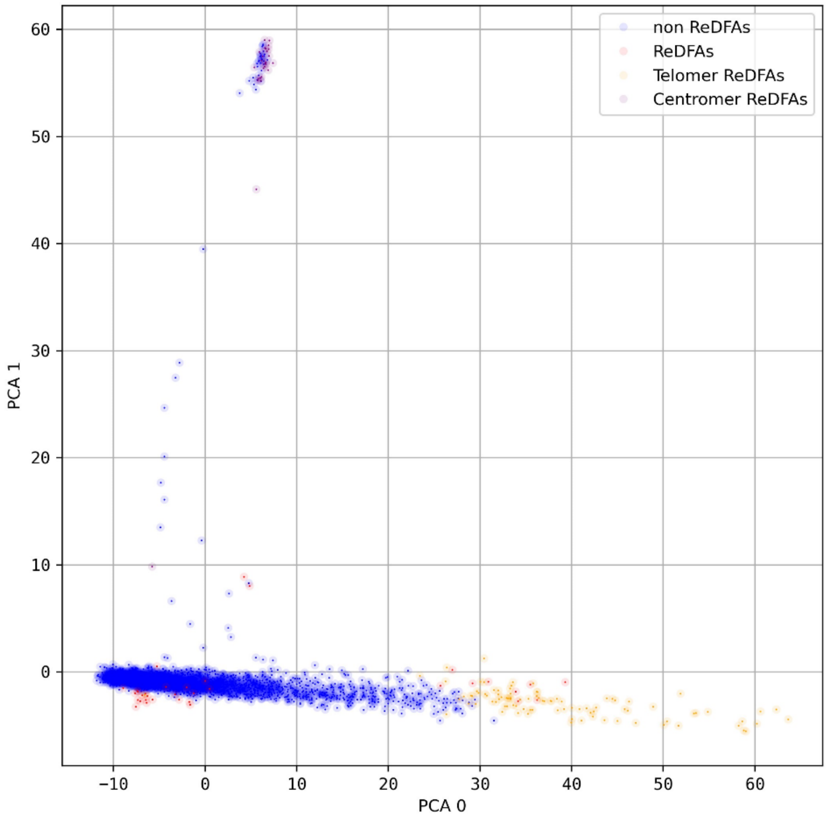

Figure S54. PCA results k = 5 on chromosome 9: PCA was performed on k = 5 spectra of 40 kbp segments on the chromosome. Different classes of regions were labeled with different colors. red: intermediate ReDFAS without special label; yellow: subtelomeric ReDFAS; purple: centromeric ReDFAS; blue: segments not labeled as ReDFAS.

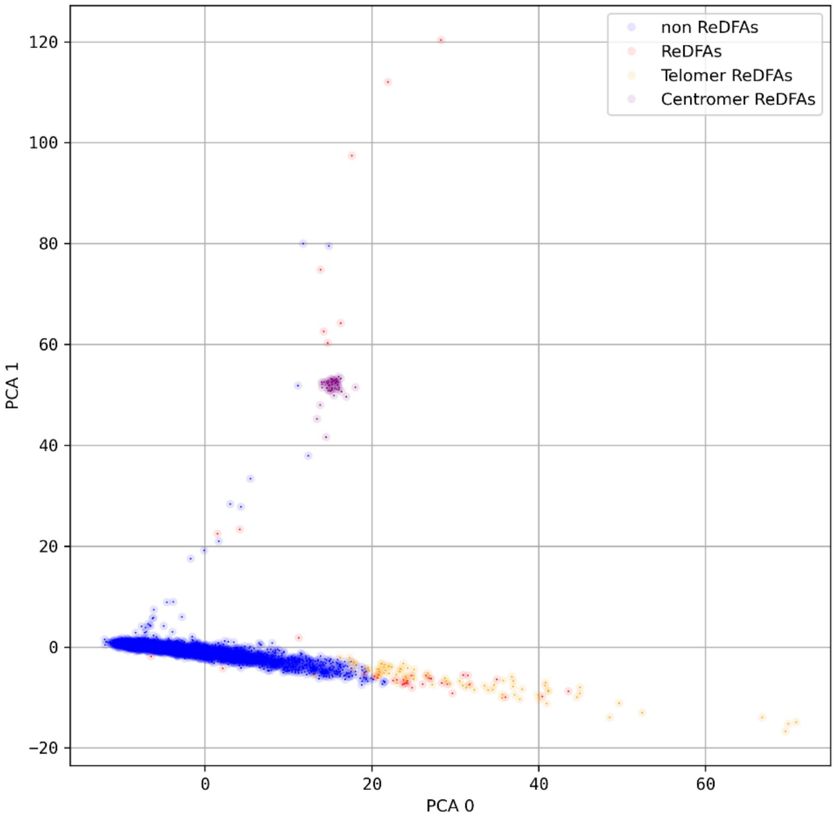

Figure S55. PCA results k = 5 on chromosome 10: PCA was performed on k = 5 spectra of 40 kbp segments on the chromosome. Different classes of regions were labeled with different colors. red: intermediate ReDFAS without special label; yellow: subtelomeric ReDFAS; purple: centromeric ReDFAS; blue: segments not labeled as ReDFAS.

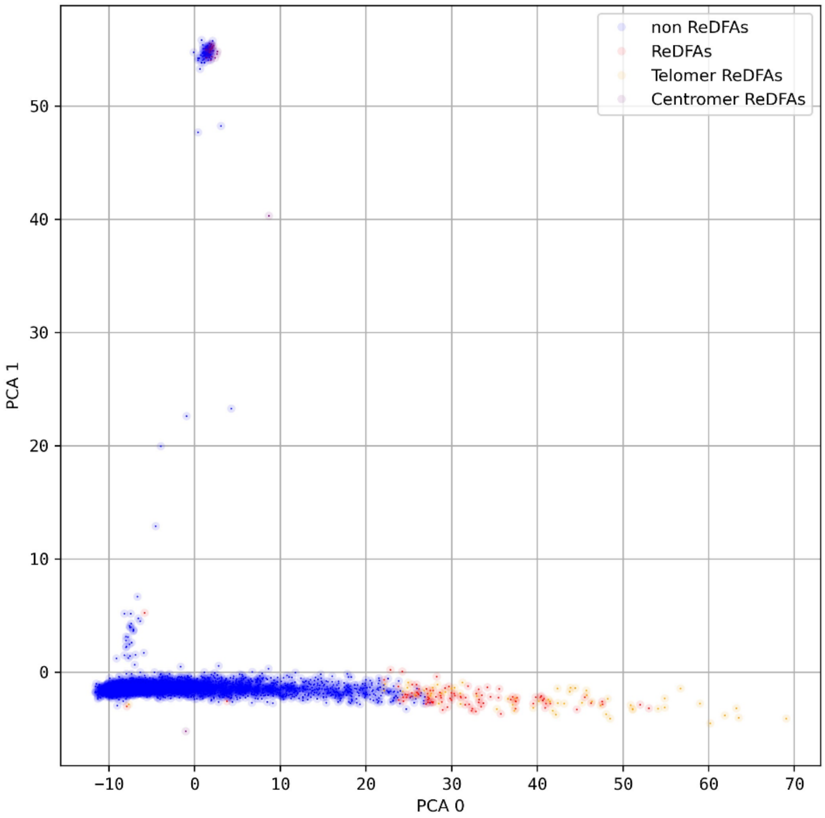

Figure S56. PCA results k = 5 on chromosome 11: PCA was performed on k = 5 spectra of 40 kbp segments on the chromosome. Different classes of regions were labeled with different colors. red: intermediate ReDFAS without special label; yellow: subtelomeric ReDFAS; purple: centromeric ReDFAS; blue: segments not labeled as ReDFAS.

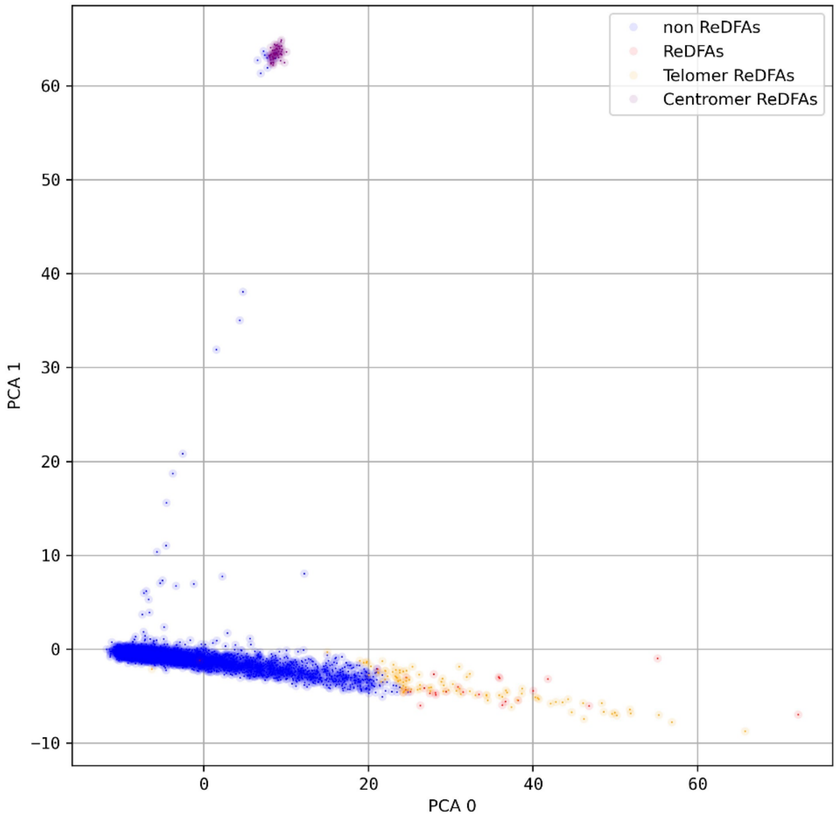

Figure S57. PCA results k = 5 on chromosome 12: PCA was performed on k = 5 spectra of 40 kbp segments on the chromosome. Different classes of regions were labeled with different colors. red: intermediate ReDFAS without special label; yellow: subtelomeric ReDFAS; purple: centromeric ReDFAS; blue: segments not labeled as ReDFAS.

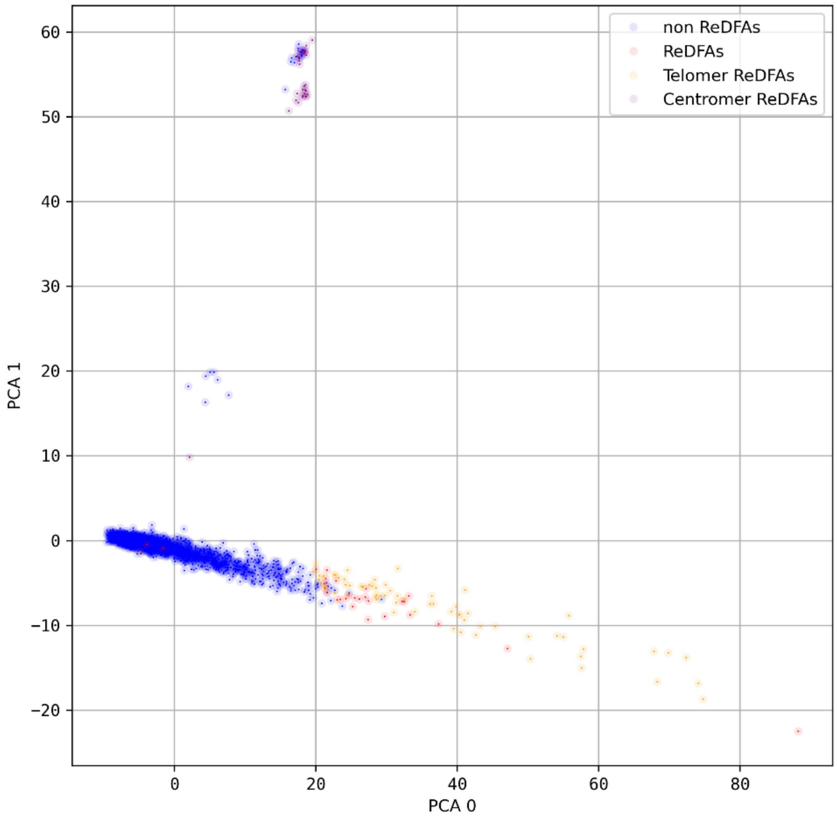

Figure S58. PCA results k = 5 on chromosome 13: PCA was performed on k = 5 spectra of 40 kbp segments on the chromosome. Different classes of regions were labeled with different colors. red: intermediate ReDFAS without special label; yellow: subtelomeric ReDFAS; purple: centromeric ReDFAS; blue: segments not labeled as ReDFAS.

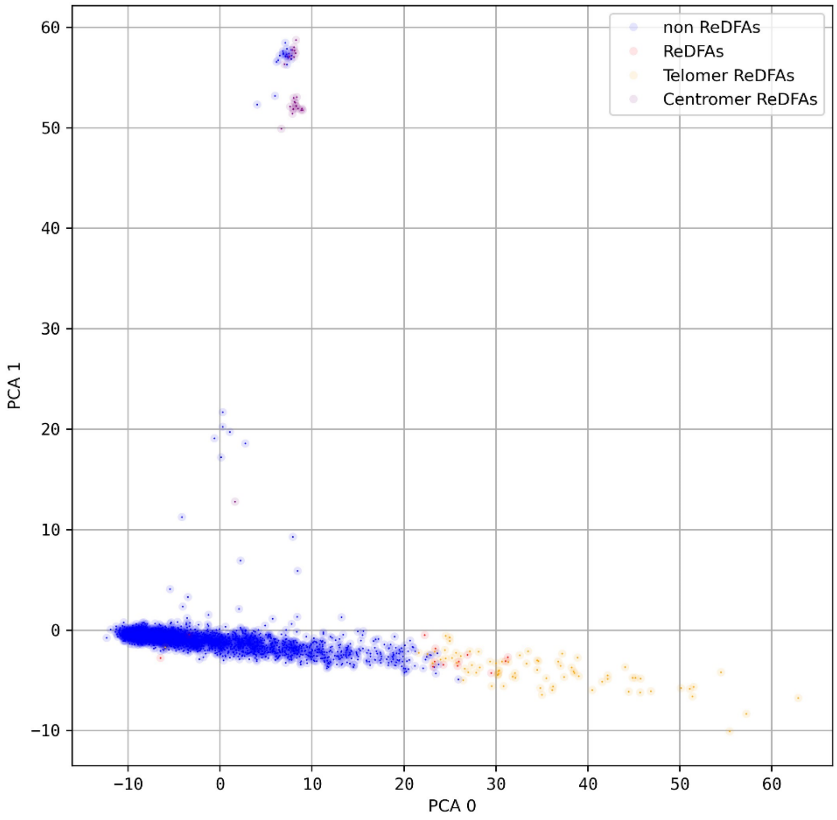

Figure S59. PCA results k = 5 on chromosome 14: PCA was performed on k = 5 spectra of 40 kbp segments on the chromosome. Different classes of regions were labeled with different colors. red: intermediate ReDFAS without special label; yellow: subtelomeric ReDFAS; purple: centromeric ReDFAS; blue: segments not labeled as ReDFAS.

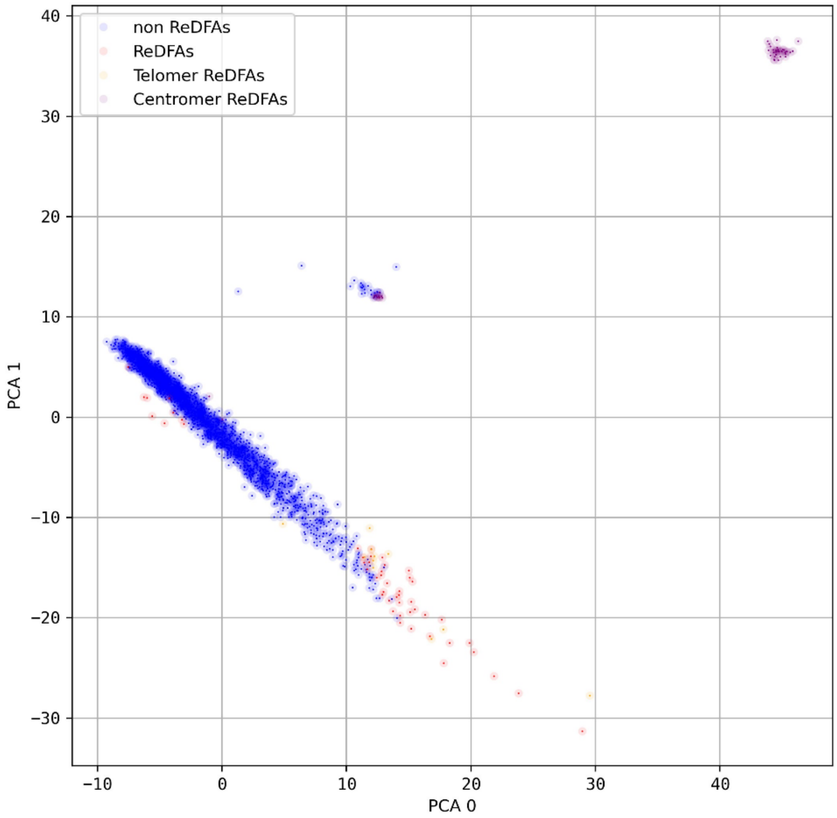

Figure S60. PCA results k = 5 on chromosome 15: PCA was performed on k = 5 spectra of 40 kbp segments on the chromosome. Different classes of regions were labeled with different colors. red: intermediate ReDFAS without special label; yellow: subtelomeric ReDFAS; purple: centromeric ReDFAS; blue: segments not labeled as ReDFAS.

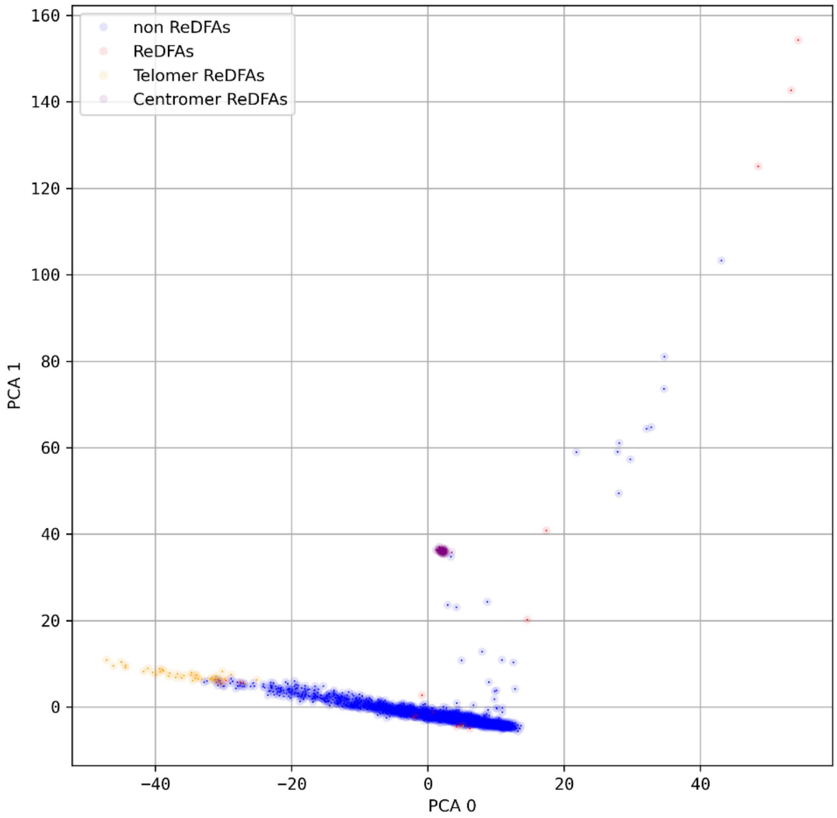

Figure S61. PCA results k = 5 on chromosome 16: PCA was performed on k = 5 spectra of 40 kbp segments on the chromosome. Different classes of regions were labeled with different colors. red: intermediate ReDFAS without special label; yellow: subtelomeric ReDFAS; purple: centromeric ReDFAS; blue: segments not labeled as ReDFAS.

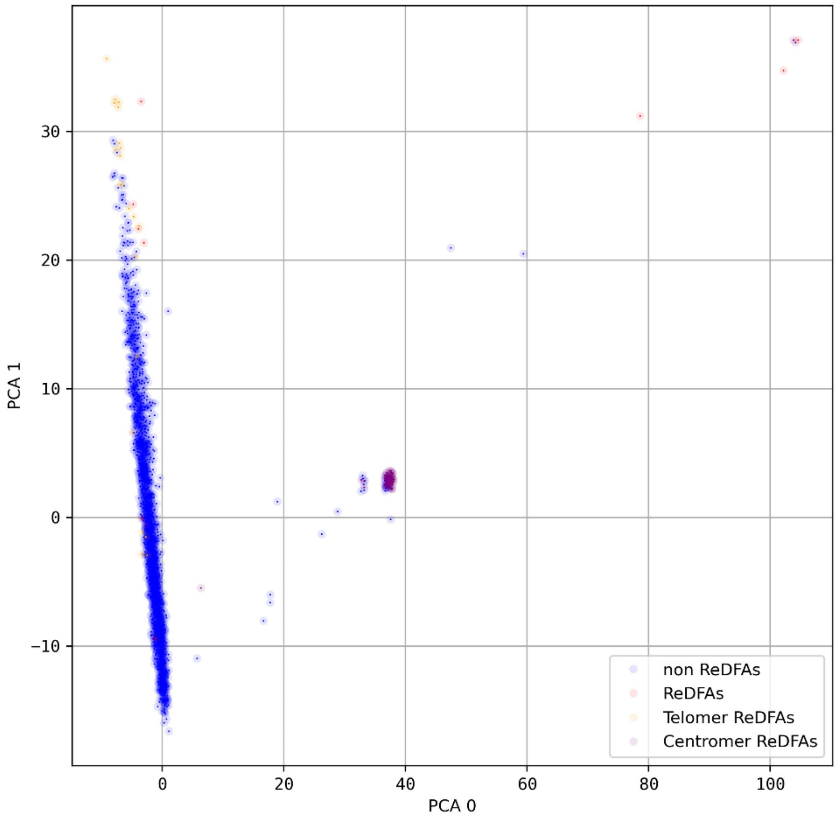

Figure S62. PCA results k = 5 on chromosome 17: PCA was performed on k = 5 spectra of 40 kbp segments on the chromosome. Different classes of regions were labeled with different colors. red: intermediate ReDFAS without special label; yellow: subtelomeric ReDFAS; purple: centromeric ReDFAS; blue: segments not labeled as ReDFAS.

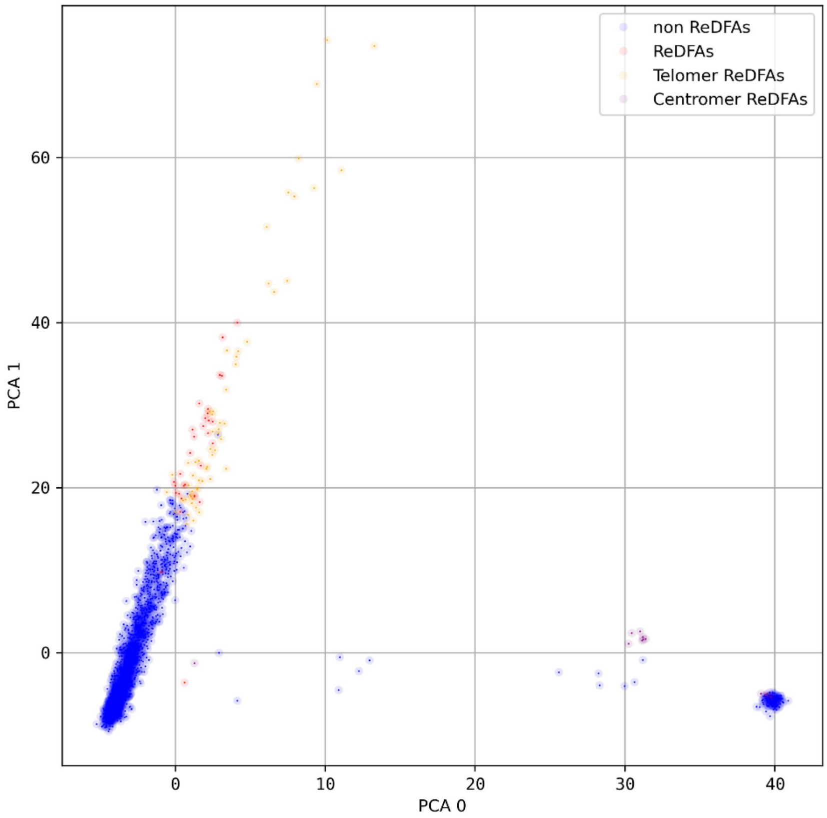

Figure S63. PCA results k = 5 on chromosome 18: PCA was performed on k = 5 spectra of 40 kbp segments on the chromosome. Different classes of regions were labeled with different colors. red: intermediate ReDFAS without special label; yellow: subtelomeric ReDFAS; purple: centromeric ReDFAS; blue: segments not labeled as ReDFAS.

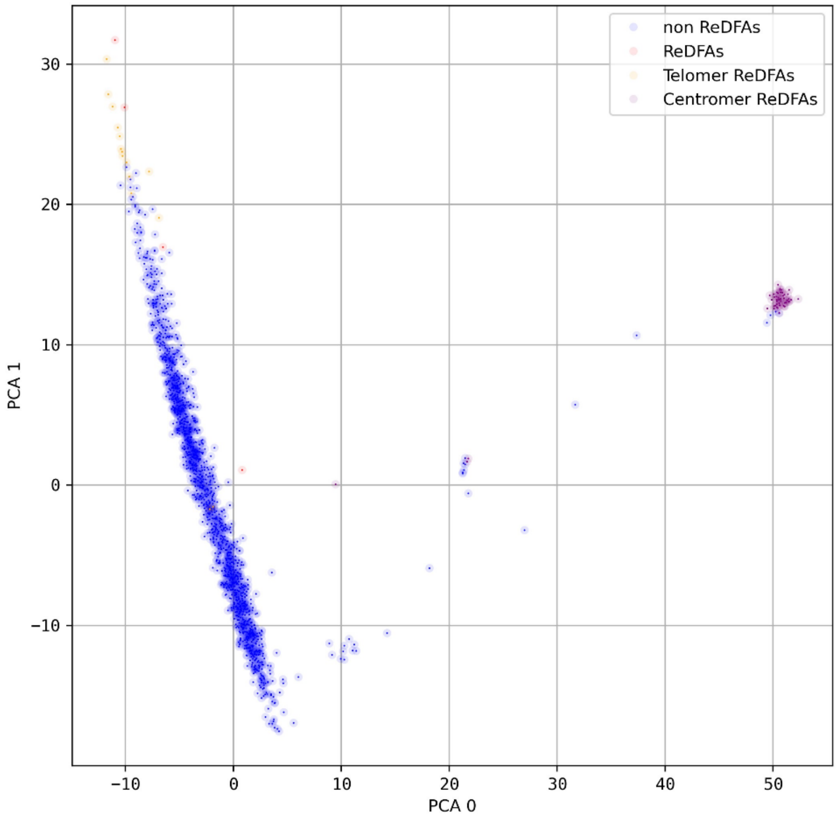

Figure S64. PCA results k = 5 on chromosome 19: PCA was performed on k = 5 spectra of 40 kbp segments on the chromosome. Different classes of regions were labeled with different colors. red: intermediate ReDFAS without special label; yellow: subtelomeric ReDFAS; purple: centromeric ReDFAS; blue: segments not labeled as ReDFAS.

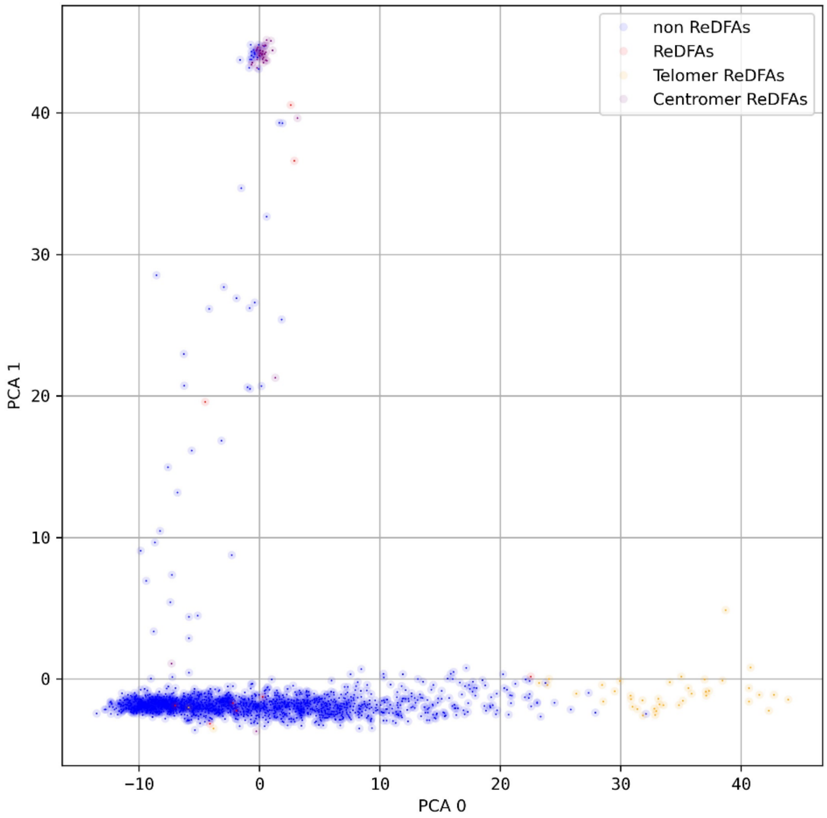

Figure S65. PCA results k = 5 on chromosome 20: PCA was performed on k = 5 spectra of 40 kbp segments on the chromosome. Different classes of regions were labeled with different colors. red: intermediate ReDFAS without special label; yellow: subtelomeric ReDFAS; purple: centromeric ReDFAS; blue: segments not labeled as ReDFAS.

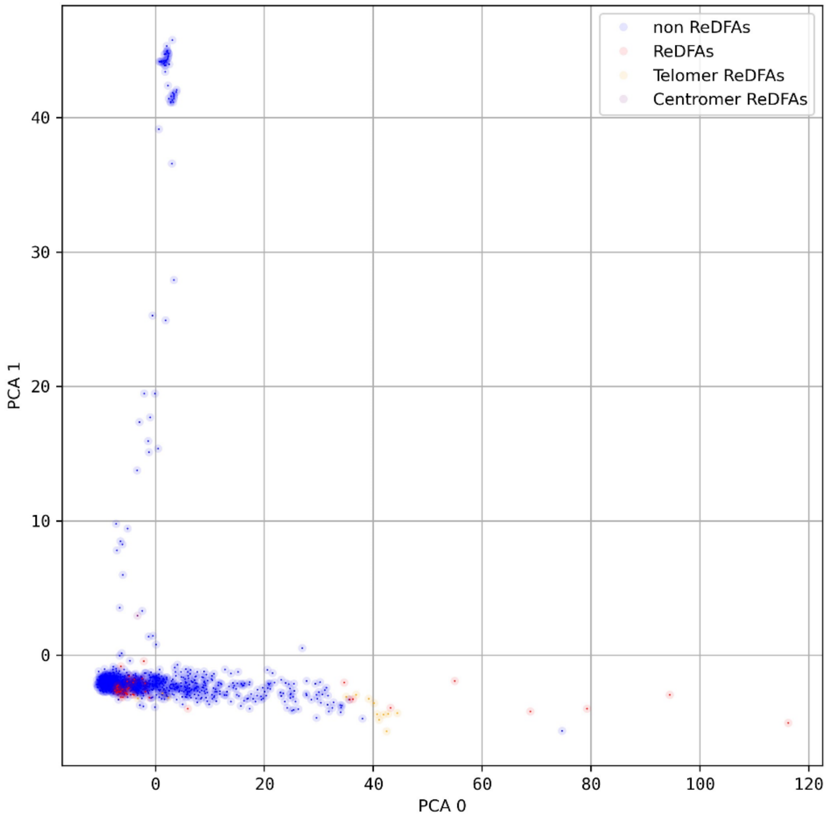

Figure S66. PCA results k = 5 on chromosome 21: PCA was performed on k = 5 spectra of 40 kbp segments on the chromosome. Different classes of regions were labeled with different colors. red: intermediate ReDFAS without special label; yellow: subtelomeric ReDFAS; purple: centromeric ReDFAS; blue: segments not labeled as ReDFAS.

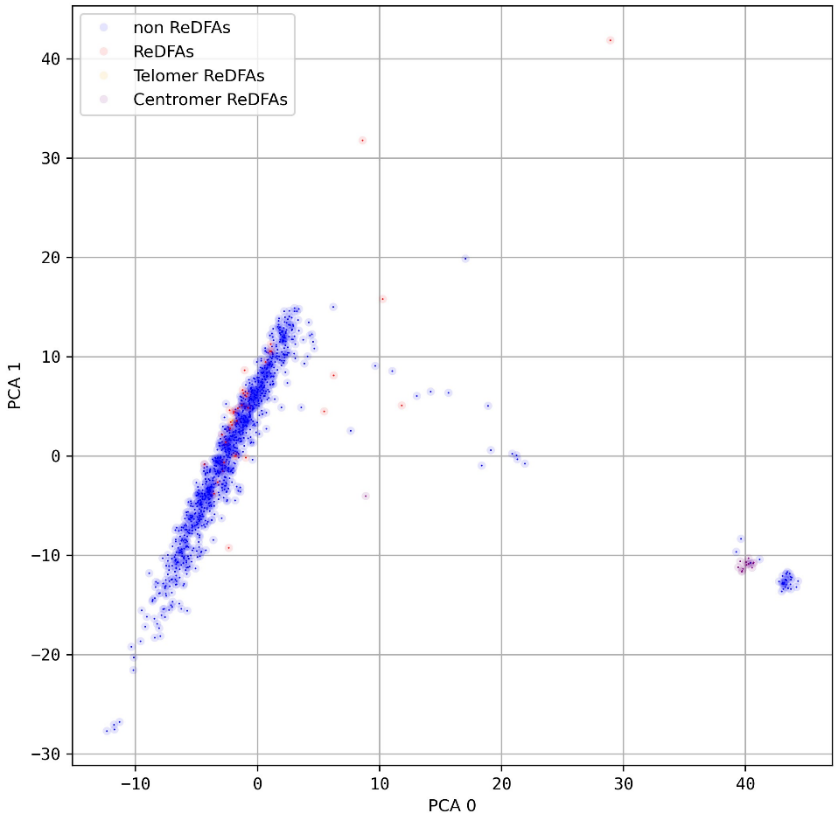

Figure S67. PCA results k = 5 on chromosome 22: PCA was performed on k = 5 spectra of 40 kbp segments on the chromosome. Different classes of regions were labeled with different colors. red: intermediate ReDFAS without special label; yellow: subtelomeric ReDFAS; purple: centromeric ReDFAS; blue: segments not labeled as ReDFAS.

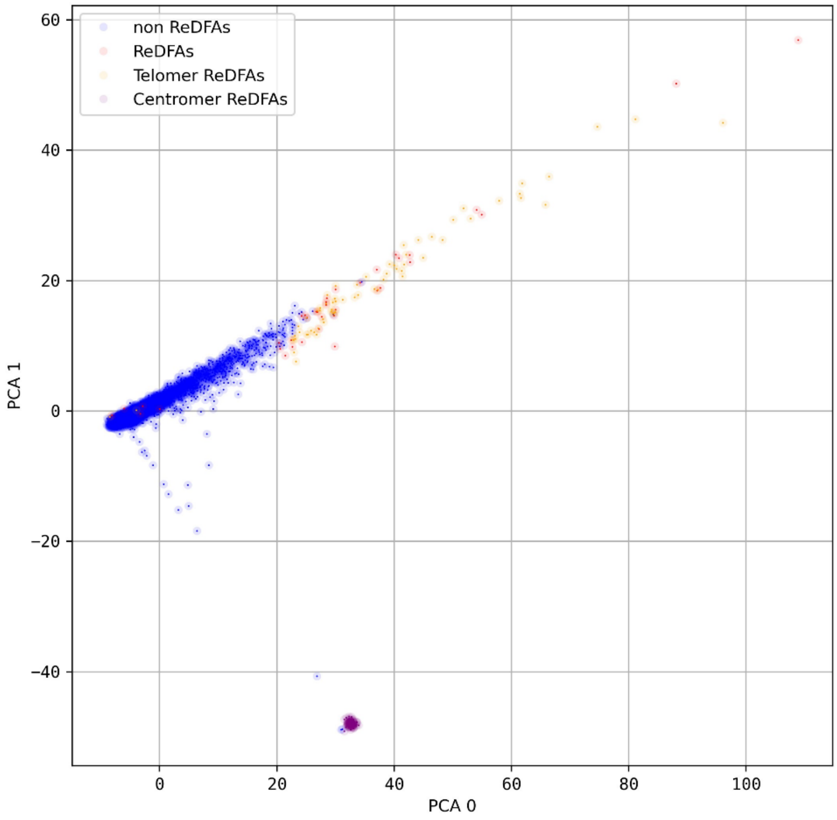

Figure S68. PCA results k = 5 on chromosome X: PCA was performed on k = 5 spectra of 40 kbp segments on the chromosome. Different classes of regions were labeled with different colors. red: intermediate ReDFAS without special label; yellow: subtelomeric ReDFAS; purple: centromeric ReDFAS; blue: segments not labeled as ReDFAS.

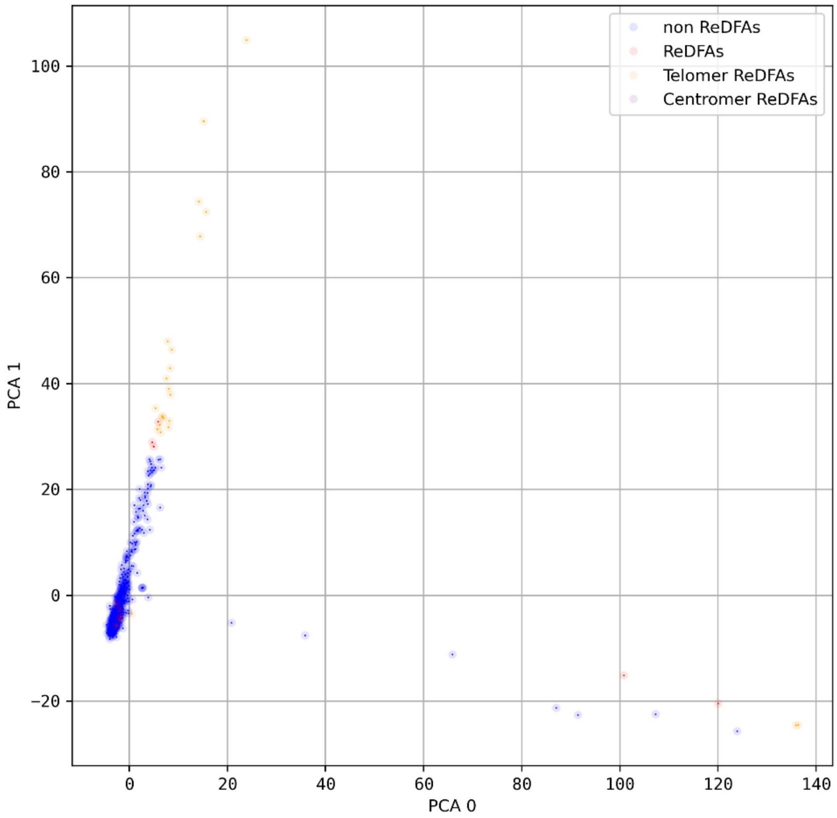

Figure S69. PCA results k = 5 on chromosome Y: PCA was performed on k = 5 spectra of 40 kbp segments on the chromosome. Different classes of regions were labeled with different colors. red: intermediate ReDFAS without special label; yellow: subtelomeric ReDFAS; purple: centromeric ReDFAS; blue: segments not labeled as ReDFAS.

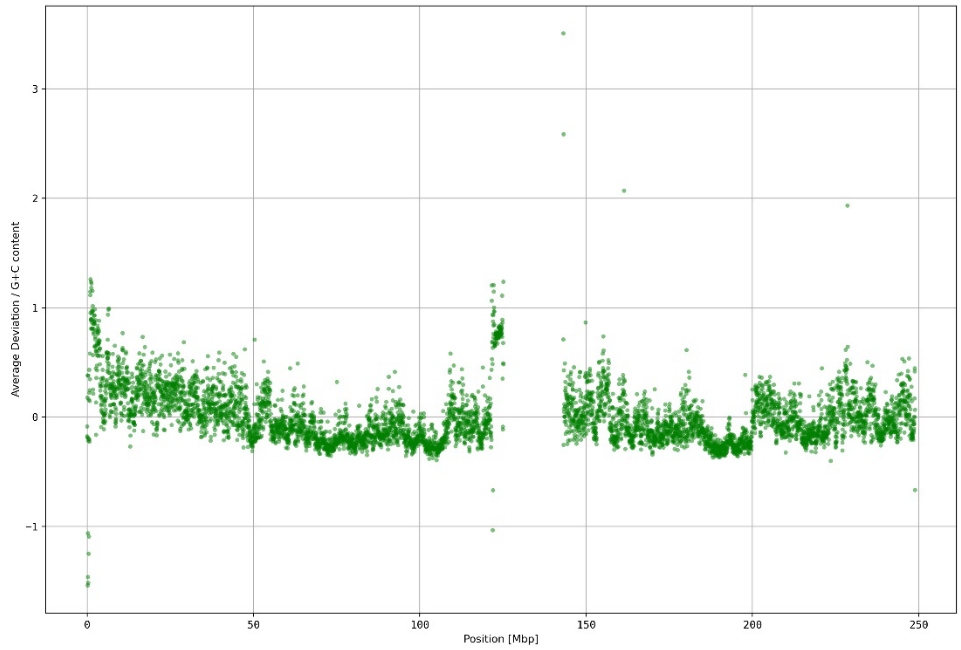

Figure S70. Relation between ReDFAS and G+C Content. Average deviation from average spectra of 40 kb segments on chromosome 1, corrected for local G+C content.

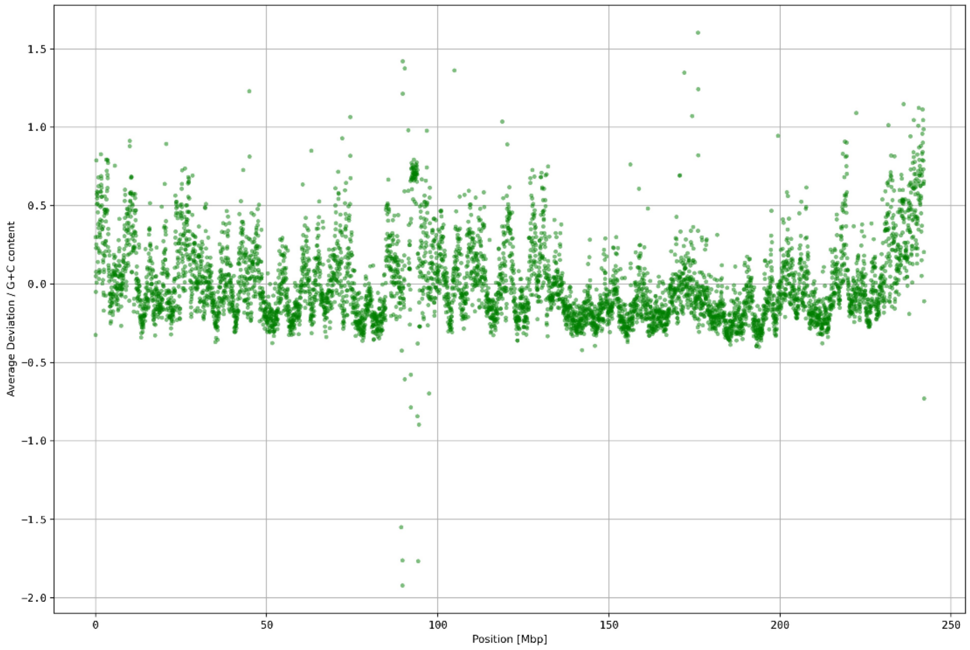

Figure S71. Relation between ReDFAS and G+C Content. Average deviation from average spectra of 40 kb segments on chromosome 2, corrected for local G+C content.

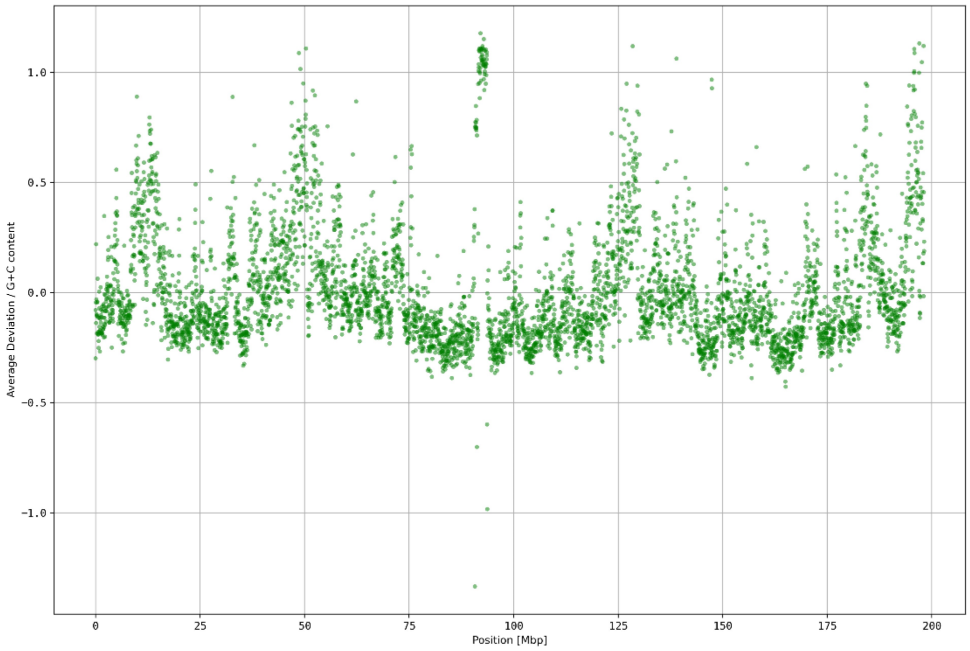

Figure S72. Relation between ReDFAS and G+C Content. Average deviation from average spectra of 40 kb segments on chromosome 3, corrected for local G+C content.

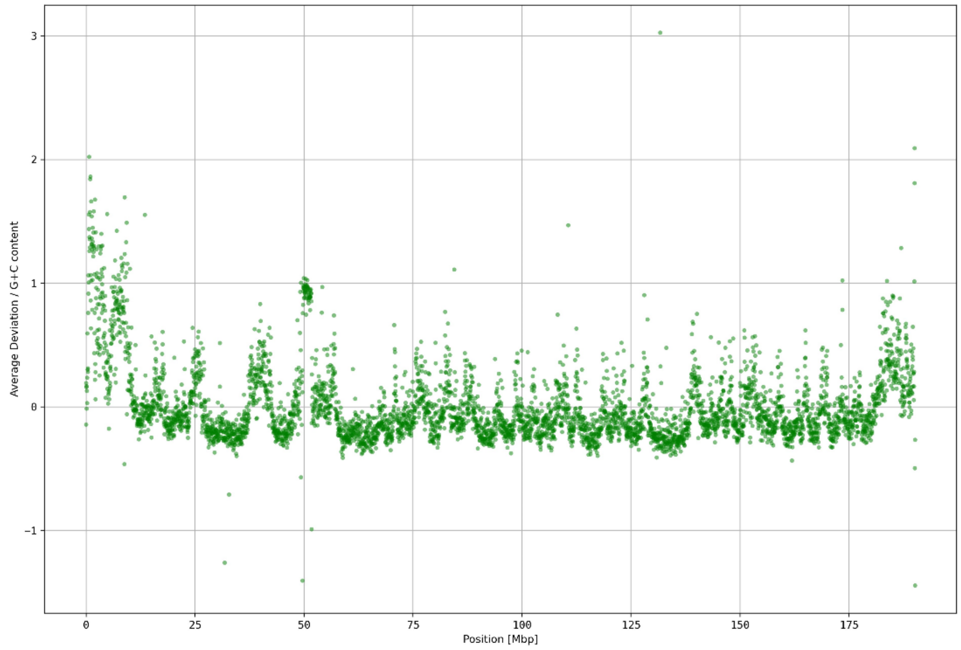

Figure S73. Relation between ReDFAS and G+C Content. Average deviation from average spectra of 40 kb segments on chromosome 4, corrected for local G+C content.

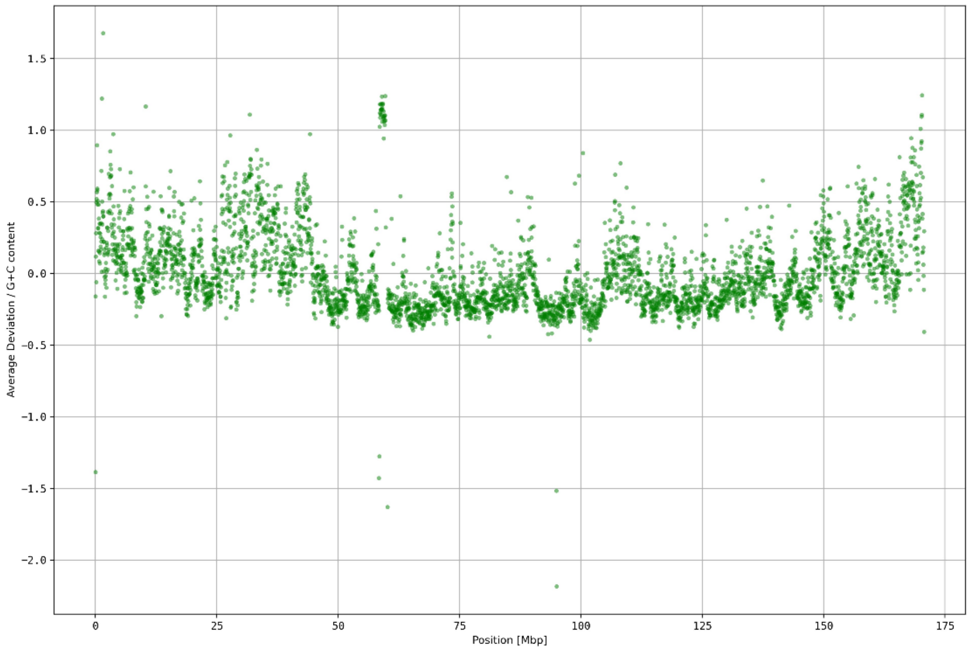

Figure S74. Relation between ReDFAS and G+C Content. Average deviation from average spectra of 40 kb segments on chromosome 6, corrected for local G+C content.

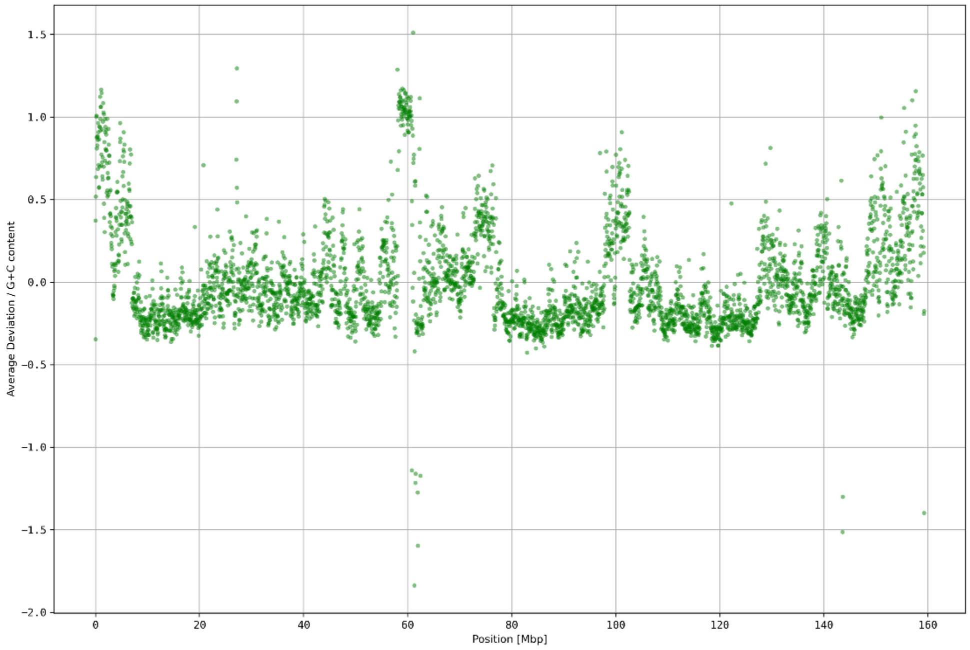

Figure S75. Relation between ReDFAS and G+C Content. Average deviation from average spectra of 40 kb segments on chromosome 7, corrected for local G+C content.

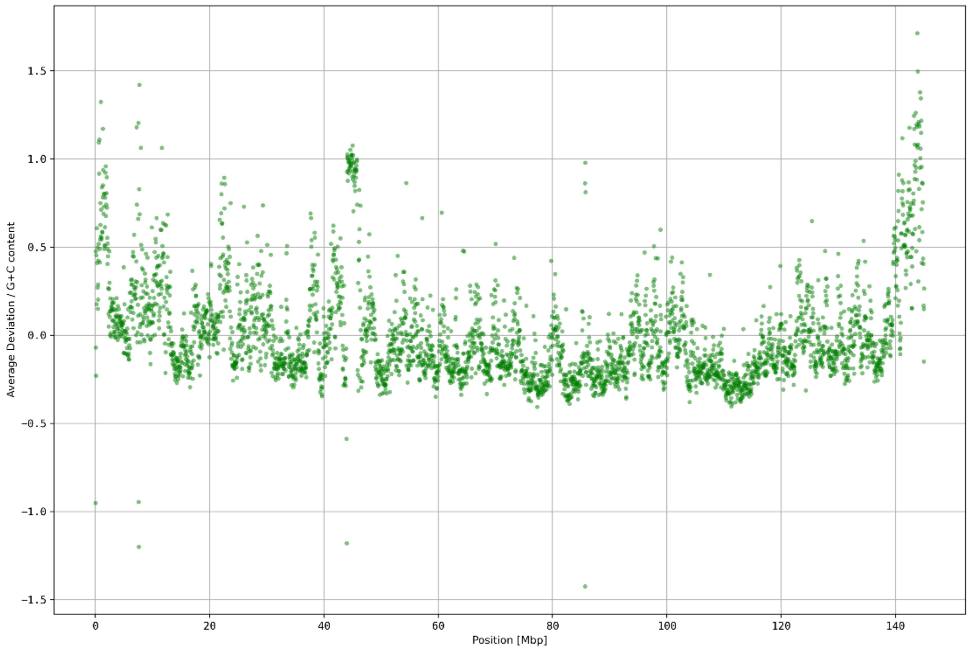

Figure S76. Relation between ReDFAS and G+C Content. Average deviation from average spectra of 40 kb segments on chromosome 8, corrected for local G+C content.

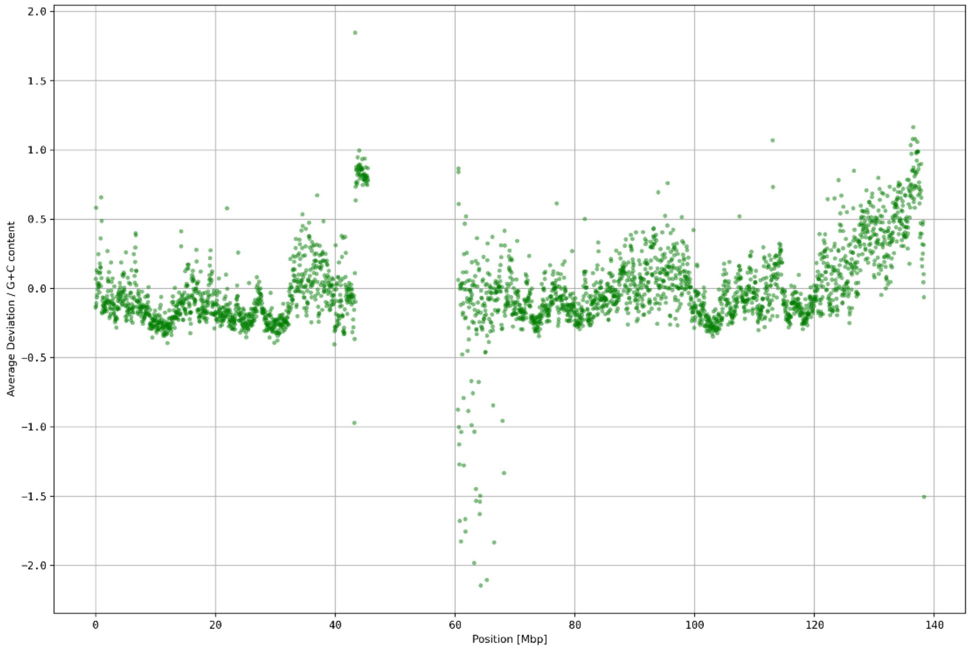

Figure S77. Relation between ReDFAS and G+C Content. Average deviation from average spectra of 40 kb segments on chromosome 9, corrected for local G+C content.

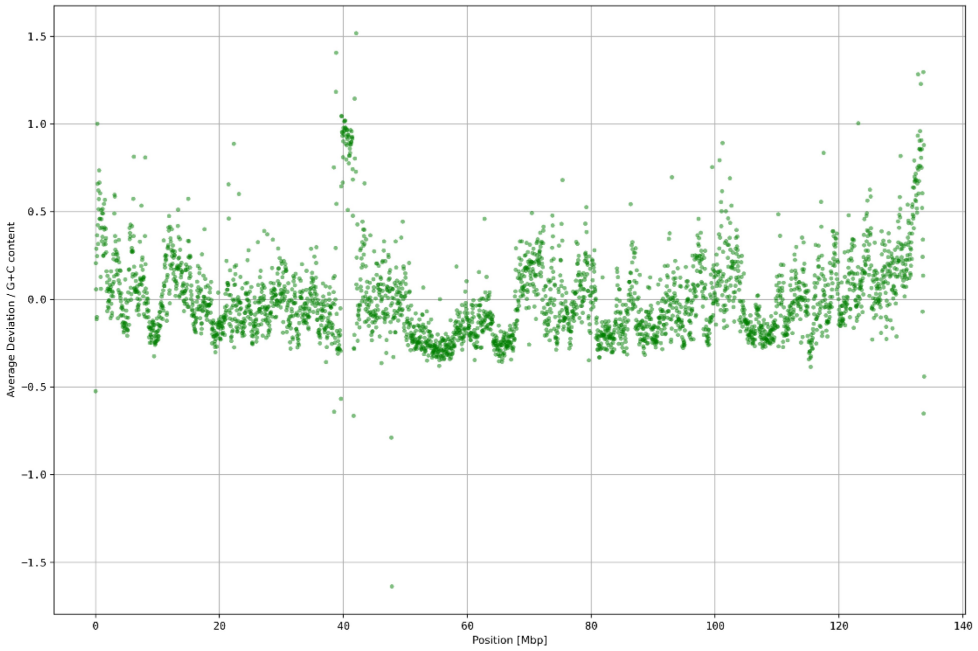

Figure S78. Relation between ReDFAS and G+C Content. Average deviation from average spectra of 40 kb segments on chromosome 10, corrected for local G+C content.

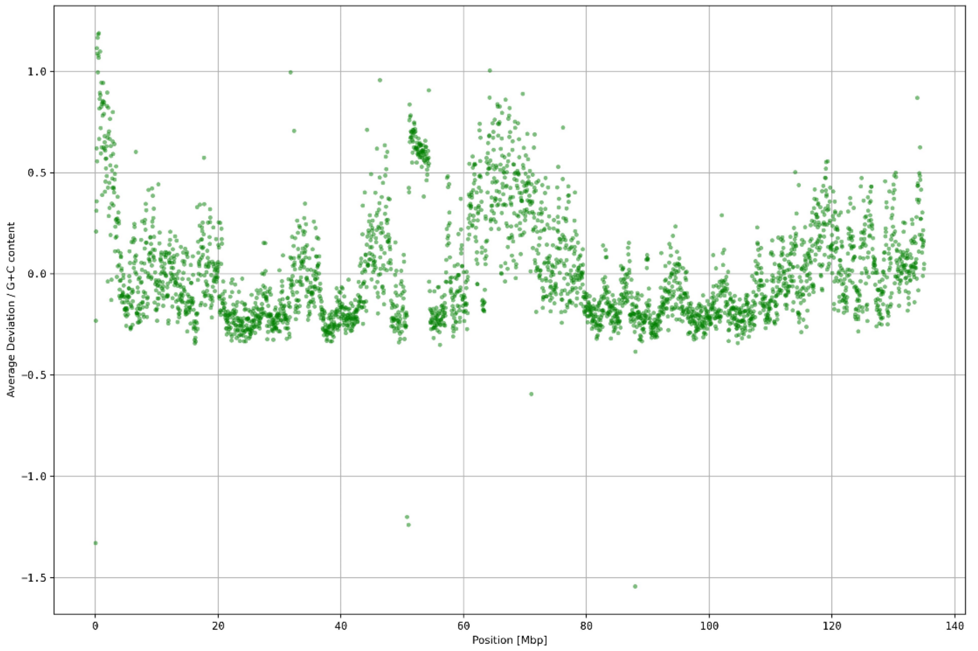

Figure S79. Relation between ReDFAS and G+C Content. Average deviation from average spectra of 40 kb segments on chromosome 11, corrected for local G+C content.

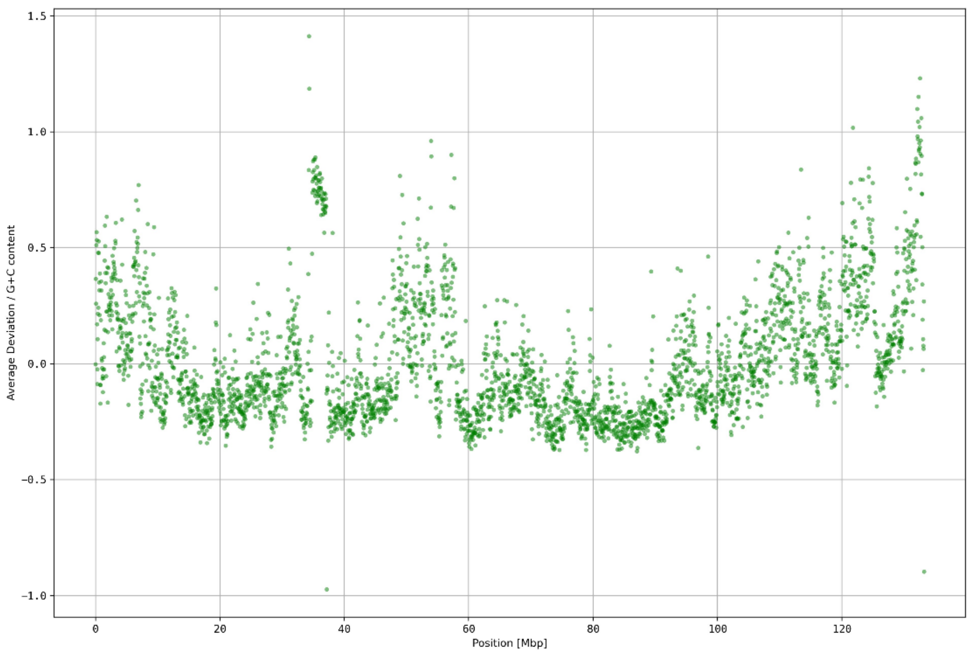

Figure S80. Relation between ReDFAS and G+C Content. Average deviation from average spectra of 40 kb segments on chromosome 12, corrected for local G+C content.

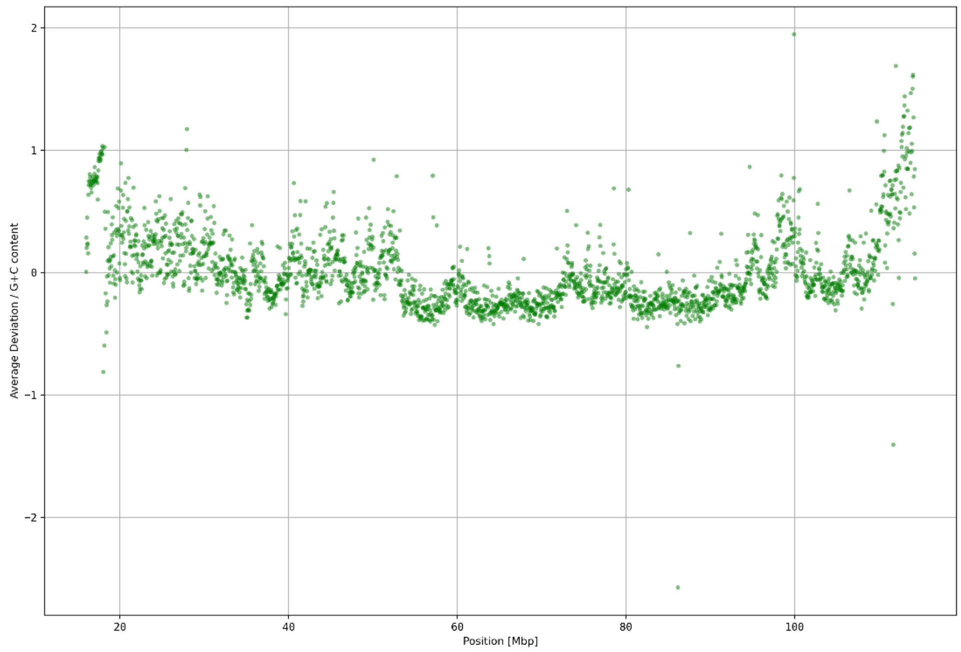

Figure S81. Relation between ReDFAS and G+C Content. Average deviation from average spectra of 40 kb segments on chromosome 13, corrected for local G+C content.

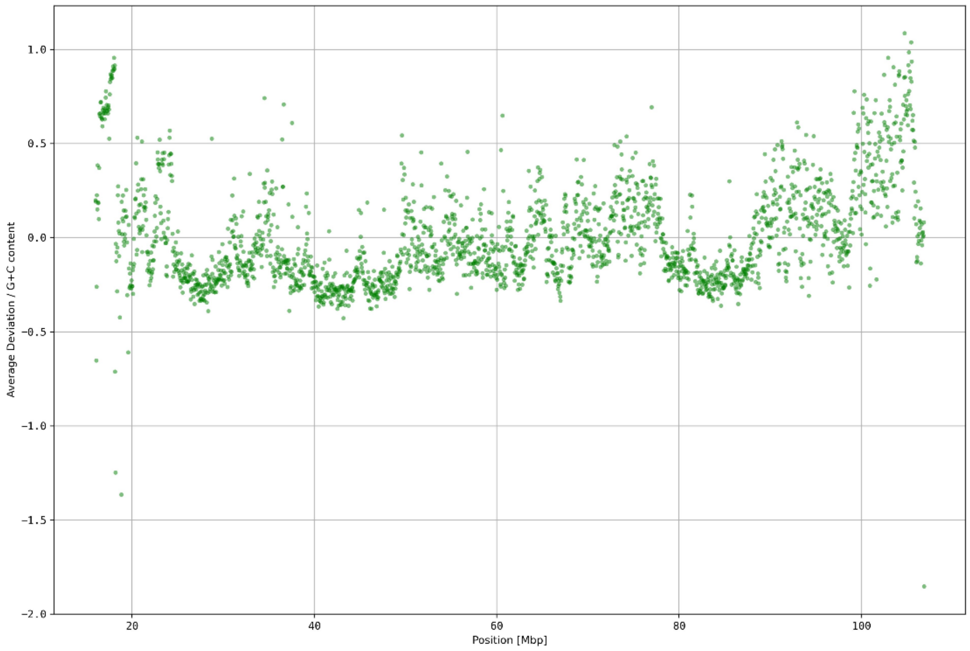

Figure S82. Relation between ReDFAS and G+C Content. Average deviation from average spectra of 40 kb segments on chromosome 14, corrected for local G+C content.

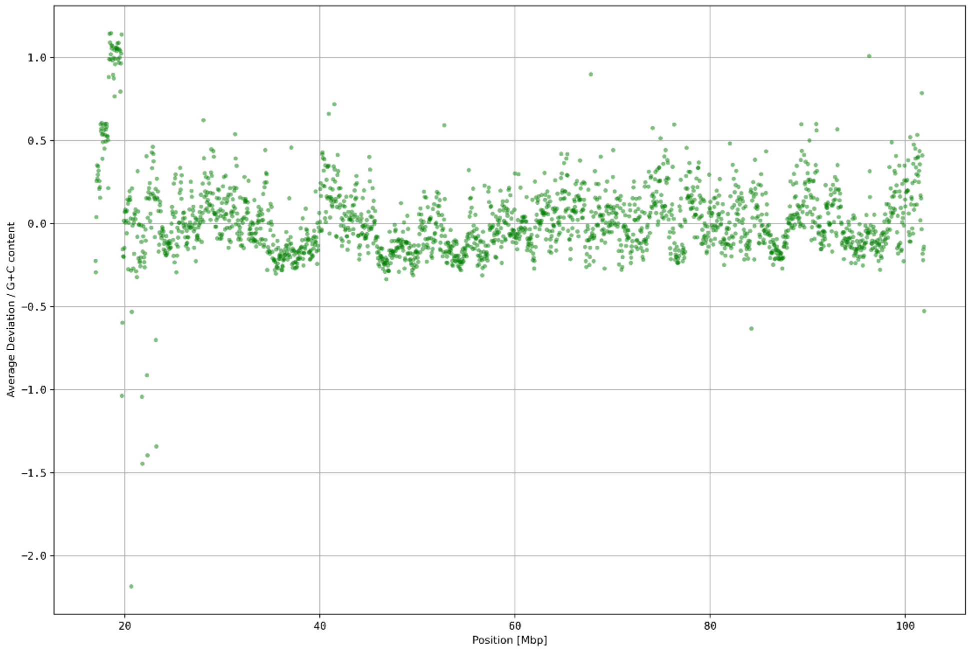

Figure S83. Relation between ReDFAS and G+C Content. Average deviation from average spectra of 40 kb segments on chromosome 15, corrected for local G+C content.

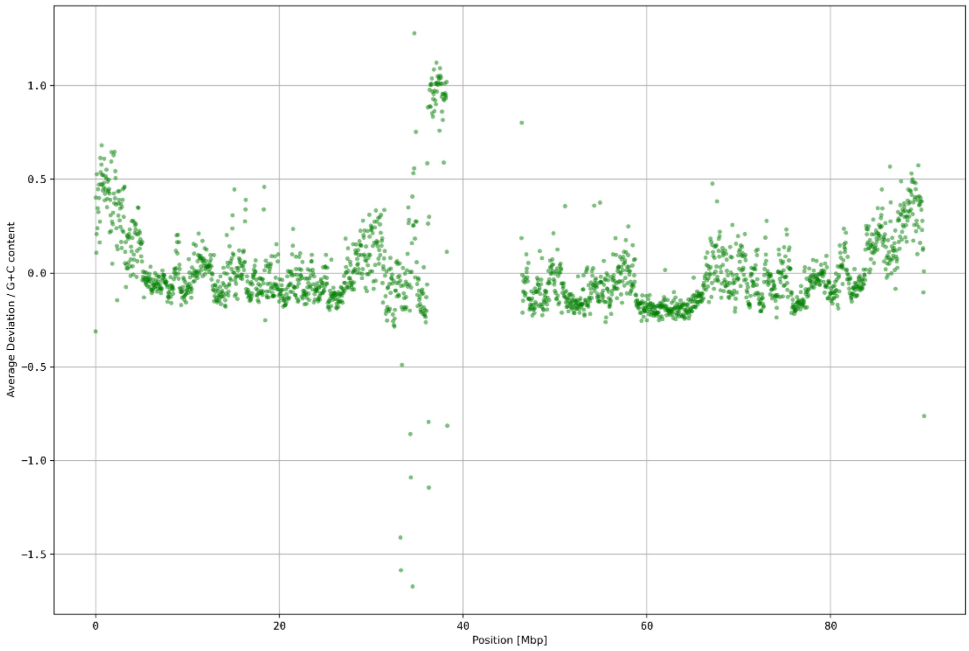

Figure S84. Relation between ReDFAS and G+C Content. Average deviation from average spectra of 40 kb segments on chromosome 16, corrected for local G+C content.

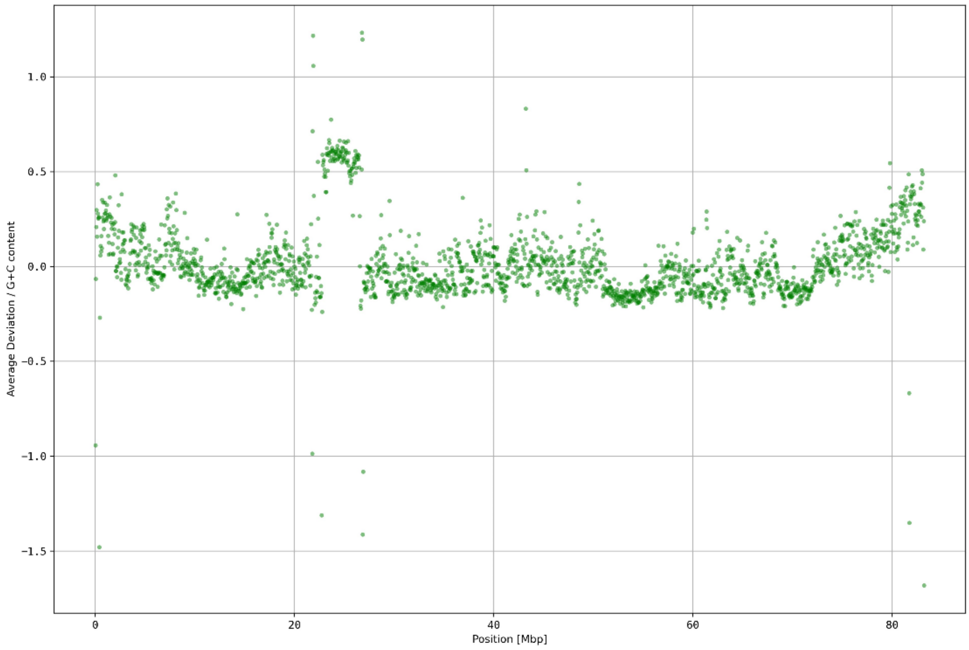

Figure S85. Relation between ReDFAS and G+C Content. Average deviation from average spectra of 40 kb segments on chromosome 17, corrected for local G+C content.

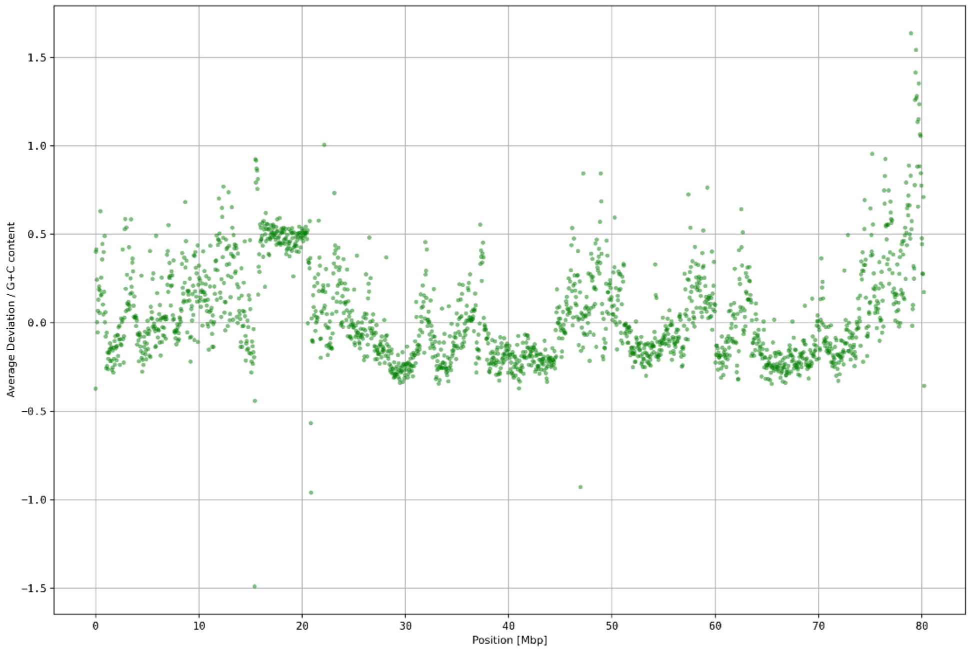

Figure S86. Relation between ReDFAS and G+C Content. Average deviation from average spectra of 40 kb segments on chromosome 18, corrected for local G+C content.

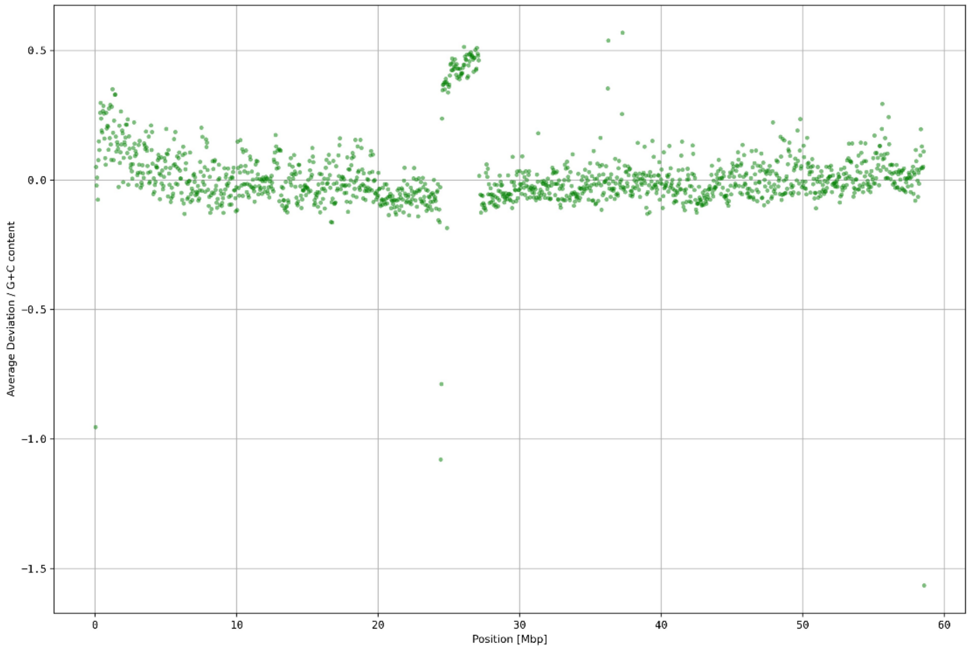

Figure S87. Relation between ReDFAS and G+C Content. Average deviation from average spectra of 40 kb segments on chromosome 19, corrected for local G+C content.

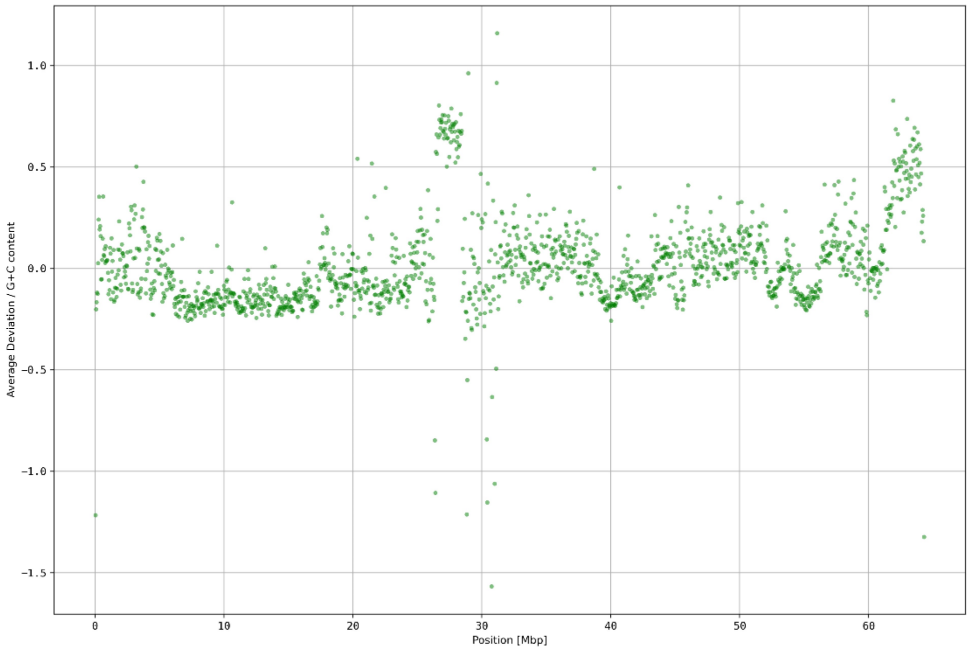

Figure S88. Relation between ReDFAS and G+C Content. Average deviation from average spectra of 40 kb segments on chromosome 20, corrected for local G+C content.

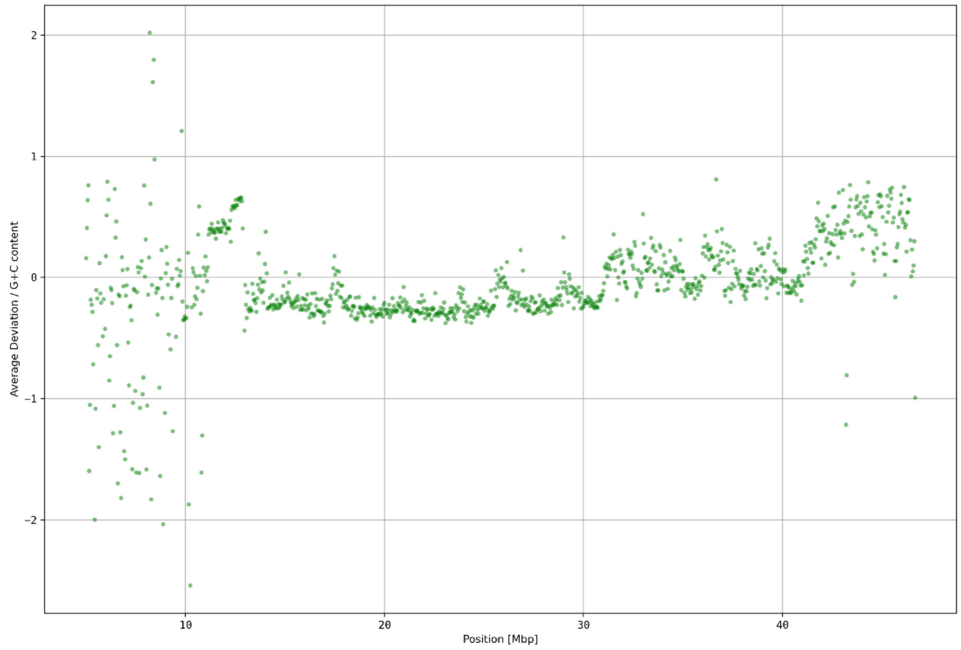

Figure S89. Relation between ReDFAS and G+C Content. Average deviation from average spectra of 40 kb segments on chromosome 21, corrected for local G+C content.

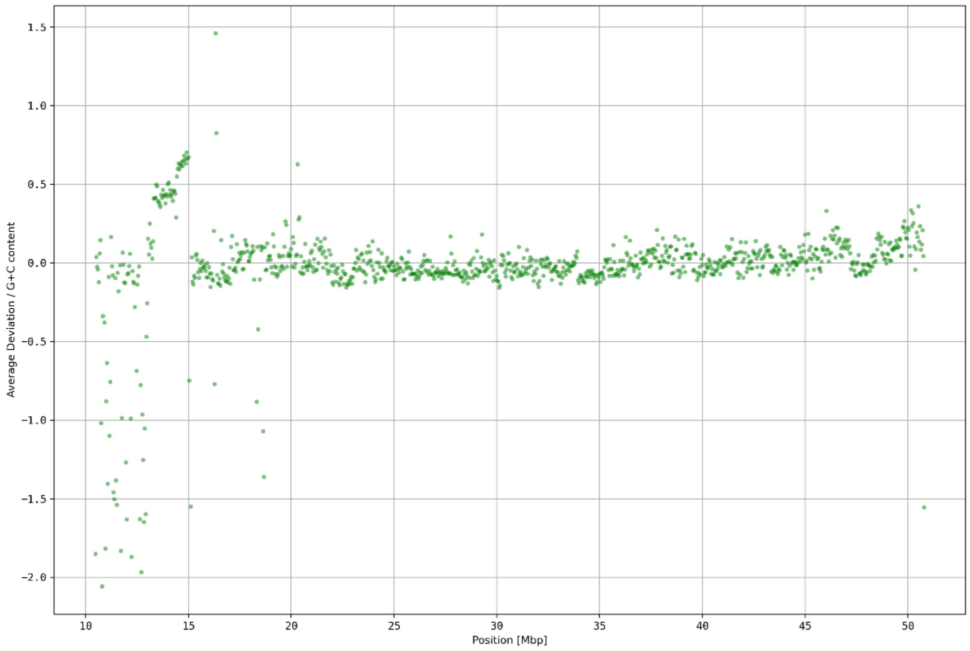

Figure S90. Relation between ReDFAS and G+C Content. Average deviation from average spectra of 40 kb segments on chromosome 22, corrected for local G+C content.

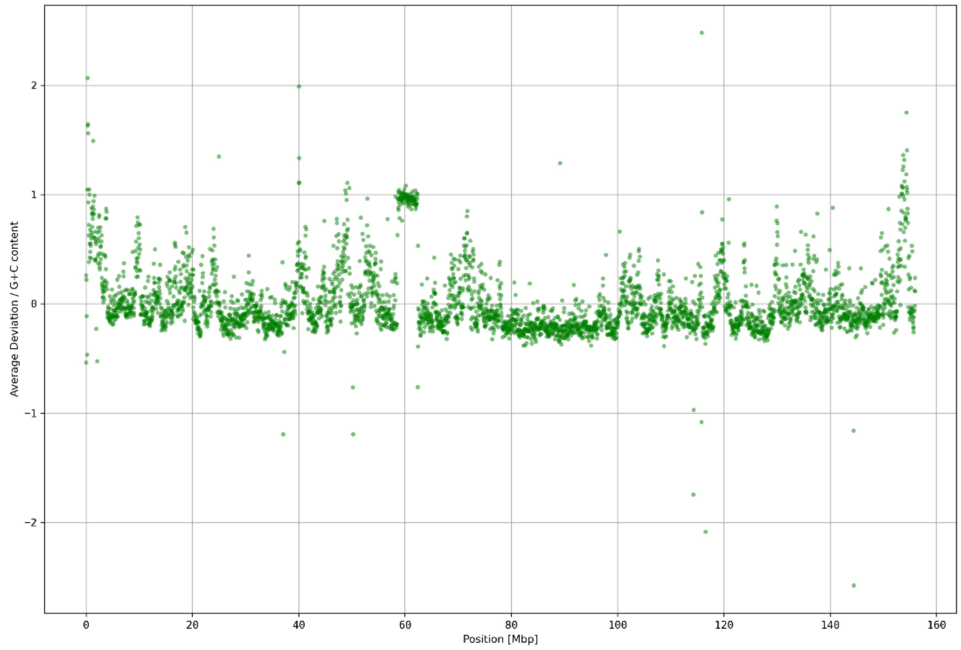

Figure S91. Relation between ReDFAS and G+C Content. Average deviation from average spectra of 40 kb segments on chromosome X, corrected for local G+C content.

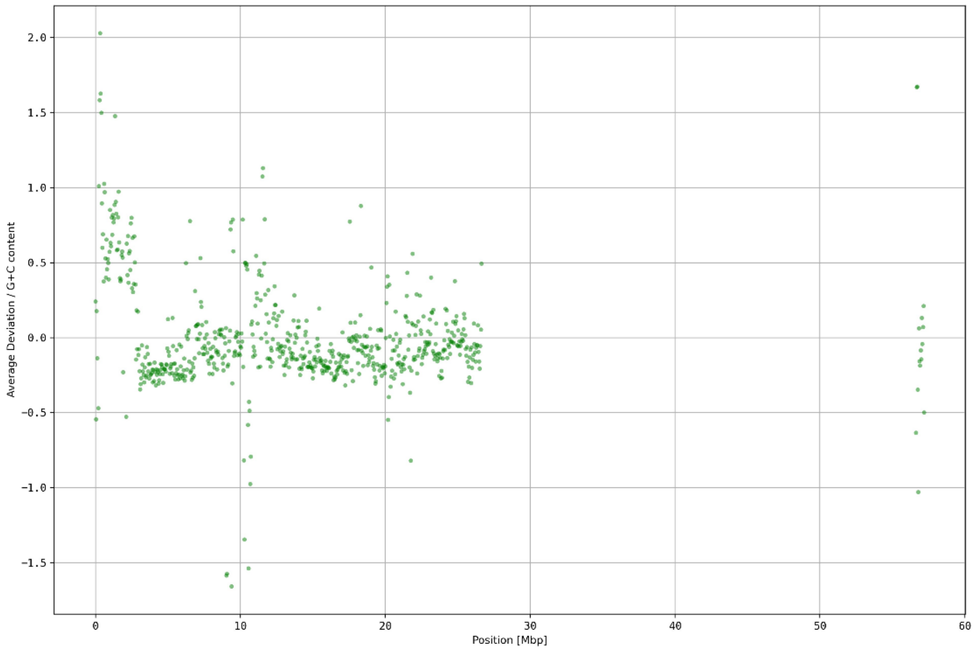

Figure S92. Relation between ReDFAS and G+C Content. Average deviation from average spectra of 40 kb segments on chromosome Y, corrected for local G+C content.

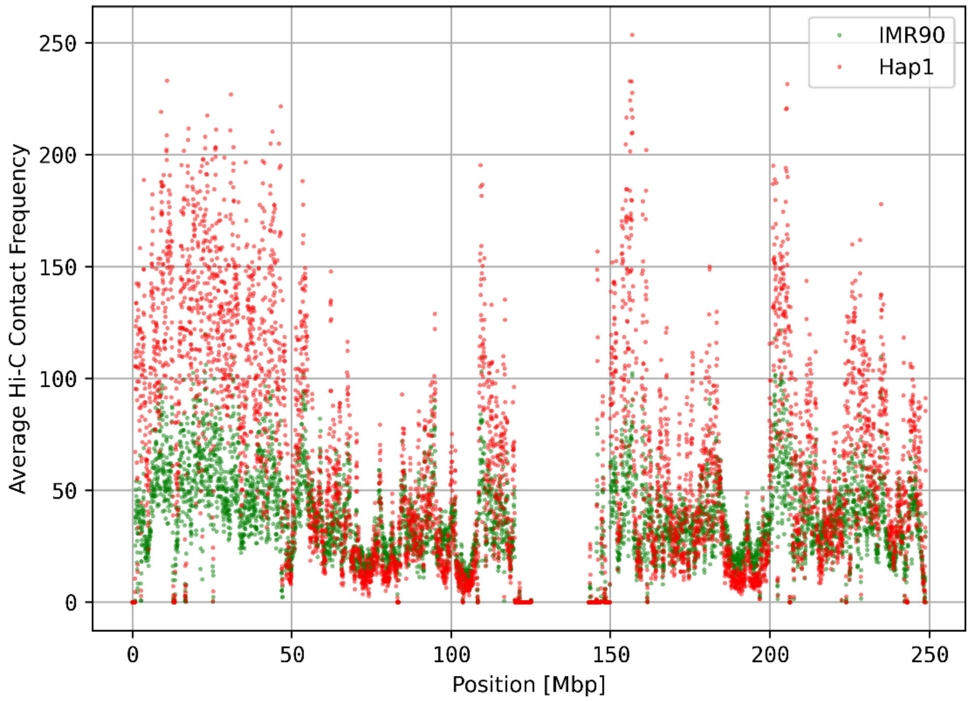

Figure S93: The Hi-C contact frequencies on chromosome 1 for different cell lines: (red) The Hi-C frequencies in HAP1. (green) The Hi-C frequencies in IMR90.

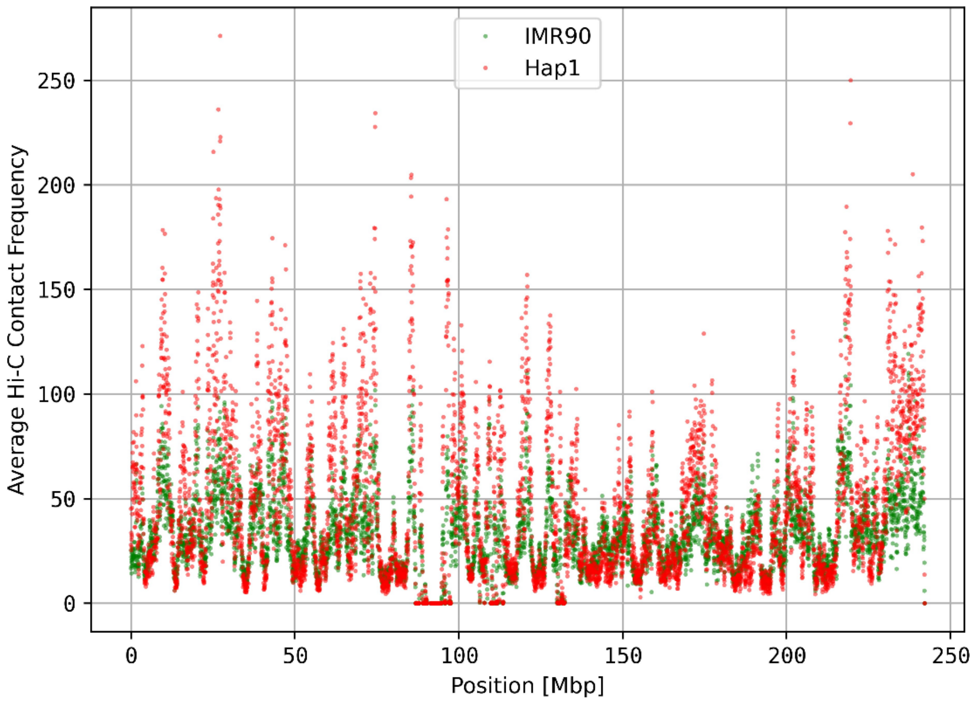

Figure S94: The Hi-C contact frequencies on chromosome 2 for different cell lines: (red) The Hi-C frequencies in HAP1. (green) The Hi-C frequencies in IMR90.

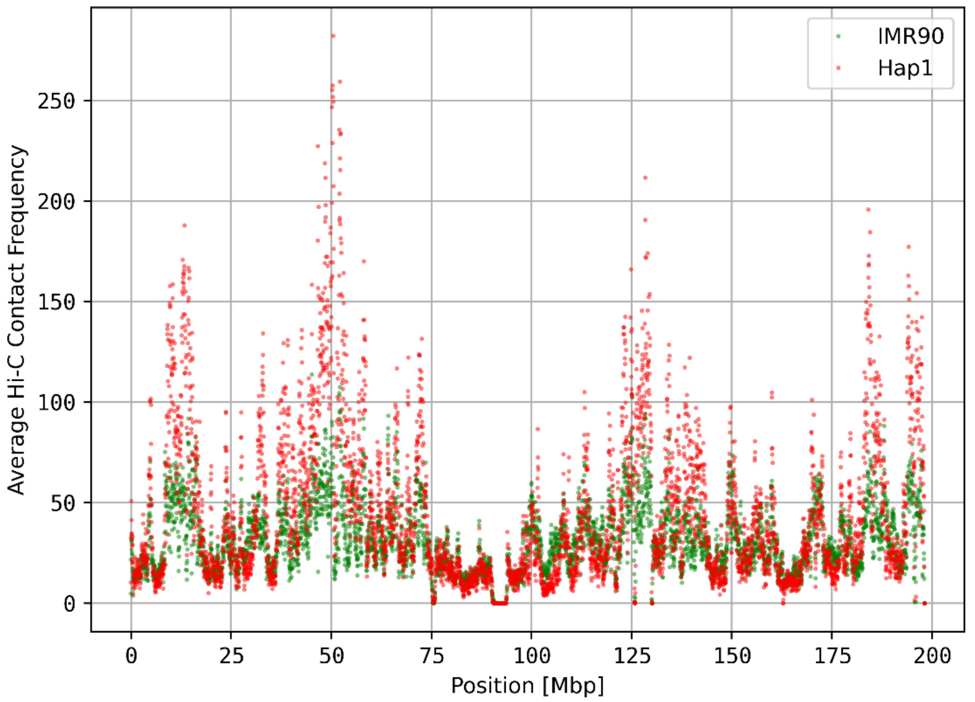

Figure S95: The Hi-C contact frequencies on chromosome 3 for different cell lines: (red) The Hi-C frequencies in HAP1. (green) The Hi-C frequencies in IMR90.

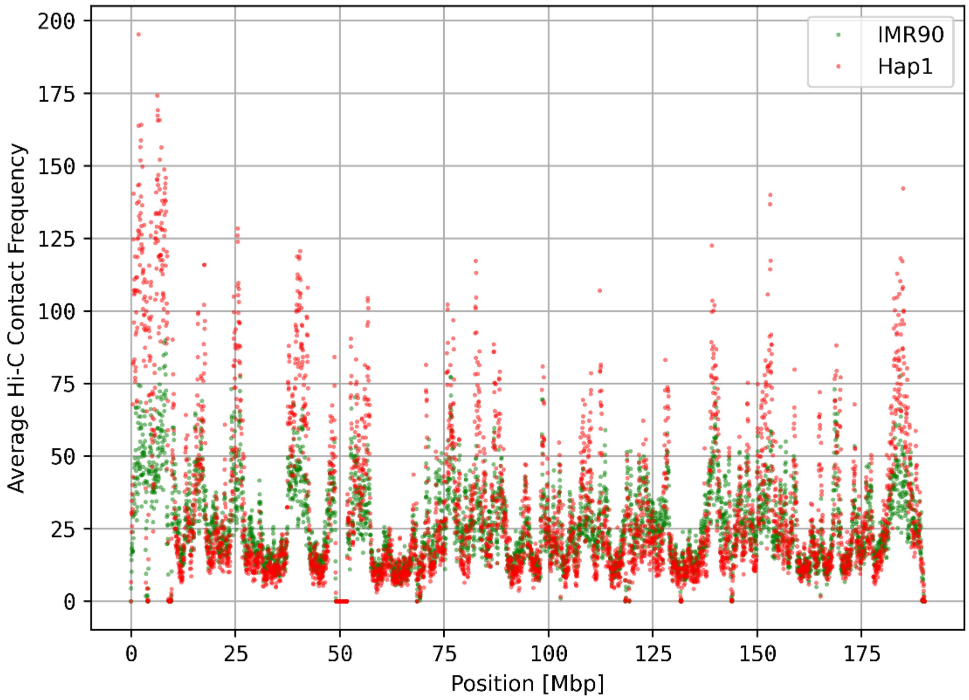

Figure S96: The Hi-C contact frequencies on chromosome 4 for different cell lines: (red) The Hi-C frequencies in HAP1. (green) The Hi-C frequencies in IMR90.

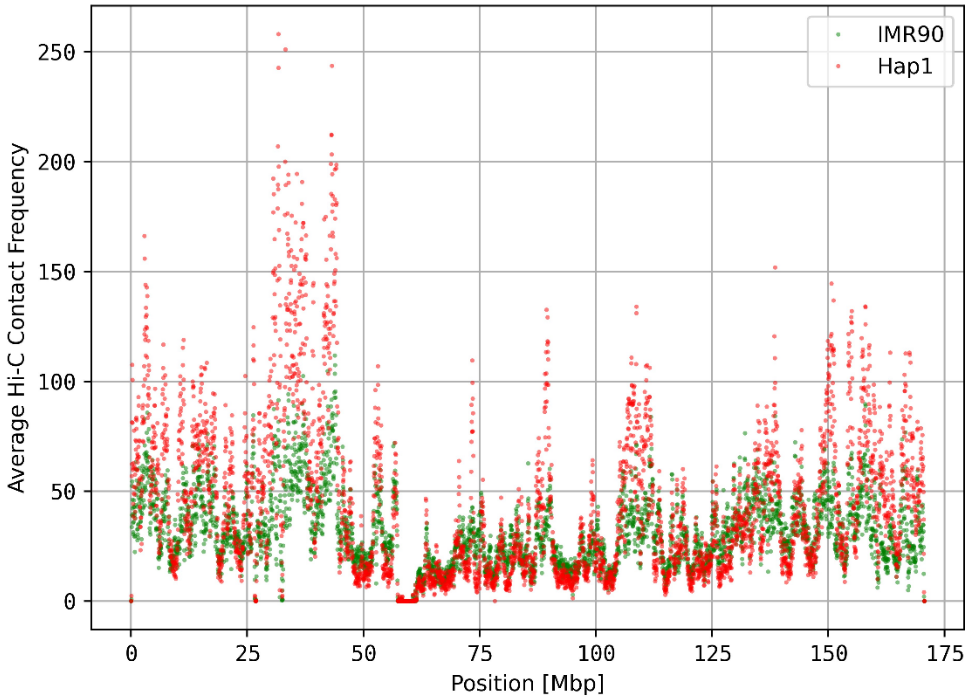

Figure S97: The Hi-C contact frequencies on chromosome 6 for different cell lines: (red) The Hi-C frequencies in HAP1. (green) The Hi-C frequencies in IMR90.

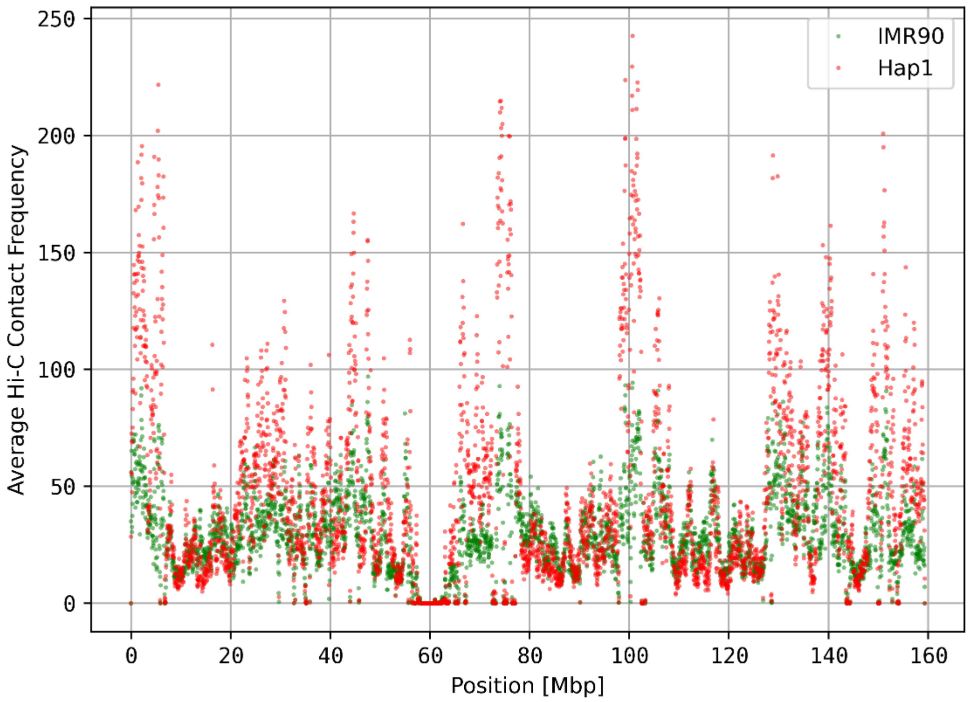

Figure S98: The Hi-C contact frequencies on chromosome 7 for different cell lines: (red) The Hi-C frequencies in HAP1. (green) The Hi-C frequencies in IMR90.

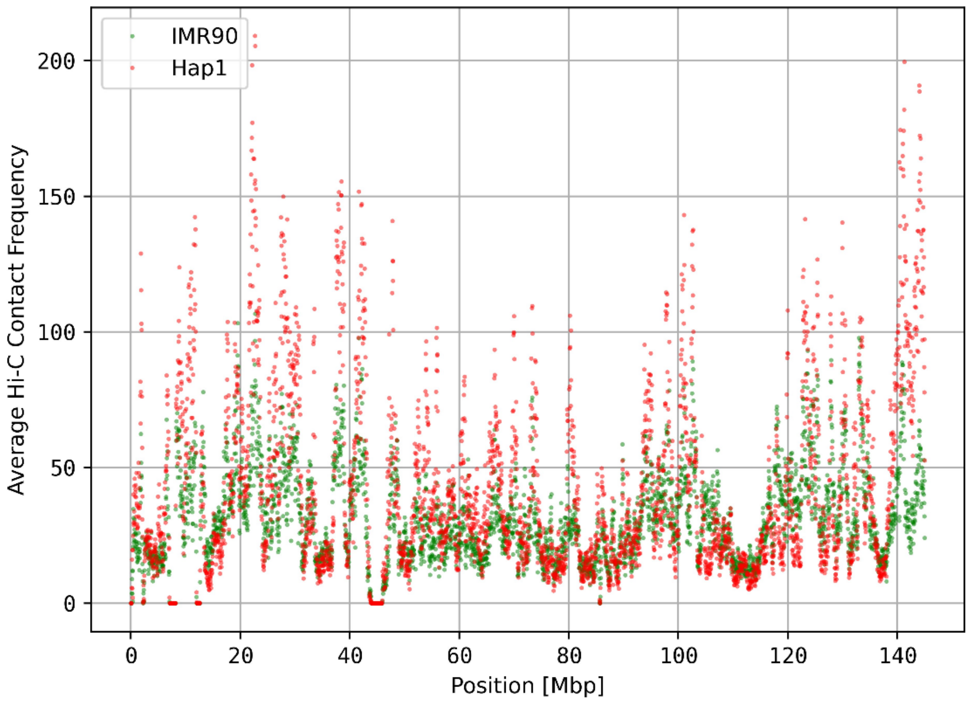

Figure S99: The Hi-C contact frequencies on chromosome 8 for different cell lines: (red) The Hi-C frequencies in HAP1. (green) The Hi-C frequencies in IMR90.

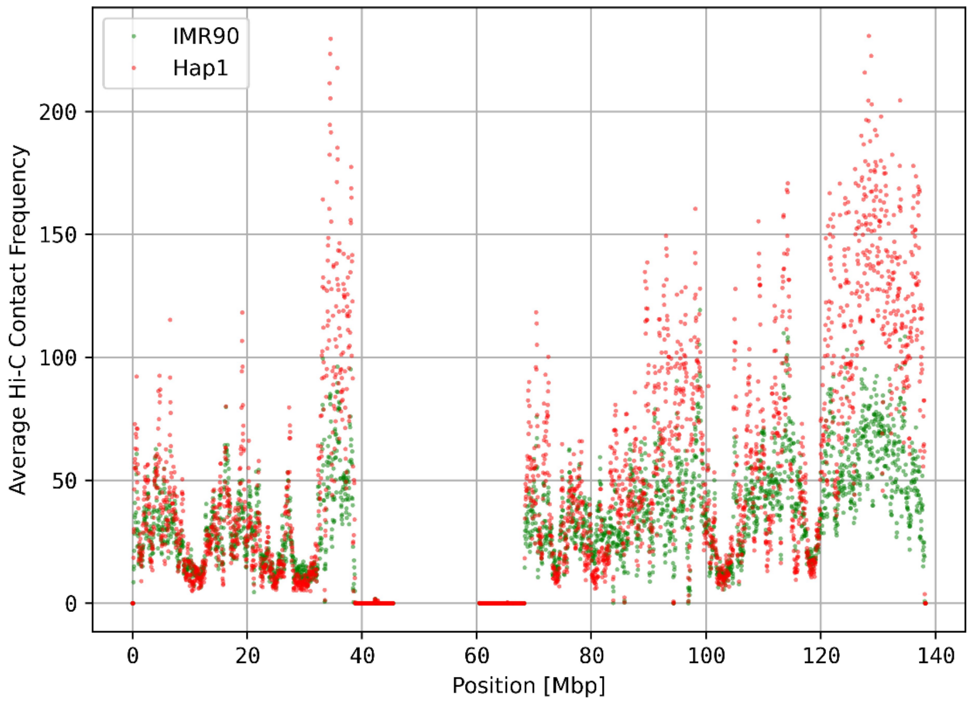

Figure S100: The Hi-C contact frequencies on chromosome 9 for different cell lines: (red) The Hi-C frequencies in HAP1. (green) The Hi-C frequencies in IMR90.

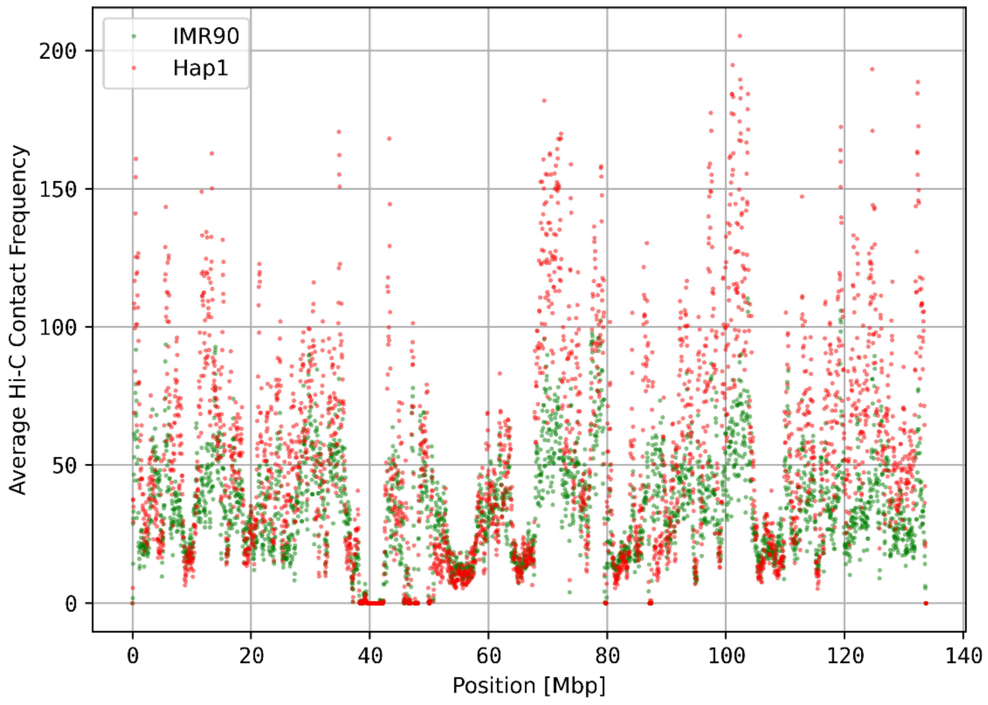

Figure S101: The Hi-C contact frequencies on chromosome 10 for different cell lines: (red) The Hi-C frequencies in HAP1. (green) The Hi-C frequencies in IMR90.

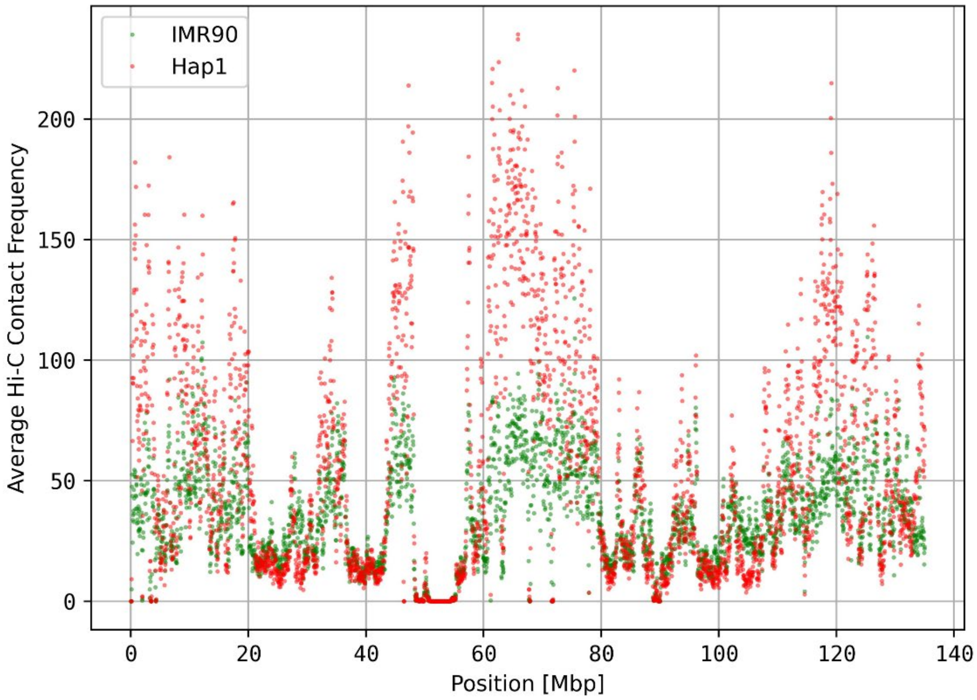

Figure S102: The Hi-C contact frequencies on chromosome 11 for different cell lines: (red) The Hi-C frequencies in HAP1. (green) The Hi-C frequencies in IMR90.

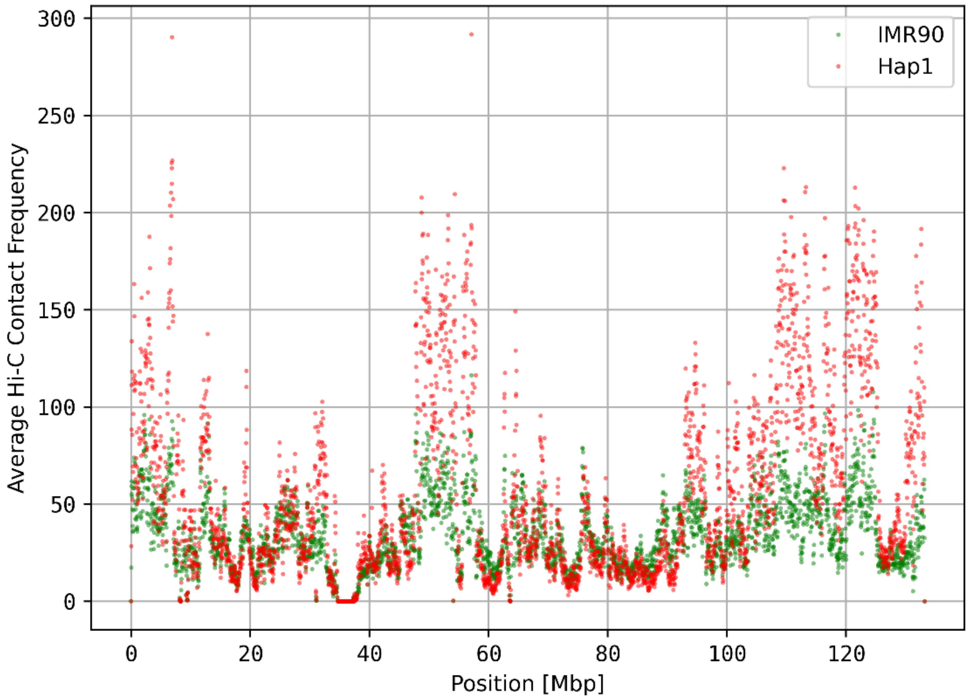

Figure S103: The Hi-C contact frequencies on chromosome 12 for different cell lines: (red) The Hi-C frequencies in HAP1. (green) The Hi-C frequencies in IMR90.

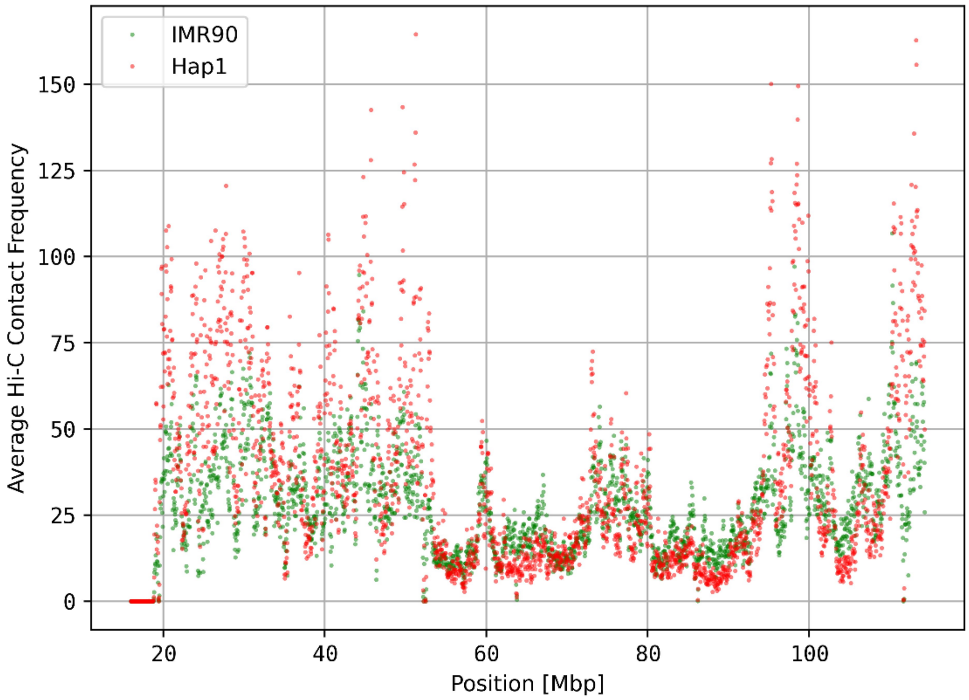

Figure S104: The Hi-C contact frequencies on chromosome 13 for different cell lines: (red) The Hi-C frequencies in HAP1. (green) The Hi-C frequencies in IMR90.

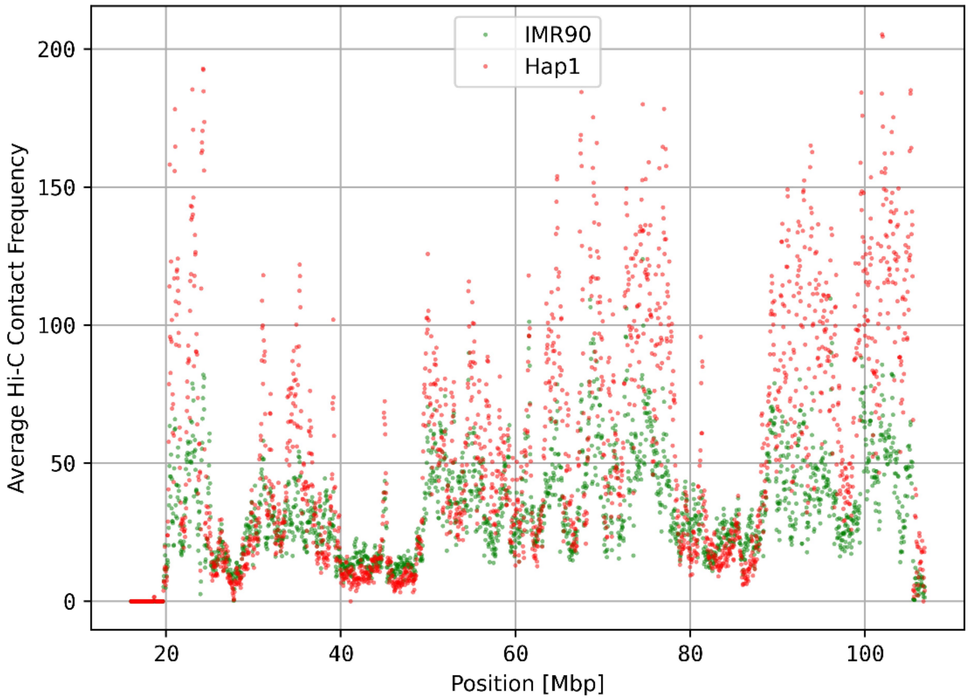

Figure S105: The Hi-C contact frequencies on chromosome 14 for different cell lines: (red) The Hi-C frequencies in HAP1. (green) The Hi-C frequencies in IMR90.

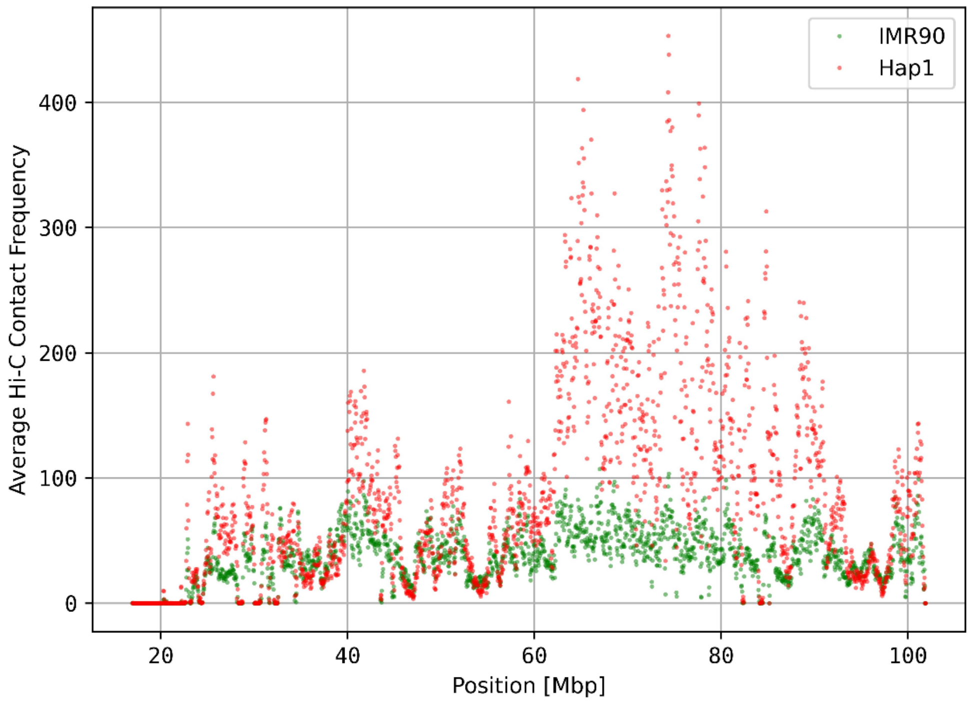

Figure S106: The Hi-C contact frequencies on chromosome 15 for different cell lines: (red) The Hi-C frequencies in HAP1. (green) The Hi-C frequencies in IMR90.

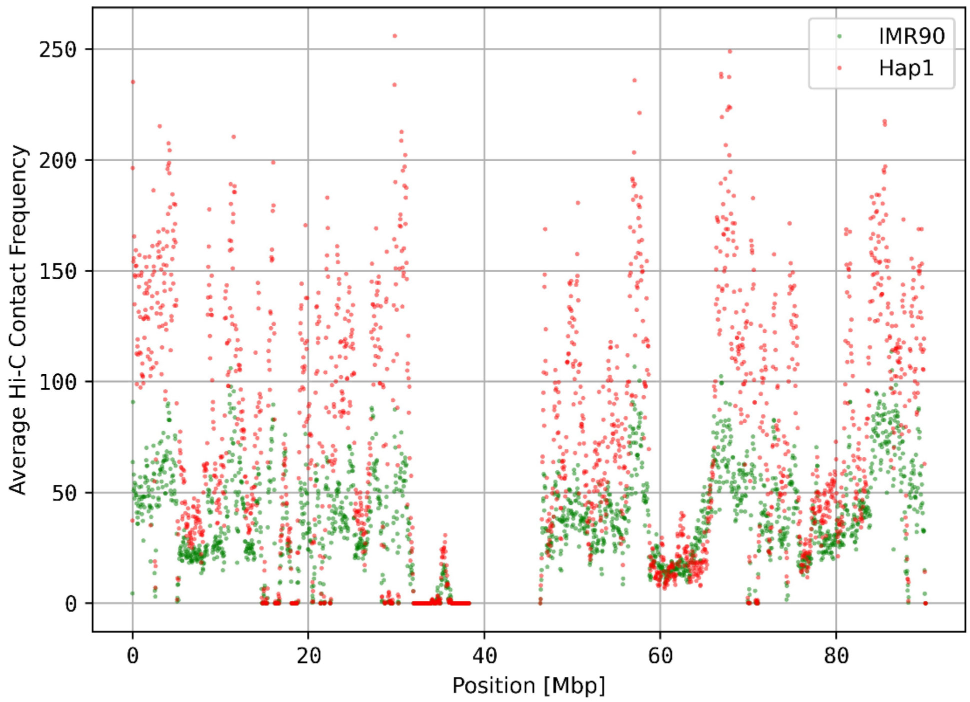

Figure S107: The Hi-C contact frequencies on chromosome 16 for different cell lines: (red) The Hi-C frequencies in HAP1. (green) The Hi-C frequencies in IMR90.

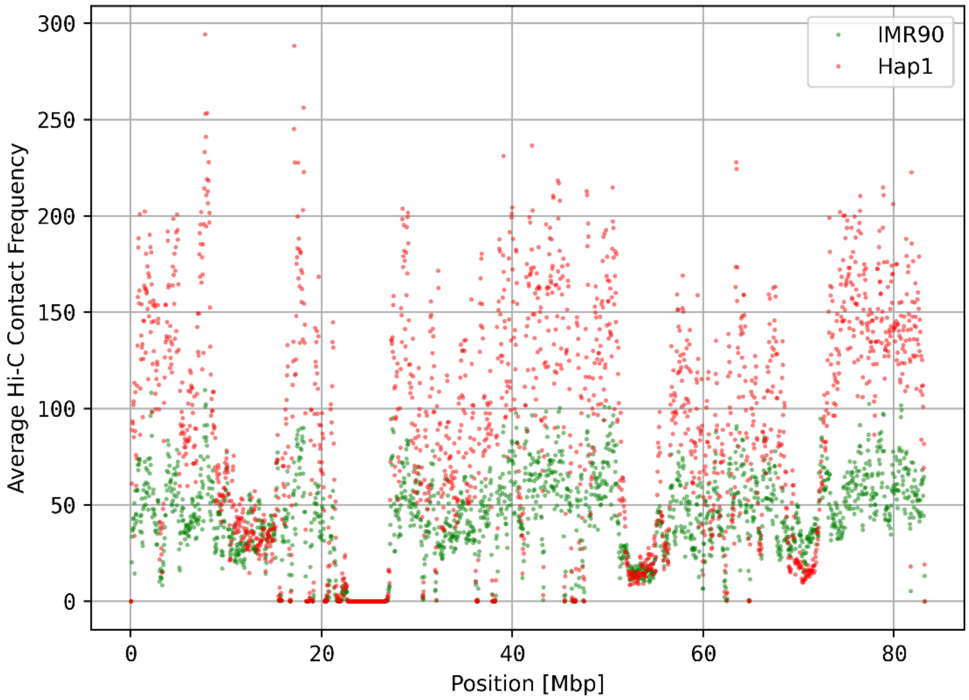

Figure S108: The Hi-C contact frequencies on chromosome 17 for different cell lines: (red) The Hi-C frequencies in HAP1. (green) The Hi-C frequencies in IMR90.

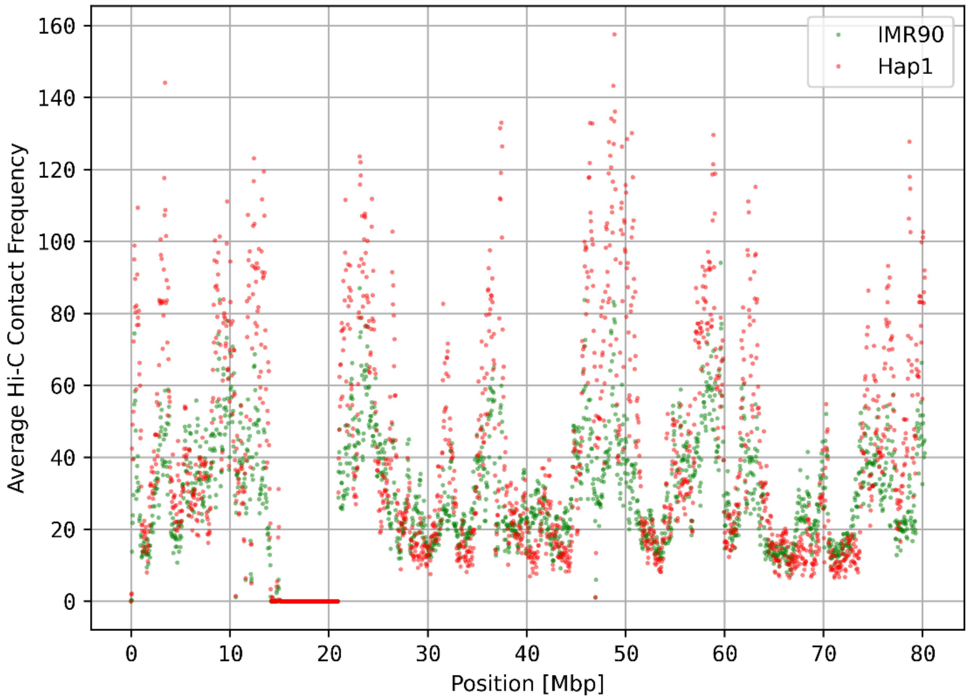

Figure S109: The Hi-C contact frequencies on chromosome 18 for different cell lines: (red) The Hi-C frequencies in HAP1. (green) The Hi-C frequencies in IMR90.

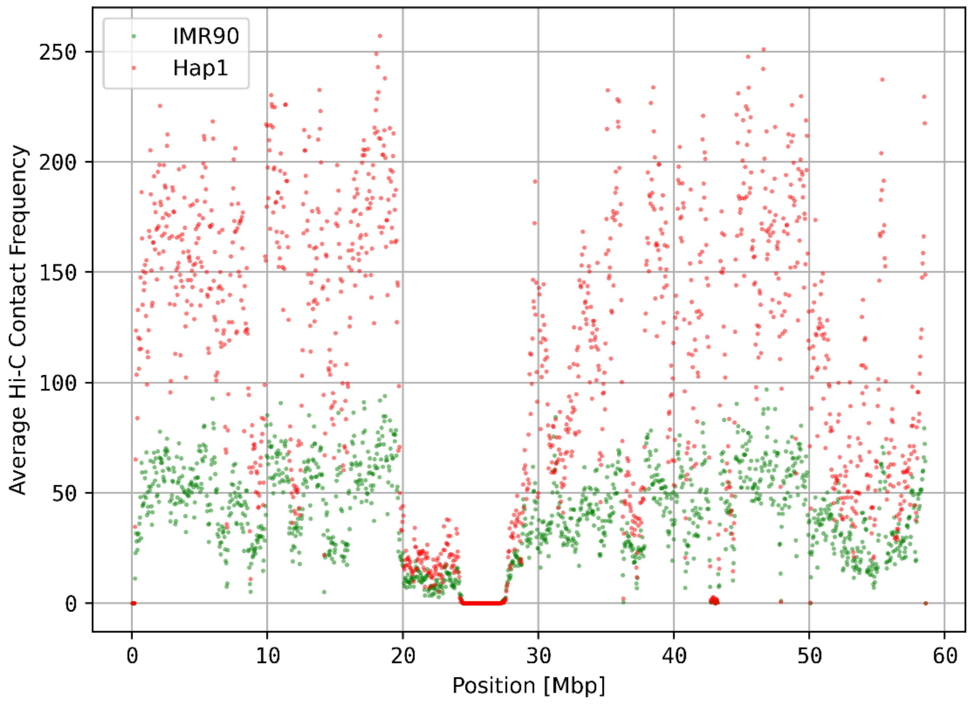

Figure S110: The Hi-C contact frequencies on chromosome 19 for different cell lines: (red) The Hi-C frequencies in HAP1. (green) The Hi-C frequencies in IMR90.

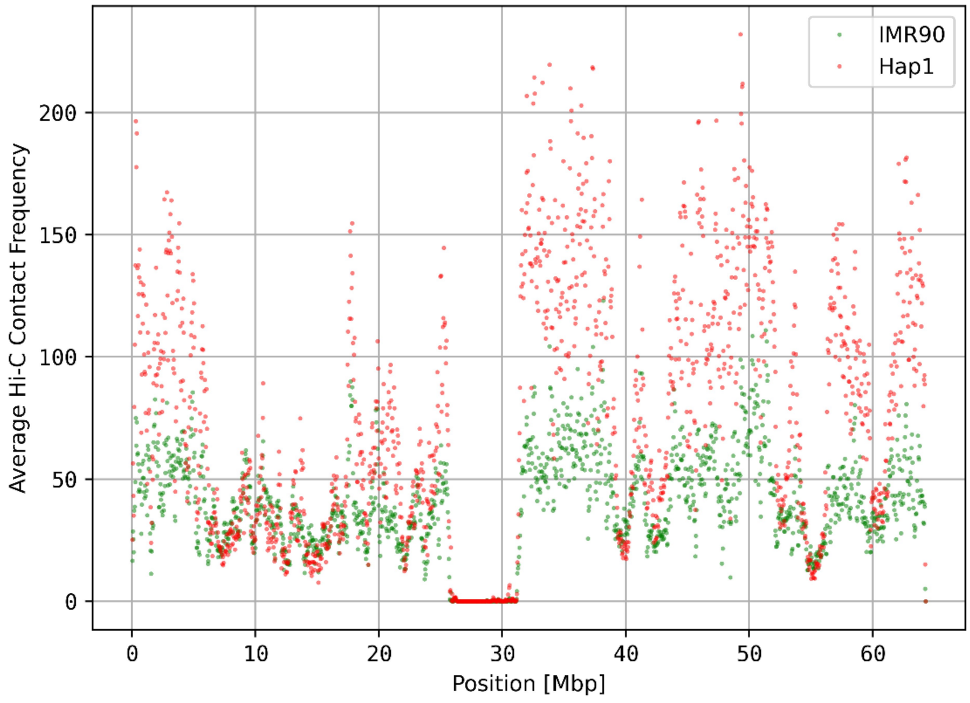

Figure S111: The Hi-C contact frequencies on chromosome 20 for different cell lines: (red) The Hi-C frequencies in HAP1. (green) The Hi-C frequencies in IMR90.

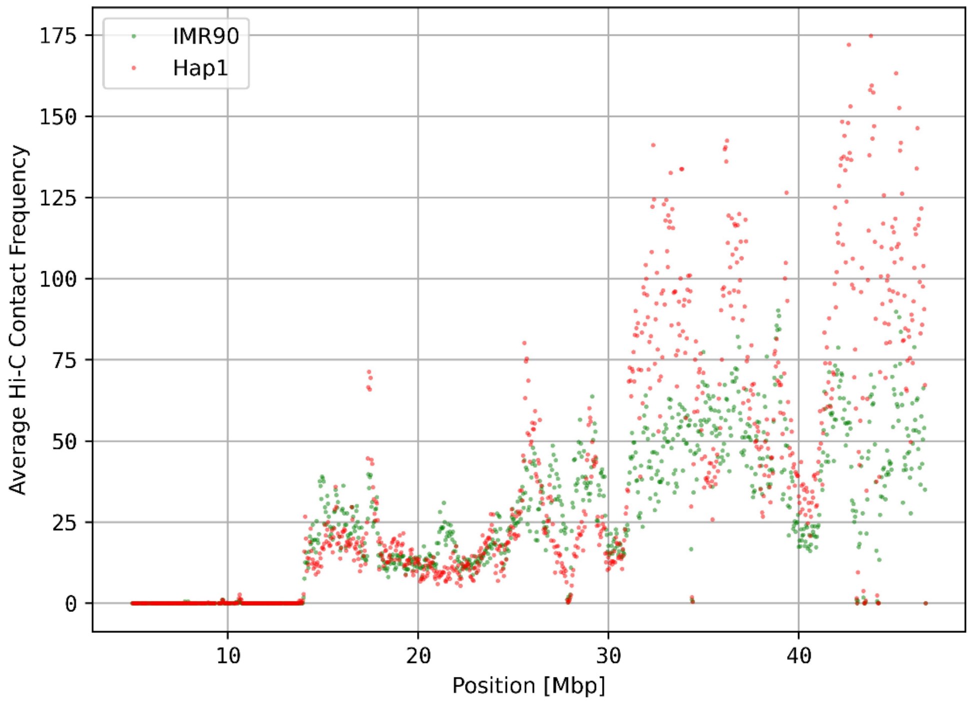

Figure S112: The Hi-C contact frequencies on chromosome 21 for different cell lines: (red) The Hi-C frequencies in HAP1. (green) The Hi-C frequencies in IMR90.

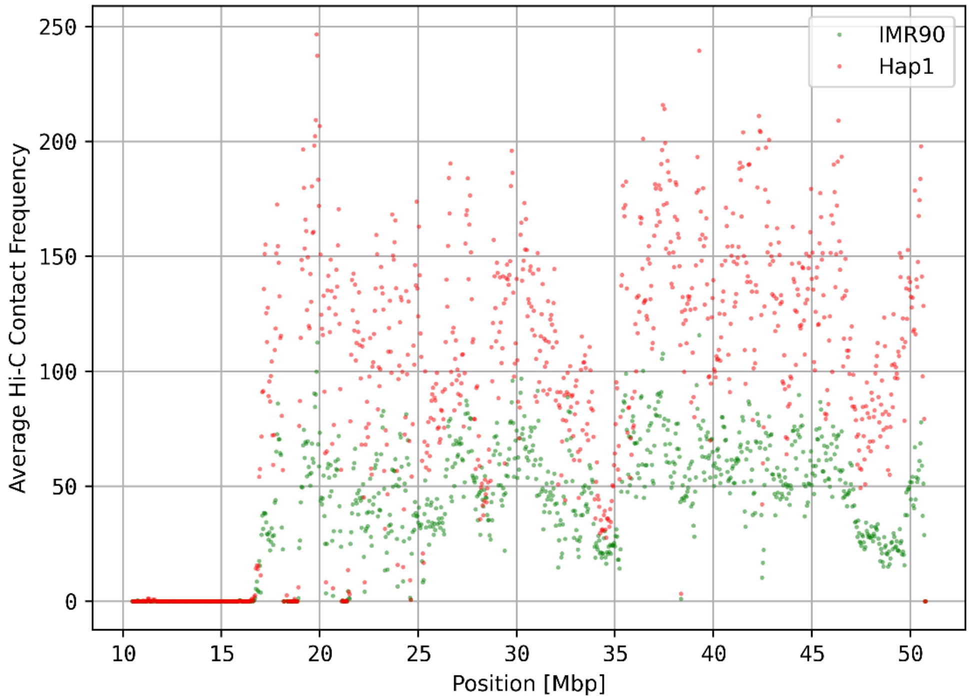

Figure S113: The Hi-C contact frequencies on chromosome 22 for different cell lines: (red) The Hi-C frequencies in HAP1. (green) The Hi-C frequencies in IMR90.

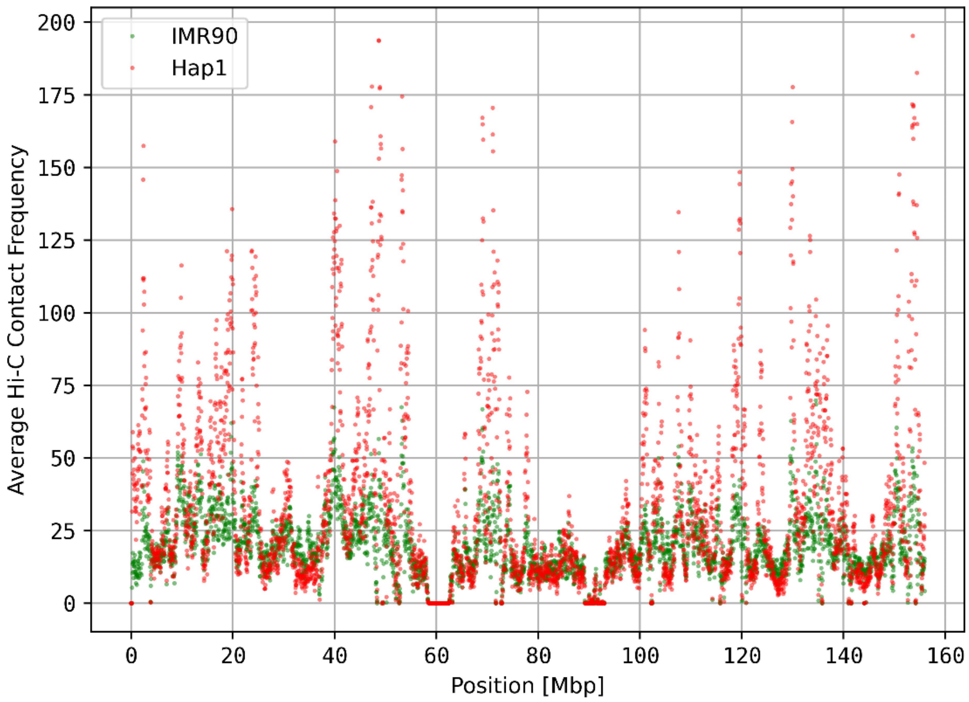

Figure S114: The Hi-C contact frequencies on chromosome X for different cell lines: (red) The Hi-C frequencies in HAP1. (green) The Hi-C frequencies in IMR90.

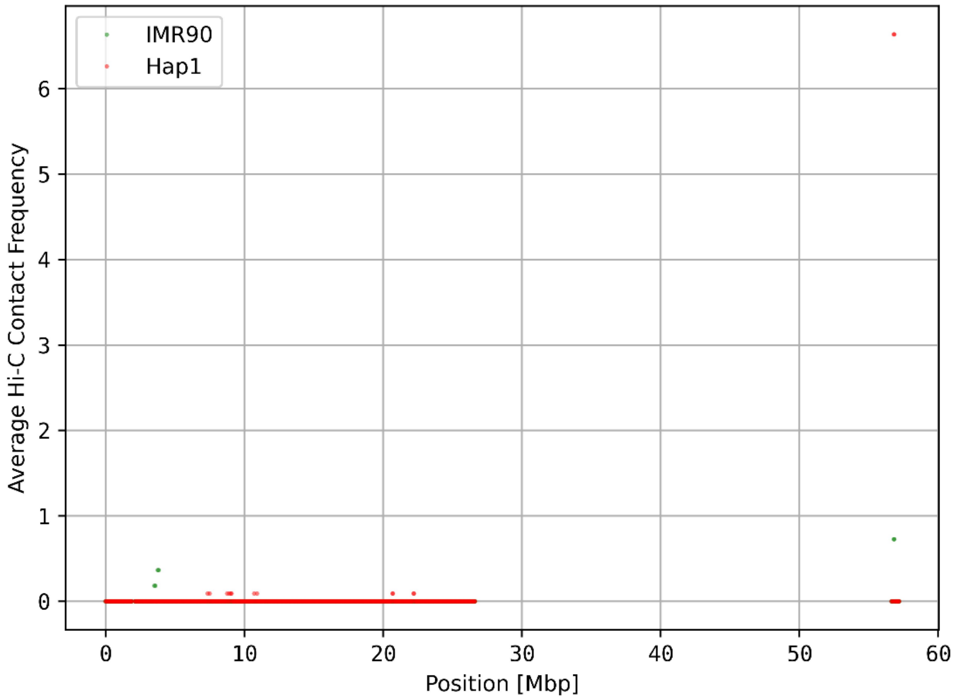

Figure S115: The Hi-C contact frequencies on chromosome Y for different cell lines: (red) The Hi-C frequencies in HAP1. (green) The Hi-C frequencies in IMR90.

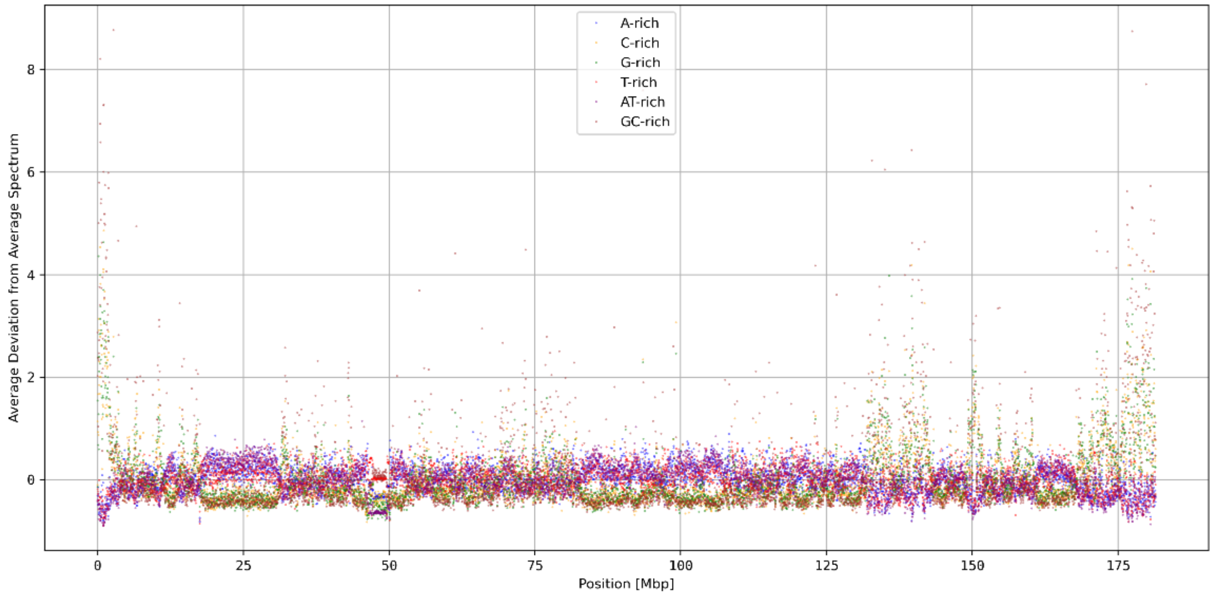

Figure S116: The spectral deviations of segments on chromosome 5 for all word sets
